# Pyridine-boryl radical mediated synthesis of quinolines *via* α-amino radical formation and intramolecular alkenyl sulfone trapping

**DOI:** 10.1039/d6qo00132g

**Published:** 2026-04-10

**Authors:** Raúl Valderrama-Callejón, Inés Alonso, Sara Gómez, David Jiménez, Gonzalo Dorado-Briones, Emily L. Vargas, Mariola Tortosa, M. Belén Cid

**Affiliations:** a Department of Organic Chemistry, Universidad Autónoma de Madrid Cantoblanco 28049 Madrid Spain Belen.cid@uam.es Mariola.tortosa@uam.es; b Institute for Advanced Research in Chemical Sciences (IAdChem), Universidad Autónoma de Madrid 28049 Madrid Spain; c Center of Innovation in Advanced Chemistry (ORFEO-CINQA) Spain; d Sede del Caribe, Universidad de Costa Rica Limón Costa Rica

## Abstract

This study demonstrates that catalytic amounts of functionalized pyridines, in the presence of B_2_nep_2_ as a diboron reagent, can react with imines to form α-amino radicals. These α-amino radicals can be intramolecularly trapped by alkenyl sulfones through a *6-endo-trig* process. According to our experiments and DFT calculations, the sulfonyl moiety plays a crucial role in the cyclization and aromatization processes, which occur in two steps: elimination of the sulfonyl radical and hydrogen atom abstraction, facilitating both aromatization and regeneration of the pyridine–boryl radical. The approach represents a useful application of radical-based methodologies for heterocycle synthesis under mild and catalytic conditions.

## Abstract 2

Pyridine–boryl radicals trigger α-amino radical formation from imines, enabling a 6-*endo-trig* intramolecular trapping with alkenyl sulfones to deliver quinolines under mild conditions.

## Graphical abstract

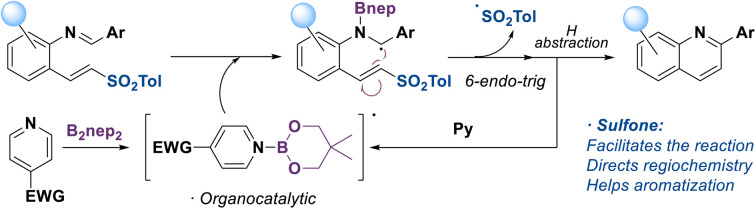

## Introduction

**Scheme 1:**
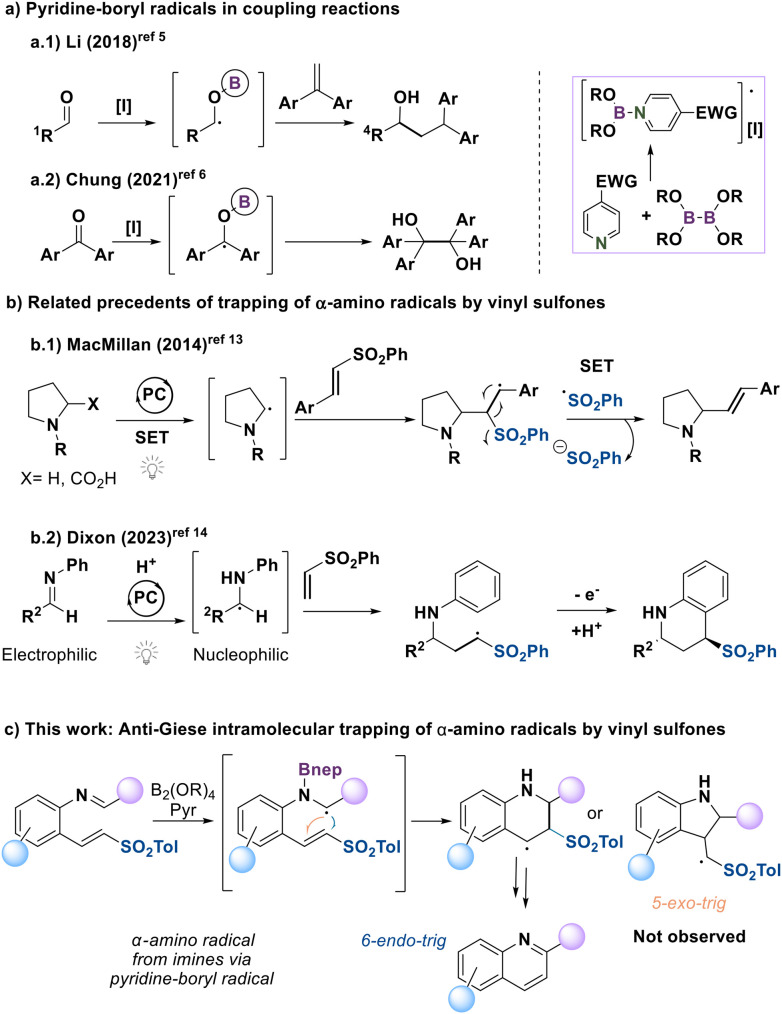
Precedents and present work.

In recent years, diboron reagents have emerged as a powerful tool in different research fields, including organic synthesis, catalysis, and medicinal chemistry, enabling the efficient construction of complex structures due to their unique versatility and interesting reactivity.^1^ As Lewis acids, diboron reagents can undergo selective and controlled transformations.^2^ As evidence of this, diboron compounds react with EWG-substituted pyridines^3^ to generate pyridine-boryl radicals (Scheme 1), which could promote diverse reactions under mild conditions.^4^ For example, pyridine-boryl radicals have been used by Li's research group in the reductive coupling reaction between aldehydes and arylalkenes^5^ as well as Chung's group in the reductive pinacol coupling of diaryl ketones^6^ (Scheme 1a). To our knowledge, the equivalent reaction using imines to form α-amino radicals has not been described.^7^ Combining our experience in boron^8^ and sulfone chemistry,^9,10^ we explored the potential of alkenyl sulfones as acceptors of α-amino radicals in an intramolecular setting,^11,12^ which could provide straightforward access to heterocycles.

There are closely related precedents describing the addition of α-amino radicals—generated through different methodologies—to sulfone-containing systems. In particular, MacMillan has reported an *anti*-Giese-type addition of α-amino radicals to alkenyl sulfones, followed by elimination to afford allylic amines (Scheme 1b.1).^13^ This observed pathway is consistent with stabilization of the corresponding benzylic radical. In contrast, Dixon's work involves a classical Giese addition that ultimately enables the synthesis of tetrahydroquinolines.^14^ These examples highlight the divergent reactivity of α-amino radicals with alkenyl sulfones and suggest that the intramolecular variant could open new synthetic opportunities.

Specifically, the intramolecular trapping of imines with alkenyl sulfones *via* α-borylamino radicals—generated upon treatment with pyridine-boryl radicals—could undergo cyclization through either a 6-*endo-trig* or a 5-*exo-trig* pathway (Scheme 1c).^15^ We envisioned that radical trapping *via* a 6-endo-trig process could be possible by benzylic stabilization, while the sulfonyl moiety could facilitate ring aromatization through elimination, ultimately enabling the one-pot synthesis of quinolines.

The importance of quinolines in fields ranging from pharmacology^16^ to materials science^17^ has driven the development of numerous synthetic methodologies, making this an area of great relevance and dynamism within organic chemistry, as evidenced by the growing number of publications on the subject.^18^ Among the reported approaches, only recently—mainly due to the rapid development of photoredox catalysis—has the reactivity of radical intermediates been exploited for quinoline synthesis, including strategies based on iminyl and imidoyl radical cations^19^ and α-amino radicals.^20^

We present herein a radical cascade cyclization protocol to prepare quinolines, using pyridine-boryl radicals as sustainable organic promoters of α-amino radicals from imines,^21^ and versatile alkenyl sulfones as radical acceptors.^22,23^

## Results

Quinoline precursors 1 bearing an imine and alkenyl sulfone moieties are stable and easy to prepare from 2-aminobenzaldehydes by Horner–Wadsworth–Emmons^24^ reaction and condensation with aldehydes.^25,26^ We started our study using 1a as a model substrate and optimized the reaction conditions by varying the diboron source (B1–B4), the pyridine organocatalyst (P1–P7), and solvents of different polarities and boiling points (Table 1). The reaction was initially tested using 1 equiv. of B_2_nep_2_ (B1) as the boron source and bispyridine P1 in solvents of different polarities.^24^ Among them, toluene provided the highest yield (entry 5), whereas more polar solvents such as DMF and DMSO-d_6_ (entries 2 and 3) resulted in significantly lower conversions or no reaction. Acetonitrile (entry 1) showed similar efficiency, while xylene (entry 4) led to a moderate decrease in yield compared to toluene.

**Table 1:** Optimization conditions of the cyclization reaction

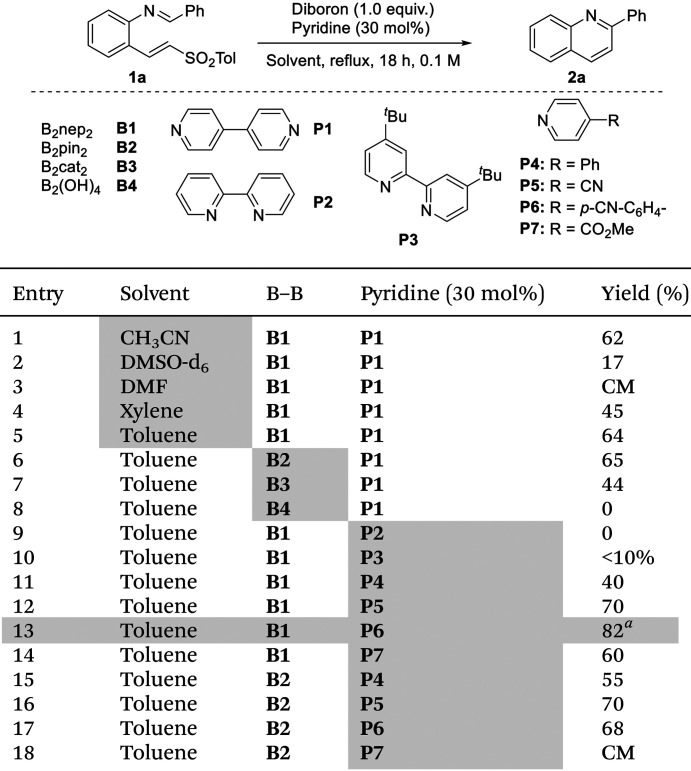

a Isolated yield.

The evaluation of different diboron reagents (B2–B4) revealed that B_2_pin_2_ (B2) produced a very similar yield to B_2_nep_2_ (B1) when combined with P1 (entry 6 *vs*. entry 5). In contrast, the use of B_2_cat_2_ (B3) led to a significantly lower yield (entry 7), while B_2_(OH)_4_ (B4) was completely ineffective (entry 8). A range of pyridine derivatives (P2–P7) was then examined using B_2_nep_2_ (B1) as the diboron reagent (entries 9–14). P2 and P3 proved ineffective or poorly effective, giving no conversion or only trace amounts of product (entries 9 and 10). Pyridines P5–P7 significantly improved the reaction outcome, with P6 emerging as the optimal catalyst and affording an isolated yield of 82% (entry 13). The behaviour of B_2_pin_2_ (B2) in combination with pyridines P4–P7 ^24,27^ was also evaluated (entries 15–18). Overall, the results were comparable but generally lower or less consistent than those obtained with B_2_nep_2_. For pyridine P5, the yields obtained with the two diboron reagents were comparable (entries 12 and 16).

Lower amounts of the diboron reagents resulted in decreased yields, as will be discussed in the mechanistic studies (see Table 3 of the manuscript). However, decreasing the catalytic loading of the pyridine derivative to 20%, 10%, or 5% completely suppressed product formation.^24^

For scope analysis, B_2_nep_2_ was primarily used; however, it is important to highlight that B_2_pin_2_ also provided competitive results. Therefore, B_2_pin_2_ could be a viable alternative for mechanistic studies, facilitating comparisons with literature data.^5^

We explored the reaction with various substituents on the aromatic ring of the imine (R) and on the 2-aminobenzaldehyde moiety (*Z*) (Table 2). We first examined the effect of an *ortho*-methyl substituent to evaluate the limitations imposed by steric hindrance. Using imine 1b, we tested B_2_pin_2_ and B_2_nep_2_, obtaining yields of 33% and 59% respectively. This indicates that the more sterically hindered B_2_pin_2_ leads to a lower yield.

**Table 2:** Scope of the cyclization reaction

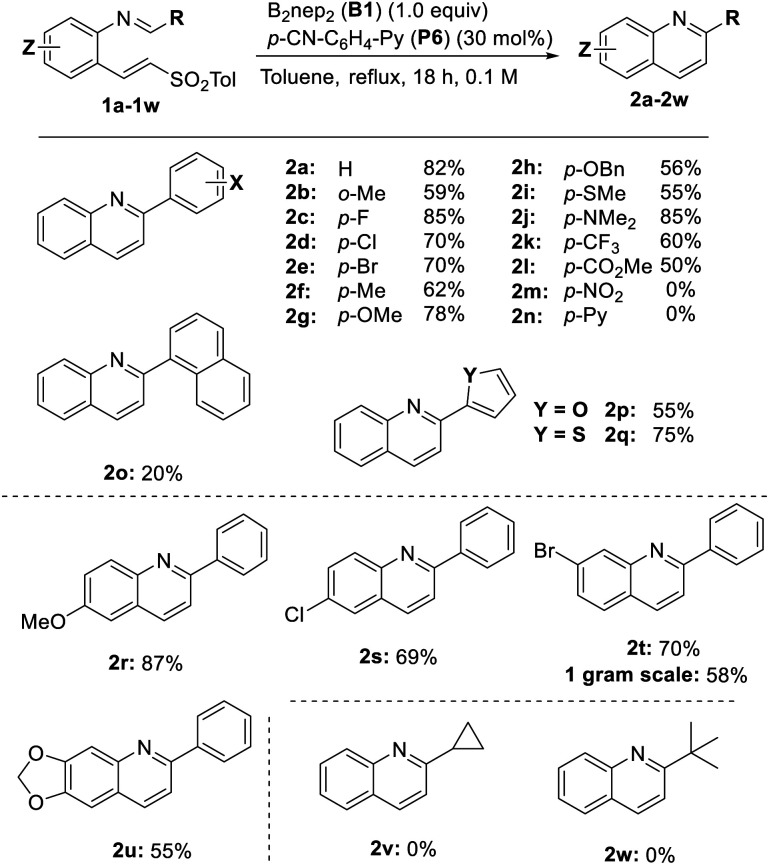

The method tolerates a range of electron-donating and electron-withdrawing groups at the para position of a phenyl ring (1c–1l), with yields ranging from 50% (2l) to 85% (2c, X = F, and 2j, X = *p*-NMe_2_). Quinoline derivatives are also formed when heterocyclic substituents such as furan and thiophene are present (2p and 2q). A 1-naphthyl substituent leads to lower yields, probably due to steric hindrance. However, as expected, pyridines (1n) are not compatible, likely due to their potential to react with the diboron reagent. Similarly, the nitro derivative (1m) undergoes reduction under the reaction conditions.^28^ The presence of different substituents on the 2-aminobenzaldehyde moiety (*Z*) are well tolerated, including ether groups (2r and 2u), as well as chlorine and bromine substituents at different positions, allowing for further functionalization (2s and 2t). Substrates containing cyclopropyl or *tert*-butyl groups (2v and 2w) proved unreactive under the standard conditions. Other aliphatic aldehydes do not afford the corresponding imines and instead give complex mixtures, likely due to the presence of enolizable α-protons.

Notably, compound 2t was successfully synthesized on a 1-gram scale in 58% yield. This brominated quinoline is a valuable intermediate that has been used before for the synthesis of linsitinib.^29^

### Mechanistic studies

All experiments designed to elucidate the reaction mechanism were performed under a defined set of standard conditions, using B_2_pin_2_ (B2) as the diboron reagent and *p*-cyanopyridine (P5) as the pyridine catalyst, unless explicitly stated otherwise. These standard conditions were used as a reference because both reagents have been previously employed in related mechanistic studies.^5^Table 3 summarizes the variables analyzed, including the role of light, radical scavengers, the necessity of each reagent, the potential autocatalysis by the resulting quinoline and the required amount of diboron reagent.

**Table 3:** Experimental studies on the cyclization mechanism

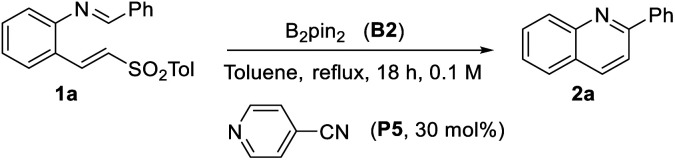		
Entry	Difference with standard conditions	Yield (%)
1	No change	70
2	Without light	70
3	Addition of galvinoxyl (3 equiv.)	—
4	Without P5	—
5	Without B_2_pin_2_	—
6	Using 2a (30 mol%) instead of P5	—
7	Using B_2_pin_2_ (0.6 equiv.)	72
8	Using B_2_nep_2_ (0.6 equiv.) instead of B_2_pin_2_	64
9	Using B_2_nep_2_ (0.3 equiv.) and P1 (30 mol%)	22
10	Using B_2_pin_2_ (0.3 equiv.) and P1 (30 mol%)	19

Performing the reaction in the absence of light resulted in no observable difference, indicating that light is not required (compare entries 1 and 2). However, when galvinoxyl was introduced as a radical inhibitor, the reaction was completely suppressed, and the starting imine was fully recovered, suggesting the radical nature of the mechanism (entry 3). In this case, B_2_pin_2_ was consumed, and a signal at 22.6 ppm (^11^B NMR) is observed, likely indicating that boron has been sequestered by the oxygen atom of galvinoxyl.^30^ The reaction did not proceed without pyridine P5 (entry 4), highlighting its essential role as organocatalyst,^3^ or without B_2_pin_2_ (entry 5). Furthermore, no autocatalysis was observed, as quinoline 2a failed to promote the reaction (entry 6).^24^ Interestingly, the reaction also proceeded with lower amounts of the diboron reagents (0.6 equiv.), both B_2_pin_2_ and B_2_nep_2_ (entries 7 and 8). Nevertheless, the use 0.3 equiv. of diboron reagents using P1 as pyridine provides lower yields (entries 9 and 10).

**Scheme 2:**
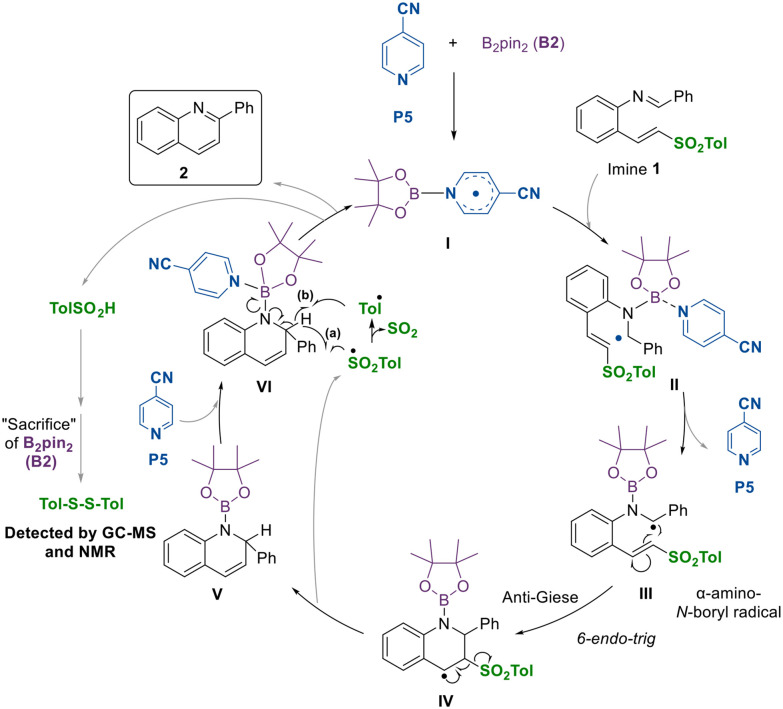
Mechanistic proposal.

Based on our observations, we propose a catalytic cycle, illustrated in Scheme 2, and exemplified using *p*-cyanopyridine (P5) and B_2_pin_2_ (B2). The cycle begins with the nucleophilic attack of the pyridine derivative on the diboron compound, leading to the formation of the boron–pyridine radical intermediate I. Next, this intermediate would react with imine 1 by forming the N–B bond to give complex II which, by releasing a molecule of pyridine would form the α-amino radical III, that would be captured by the olefin in a 6-*endo-trig* process. The benzylic radical IV would evolve to species V due to the presence of the *p*-tolylsulfonyl group, which acts as a good leaving group and is released as a radical, thereby facilitating the formation of the double bond. Catalytic pyridine (P5) may enter the catalytic cycle by coordinating to boron to form pyridine–boron species VI. Subsequently, the released sulfonyl radical would abstract a hydrogen to form TolSO_2_H, producing aromatization to quinoline 2 and the homolytic cleavage of the nitrogen–boron bond. This step may proceed through either pathway (a) or pathway (b), as discussed below. This process would regenerate species I, allowing it to re-enter the catalytic cycle.

It is known that sulfinic acids predominantly evolve into the corresponding disulfides.^31^ When we monitored the reaction, we detected signals corresponding to the PinB–O–BPin species in the ^11^B NMR spectrum (at 22.4 ppm).^24,32^ The presence of this species suggests that the diboron reagent has been involved in a deoxygenation process.^24^ This is consistent with the detection of *p*-tolyl disulfide in both the ^1^H NMR and the mass spectra, which may have formed from the oxygenated by-products of the sulfone. Nevertheless, the amount of *p*-tolyl disulfide observed by ^1^H NMR integration only account for 33% of the starting sulfone. Therefore, while the presence of PinB–O–BPin could indicate that part of the diboron reagent is sacrificed to deoxygenate the S–O bond,^33^ this is probable not the only operative pathway in the aromatization process. The *p*-tolylsulfonyl radical could also evolve by losing SO_2_, generating the tolyl radical, which may play the same role as the sulfonyl radical depicted in Scheme 2, that is, abstracting the hydrogen from intermediate V and allowing the catalytic cycle to continue (b).^34^ In this case, toluene would be formed as a side product, but its presence would go unnoticed.

**Fig. 1:**
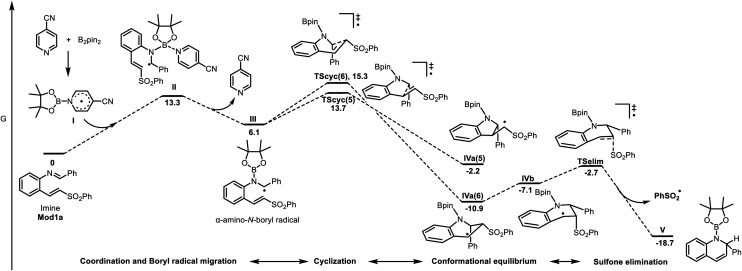
Energy profile in toluene [M06-2X_SMD_/6-311++G(d,p)//M06-2X/6-31G(d). Relative *G* values at 298 K (kcal mol^−1^)].

To better understand the mechanism and the origin of the selectivity of this cyclization, DFT calculations were performed using B_2_pin_2_ (B2) and *p*-cyanopyridine (P5) as model compounds due to existing computational precedents for intermediate I.^5,35^ Additionally, imine mod1a in which the tolyl group was replaced by a phenyl to simplify the calculations, was used as a model for imine 1a. According to the energy profile shown in Fig. 1, the cyclization of intermediate III through a five-membered cycle transition state [TScyc(5)] is slightly more favorable (1.6 kcal mol^−1^) than through the six-membered cycle [TScyc(6)], which agrees with Baldwin rules.^15^ However, the cyclic radical obtained IVa(5), only stabilized by the sulfonyl group, is less stable (8.7 kcal mol^−1^) than the benzyl 6-membered cycle radical IVa(6), which after a conformational change to IVb, easily evolves into intermediate V by releasing the sulfonyl radical. The equivalent process from IVa(5) would not afford a stable intermediate, likely favoring the reverse process.

When the reaction mixture was analyzed by mass spectrometry, the mass of the analogue of either IVa(5) or IVa(6) after hydrogen atom transfer (HAT) was observed with higher intensity after 1 hour than after 18 hours. Although we acknowledge that MS peak intensities are not quantitative due to differences in ionization efficiency and possible ion-suppression effects, this observation could suggest that IVa(5) might be a transient intermediate that gradually evolves over time.^24,36^ This is consistent with the proposed reversibility of the mentioned process. Thus, the observed product corresponds to the thermodynamic control reaction, and the driving force of the process would be probably the fast elimination of the sulfonyl radical, which finally evolves towards an aromatic compound.

Overall, these computational results are fully consistent with the key role of the sulfonyl moiety in guiding both the cyclization and the subsequent aromatization. We reckon that the sulfonyl group increases the electrophilicity of the alkenyl fragment through a strong −I effect, thereby facilitating radical addition and lowering the barrier for cyclization to give a stabilized benzyl radical. The subsequent elimination of the sulfone, which forms the corresponding sulfonyl radical, is calculated to be highly exergonic, making the process effectively irreversible. This aromatization step constitutes the driving force that pushes the reaction forward and accelerates overall product formation.

## Conclusions

This original strategy opens a different approach to heterocyclization methods, demonstrating the effectiveness of functionalized pyridines as organocatalysts to activate diboron reagents and, in turn, to form α-amino radicals from imines. Additionally, it shows that sulfones make the reaction possible, can control the regiochemistry of the process, favour the aromatization and contribute to recovering the pyridine-boryl radical. Therefore, this strategy provides a useful platform for the synthesis of heterocycles, with quinolines as the first accessible model and potential extension to more complex structures.

## Conflicts of interest

There are no conflicts to declare.

## Supplementary Material

QO-013-D6QO00132G-s001

## Data Availability

The data supporting this article have been included as part of the supplementary information (SI). Supplementary information is available. The Supplementary Information includes full experimental procedures, characterization data for all new compounds, NMR spectra, additional control experiments, and computational details. See DOI: https://doi.org/10.1039/d6qo00132g.
