# Supplementary material for: Pyridine-boryl radical mediated synthesis of quinolines via α-amino radical formation and intramolecular alkenyl sulfone trapping
Source: Org Chem Front. 2026 Apr 10;13(10):3208–14. doi: 10.1039/d6qo00132g (PMC13068079; doi:10.1039/d6qo00132g)
Supplement: QO-013-D6QO00132G-s001 [file QO-013-D6QO00132G-s001.pdf]

Supplementary Information (SI) for Organic Chemistry Frontiers.
This journal is © the Partner Organisations 2026

Supporting information for

Pyridine-boryl radical mediated synthesis of quinolines via α-amino radical formation and intramolecular vinyl sulfone trapping

Raúl Valderrama-Callejón,[a] Inés Alonso,[a, b, c] Sara Gómez,[a] David Jiménez,[a] Gonzalo Dorado-Briones,[a] Emily L. Vargas,[d] Mariola Tortosa,* [a, b, c] M. Belén Cid* [a, b]

[a] Department of Organic Chemistry, Universidad Autónoma de Madrid, Cantoblanco, 28049 Madrid (Spain). e-mail: belen.cid@uam.es, mariola.tortosa@uam.es

[b] Institute for Advanced Research in Chemical Sciences (IAdChem), Universidad Autónoma de Madrid, Cantoblanco, 28049 Madrid (Spain)

[c] Center of Innovation in Advanced Chemistry (ORFEO-CINQA).

[d] Sede del Caribe, Universidad de Costa Rica, Limón, Costa Rica

Table of contents

1.- General remarks S2

2.- General synthetic route and synthesis of precursors S3

3.- Synthesis of imines S9

4.- Optimization of the quinoline formation S21

5.-Formation of quinoline via cyclization reaction using diboron reagents and imines S23

6.- Mechanistic studies S30

7.- Experimental spectra S36

8.- Theoretical calculations S77

References S86

1.- General Remarks

NMR spectra were acquired at room temperature using the Brucker AV-V06 equipment (300 MHz for ^{1}H, 75 MHz for ^{13}C, 96 MHz for ^{11}B and 282 MHz for ^{19}F), in $CDCl_3$, C_6D_6, DMSO-d_6 or CD_2Cl_2 as it will be indicated in every case. Chemical shifts (δ) are reported in ppm relative to residual solvent signal ($CDCl_3$ 7.26 ppm for ^{1}H-NMR and 77.0 ppm for ^{13}C-NMR, C_6D_6 7.16 ppm for ^{1}H-NMR and 128.6 ppm for ^{13}C-NMR, DMSO-d_6 2.54 ppm for ^{1}H-NMR and 39.5 ppm for ^{13}C-NMR, CD_2Cl_2 5.32 ppm for ^{1}H-NMR and 53.8 ppm for ^{13}C-NMR). In all ^{1}H-NMR spectra the multiplicity is indicated: s (singlet), d (doublet), t (triplet), q (quartet), m (multiplet) and the values of coupling constants (J) in Hertz (Hz).

All reactions were carried out under an inert atmosphere with argon unless contrary is indicated.

The reactions were monitored by thin layer chromatography (TLC) on silica gel type 6 F254 chromatofoils, with layer thickness of 0.2 mm (Merck). The analysis of chromatographic plates was carried out with ultraviolet light lamp with a wavelength of 254/365 nm, or solutions of phosphomolybdic acid, ninhydrin, potassium permanganate or ammonium and cerium (IV) nitrate.

Separation and purification were performed by flash chromatography using silica gel 60 with a particle size of 230-400 mesh (Merck) as stationary phase. The eluent used is specified in each case, as well as the ratio of solvents.

Mass spectrometry analysis was performed using gas chromatography coupled to high-resolution mass spectrometry trials (GC-Q-TOF). The equipment used was a Bruker 436-GC gas chromatograph and a Bruker Compact spectrometer with a Q-TOF analyzer and GC-APCI ionization source and Nitrogen (N_2) as the carrier gas for the chromatograph. The chromatographic method was as follows: injector temperature: 250 ^{o}C, split ratio: 1:100, injection volume: 1 µL, oven temperature ramp: time 0 min: 70 ^{o}C, time 1 min: 70 ^{o}C, time 10 min: 300 ^{o}C, time 15 min: 300 ^{o}C. The mass spectrometer registration range was from 50 to 1000 m/z, with a GCAPCI ionization source temperature of 200 ^{o}C. MS and MS/MS spectra were alternately recorded using the broad banding collision-induced dissociation (bbCID) fragmentation mode. Each chromatogram was internally calibrated using the polysiloxane signals from the column bleeding at the end of each registration.

2.- General synthetic route and synthesis of precursors

General remarks

The general scheme to form the corresponding imines is resumed below.

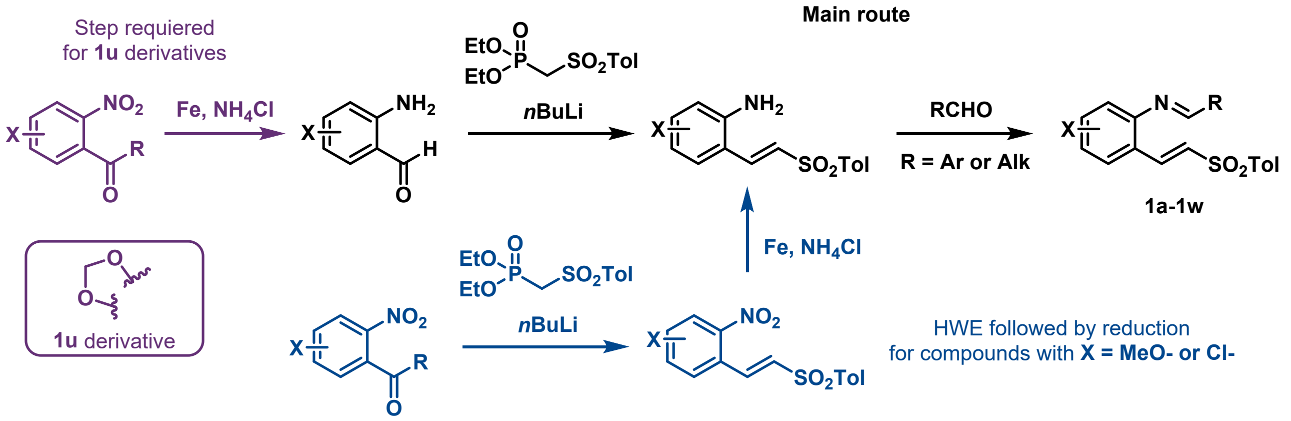

To prepare the corresponding starting materials, the main synthetic route (**highlighted in black**) was established and followed for the majority of the compounds (from **1a** to **1q**, **1v**, and **1w**). However, for three compounds (**1r**, **1s**, and **1u**), the corresponding amines were not commercially available, and therefore the nitro derivatives were employed instead.

For compound **1u**, a prior reduction of the nitro group was performed before entering the main route (step **highlighted in purple**). In contrast, for compounds **1r** and **1s**, the Horner–Wadsworth–Emmons (HWE) reaction had to be carried out first, followed by reduction (**highlighted in blue**). From that point, the synthesis continued along the main route at the imine formation step. This modification of the route was necessary, as performing the reduction first and then proceeding with the main route did not result in alkene formation

In the next page, the starting materials have been described, starting with the HWE reaction and then, the nitro reduction. To check the specific route for each compound, this page must be revised.

To make easier the identification of compounds, all the precursors have been identified with the code **SMX**, where X is a number between 1 and 8.

2.1.-Horner-Wadsworth-Emmons reaction (HWE)

General procedure for HWE

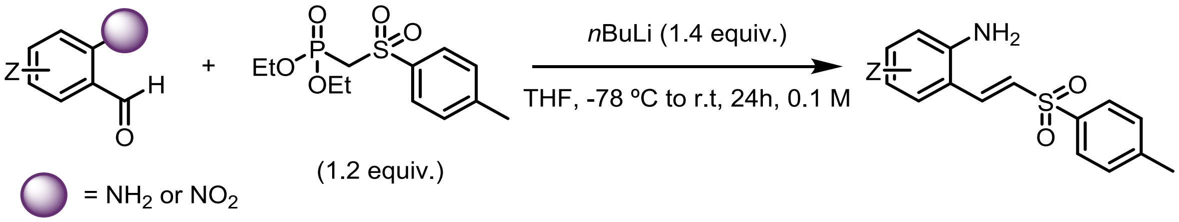

The compound was synthesized following the procedure described in literature.[1] In a round-bottom flask, the phosphonate was added and the flask was evacuated and backfilled with argon three times. The phosphonate was dissolved in dry THF and the *n*BuLi (2.5 M in hexanes) was added at -78 ºC. After 30 min, corresponding amine or nitro derivative (indicated in each case) was added and the reaction was stirred at room temperature for 24 hours. Afterward, the reaction mixture was quenched with NH_4Cl, extracted with EtOAc, washed with brine, dried over $MgSO_4$ and concentrated under reduced pressure. The product was purified by column chromatography using cyclohexane: ethyl acetate and triethylamine as eluent mixture (indicated in each case).

SM1. (*E*)-2-(2-Tosylvinyl)aniline

This starting material was employed as precursor to form derivatives from **1a** to **1q**, **1v** and **1w**.

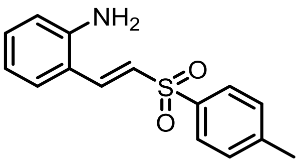

Synthesized following general procedure of HWE using 2-aminobenzaldehyde (3.35 mmol, 406 mg) as starting material, phosphonate (4.02 mmol, 1.23 g), *n*BuLi (4.69 mmol, 1.9 mL) as reagents and THF (35 mL) as solvent. The product was purified by column chromatography (cyclohexane: ethyl acetate 10:1 to 2:1 and 1% of triethylamine) to afford (*E*)-2-(2-tosylvinyl)aniline (**SM1**) as a yellow solid in quantitative yield (900 mg, 98%).

^{1}H-NMR (300 MHz, $CDCl_3$) δ (ppm) 7.84-7.74 (m, 3H), 7.37-7.31 (m, 2H), 7.26 (dd, *J* = 8.0, 1.4 Hz, 2H), 7.18 (ddd, *J* = 8.5, 7.4, 1.5, 1H), 6.82-6.67 (m, 3H), 2.43 (s, 3H). (*See spectrum*)

^{13}C-NMR (75 MHz, $CDCl_3$) δ (ppm) 146.1,144.4, 138.7, 138.1, 132.3, 130.1, 130.0, 128.8, 127.9, 127.8, 127.2, 119.3, 117.9, 117.3, 21.7. (*See spectrum*)

APCI-QTOF-MS (m/z): [M + H]$^+$ = calculated: 274.0901, found: 274.0889.

m.p. = 123-126 ºC.

Elemental Analysis: C = 65.61%, H = 5.75%, N = 5.15%, S = 11.70%.

SM2. (*E*)-5-Bromo-2-(2-tosylvinyl)aniline

This starting material was used as a precursor of **1t** and derivatives.

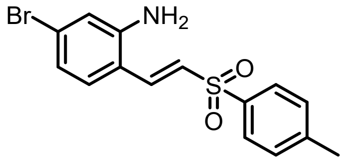

Synthesized following general procedure of HWE using 5-bromo-2-aminobenzaldehyde (5.0 mmol, 1 g) as starting material, phosphonate (6.0 mmol, 1.84 g), *n*BuLi (7.0 mmol, 2.8 mL) as reagents and THF (50 mL) as solvent. The product was purified by column chromatography (cyclohexane: ethyl acetate 7:1 to 2:1 and 1% of triethylamine) to afford (*E*)-5-bromo-2-(2-tosylvinyl)aniline (**SM2**) as a yellow solid (1.3 g, 73%).

^{1}H-NMR (300 MHz, $CDCl_3$) δ (ppm) 7.85-7.77 (m, 2H), 7.68 (d, *J* = 15.2 Hz, 1H), 7.38-7.30 (m, 2H), 7.10 (d, *J* = 8.3 Hz, 1H), 6.90-6.83 (m, 2H), 6.76 (d, *J* = 15.2 Hz, 1H), 2.44 (s, 3H). (*See spectrum*)

^{13}C-NMR (75 MHz, $CDCl_3$) δ (ppm) 147.0, 144.6, 137.8, 136.5, 130.2, 129.9, 127.8, 127.7, 126.3, 122.3, 119.8, 116.8, 21.8. (*See spectrum*)

APCI-MS (m/z): $[M + H]^+$ = calculated: 351.9986, found: 351.9998.

m.p. = 177-180 °C

Elemental Analysis: C = 51.42%, H = 3.98%, N = 4.10%, S = 9.3%.

SM3. (*E*)-6-(2-Tosylvinyl)benzo[*d*][1,3]dioxol-5-amine

This starting material was used as a precursor of **1u** and derivatives.

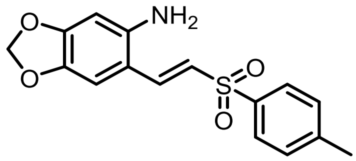

Synthesized following general procedure of HWE using 6-aminobenzo[*d*][1,3]dioxole-5-carbaldehyde (3.0 mmol, 500 mg) as starting material, phosphonate (3.64 mmol, 1.1 g), *n*BuLi (4.2 mmol, 1.7 mL) as reagents and THF (30 mL) as solvent. The product was purified by column chromatography (cyclohexane: ethyl acetate 10:1 to 2:1 and 1% of triethylamine) to afford **(***E*)-6-(2-tosylvinyl)benzo[*d*][1,3]dioxol-5-amine (**SM3**) as a yellow solid (901 mg, 94%).

^{1}H-NMR (300 MHz, $CDCl_3$) δ (ppm) 7.80 (d, *J* = 8.3 Hz, 2H), 7.69 (d, *J* = 15.0 Hz, 1H), 7.39-7.31 (m, 2H), 6.71 (s, 1H), 6.54 (d, *J* = 15.0 Hz, 1H), 6.24 (s, 1H), 5.88 (s, 2H), 3.89 (br s, 2H), 2.43 (s, 3H). (*See spectrum*)

^{13}C-NMR (75 MHz, $CDCl_3$) δ (ppm) 144.1, 141.7, 138.6, 136.9, 130.1, 127.6, 123.7, 110.1, 106.2, 101.5, 98.5, 21.7. (*See spectrum*)

APCI-MS (m/z): $[M + H]^+$ = calculated: 318.0779, found: 318.0793.

m.p. = 175-178 °C

Elemental Analysis: C = 60.13%, H = 4.90%, N = 4.52%, S = 9.9%.

SM4. (*Z*)-4-Methoxy-1-nitro-2-(2-tosylvinyl)benzene + (*E*)- 4-methoxy-1-nitro-2-(2-tosylvinyl)benzene

This starting material was used as a precursor of **1r** and derivatives.

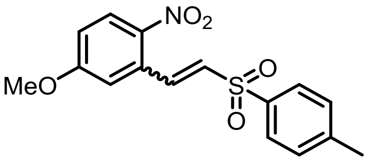

Synthesized following general procedure of HWE using 5-methoxy-2-nitrobenzaldehyde (5.6 mmol, 1g) as starting material, phosphonate (6.72 mmol, 2.2 g), *n*BuLi (7.84 mmol, 3.2 mL) as reagents and THF (56 mL) as solvent. The product was purified by column chromatography (cyclohexane: ethyl acetate 10:1 to 2:1) to afford 4-methoxy-1-nitro-2-(2-tosylvinyl)benzene (**SM4**) as an inseparable mixture of the two alkenes in a 72:28 (*Z*:*E*) ratio (600 mg, 40%). The spectrum was described assuming that both alkenes constitute just one molecule with 15H.

^{1}H-NMR (300 MHz, $CDCl_3$) δ (ppm) 8.21-8.11 (m, 0.49H), 8.04 (dd, *J* = 9.2, 1.3 Hz, 0.71H), 7.90-7.83 (m, 0.5H), 7.50-7.41 (m, 2.16H), 7.39-7.32 (m, 0.52H), 7.18-7.10 (m, 1.47H), 7.01-6.87 (m, 1.97H), 6.71 (d, *J* = 15.2 Hz, 0.74H), 6.63 (d, *J* = 11.5 Hz, 2.17H), 3.92 (s, 2.17H), 3.88 (s, 0.74H), 2.42 (s, 0.74H), 2.36 (s, 2.17H). (*See spectrum*)

^{13}C-NMR (75 MHz, $CDCl_3$) δ (ppm) 163.4, 145.0, 144.8, 140.1, 139.3, 137.3, 132.1, 132.0, 130.2, 129.7, 128.2, 128.0, 127.7, 127.1, 117.0, 115.6, 115.4, 114.7, 56.4, 21.8, 21.7. (*See spectrum*)

ESI-MS (m/z): $[M + Na]^+$ = calculated: 356.0569, found: 356.0558.

m.p. = 115-118 ^{o}C.

Elemental Analysis: C = 57.70%, H = 5.15%, N = 4.27%, S = 9.11%.

SM5. (*Z*)-4-Chloro-1-nitro-2-(2-tosylvinyl)benzene + (*E*)- 4-chloro-1-nitro-2-(2-tosylvinyl)benzene

This starting material has been used as a precursor of **1s** and derivatives.

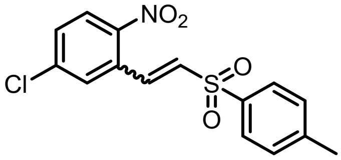

Synthesized following general procedure of HWE using 5-chloro-2-nitrobenzaldehyde (5.4 mmol, 1g) as starting material, phosphonate (6.48 mmol, 2.0 g), *n*BuLi (7.56 mmol, 3.1 mL) as reagents and THF (54 mL) as solvent. The product was purified by column chromatography (cyclohexane: ethyl acetate 10:1 to 2:1) to get 4-chloro-1-nitro-2-(2-tosylvinyl)benzene (**SM5**) as an inseparable mixture of the two alkenes in a 70:30 (*E*:*Z*) ratio (1.1 g, 61%). The spectrum was described assuming that both alkenes constitute just one molecule with 12H.

^{1}H-NMR (300 MHz, $CDCl_3$) δ (ppm) 8.12-8.08 (m, 0.66H), 8.08-8.03 (m, 1H), 7.89-7.83 (m, 1.4H), 7.55-7.48 (m, 2.15H), 7.48-7.41 (m, 0.63H), 7.41-7.34 (m, 1.56H), 7.25-7.19 (m, 0.57H), 6.78 (d, *J* = 15.2 Hz, 0.7H), 6.67 (d, *J* = 11.5 Hz, 0.3H), 2.44 (s, 0.7H), 2.40 (s, 0.3H). (*See spectrum*)

^{13}C-NMR (75 MHz, $CDCl_3$) δ (ppm) 146.1, 145.2, 145.0, 144.5, 140.6, 140.1, 137.3, 137.2, 137.2, 136.6, 133.8, 132.9, 132.0, 131.1, 131.2, 130.9, 130.3, 129.9, 129.8, 129.6, 128.2, 127.7, 126.8, 126.0, 21.8, 21.7. (*See spectrum*)

ESI-MS (m/z): [M + Na]⁺ = calculated: 360.0074, found: 360.0060.

m.p. = 125-130 °C.

Elemental Analysis: C = 53.09%, H = 4.12%, N = 4.26%, S = 9.47%.

2.2.-Reduction of nitro derivatives

According to the availability of starting materials, in some cases nitro derivatives were employed, which implies reduction to form the corresponding aniline. This step was performed either before or after HWE reaction.

General procedure of nitro reduction

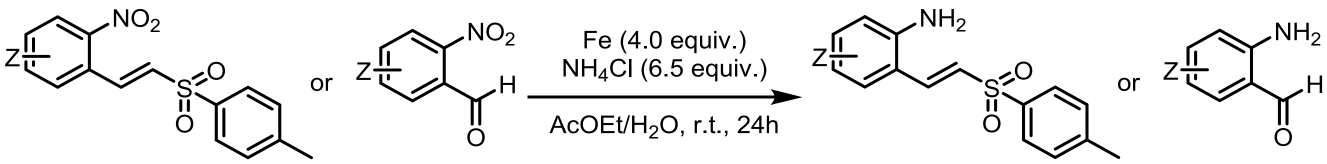

Synthesized following the procedure described in literature.[2] In a round-bottom flask, the corresponding starting material was added along with iron powder, ammonium chloride and the mixture of solvents (0.1 M for AcOEt and 0.16 M for H_2O). The mixture was stirred at room temperature for 24 hours. Afterward, the crude was filtered through celite and washed with EtOAc. Then it was extracted with EtOAc, washed with brine, dried over $MgSO_4$ and concentrated under reduced pressure.

SM6. 6-Aminobenzo[d][1,3]dioxole-5-carbaldehyde

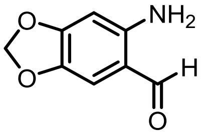

Synthesized following general procedure of nitro reduction using 6-nitrobenzo[*d*][1,3]dioxole-5-carbaldehyde (5.12 mmol, 1 g) as starting material, iron powder (20.48 mmol, 1.1 g), ammonium chloride (33.28 mmol, 1.8 g) as reagents and EtOAc (51.2 mL) and H_2O (32 mL) as solvents. The product (**SM6**) was obtained as a yellow solid without purification (785 mg, 93%). Spectroscopic data were in accordance with the literature.[3]

^{1}H-NMR (300 MHz, $CDCl_3$) δ(ppm) 9.6 (s, 1H), 6.82 (s, 1H), 6.30 (br s, 2H), 6.14 (s, 1H), 5.92 (s, 2H). (*See Spectrum*)

SM7. (*Z*)-4-Methoxy-2-(2-tosylvinyl)aniline + (*E*)-4-methoxy-2-(2-tosylvinyl)aniline

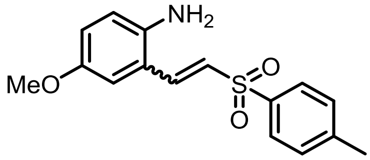

Synthesized following general procedure of nitro reduction using 4-methoxy-1-nitro-2-(2-tosylvinyl)benzene (**SM4**) (0.9 mmol, 300 mg) as starting material, iron powder (3.6 mmol, 201 mg), ammonium chloride (5.85 mmol, 313 mg) as reagents and EtOAc (9 mL) and H_2O (5.6 mL) as solvents. The product (**SM7**) was obtained as a yellow syrup without purification (265 mg, 97%) as an inseparable mixture of two alkenes with a 2:1 ratio. The spectrum was described assuming that both alkenes constitute just one molecule with 15H.

^{1}H-NMR (300 MHz, $CDCl_3$) δ (ppm) 7.83-7.73 (m, 1.91H), 7.62-7.55 (m, 0.58H), 7.33-7.27 (m, 1.28H), 7.20-7.13 (m, 0.58H), 7.03-6.96 (m, 0.35H), 6.87-6.49 (m, 3.8H), 3.70 (s, 1H), 3.68 (s, 2H), 2.40 (s, 2H), 2.35 (s, 1H). (*See spectrum*)

^{13}C-NMR (75 MHz, $CDCl_3$) δ (ppm) 153.1, 152.1, 144.3, 140.5, 138.1, 138.0, 137.9, 137.6, 133.4, 130.0, 129.5, 127.6, 126.9, 119.9, 119.0, 118.4, 117.7, 117.3, 114.8, 111.7, 60.4, 55.2, 21.6, 14.2. (*See spectrum*)

ESI-MS (m/z): $[M + H]^+$ = calculated: 304.1007, found: 304.1001.

SM8. (*E*)-4-Chloro-2-(2-tosylvinyl)aniline

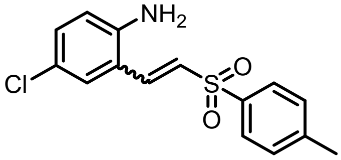

Synthesized following general procedure of nitro reduction using 4-chloro-1-nitro-2-(2-tosylvinyl)benzene (**SM5**) (0.9 mmol, 300 mg) as starting material, iron powder (3.6 mmol, 201 mg), ammonium chloride (5.85 mmol, 313 mg) as reagents and EtOAc (9 mL) and H_2O (5.6 mL) as solvents. The product (**SM8**) was obtained as a yellow syrup without purification (242 mg, 87%).

^{1}H-NMR (300 MHz, $CDCl_3$) δ (ppm) 7.82-7.76 (m, 2H), 7.70 (d, *J* = 15.2 Hz, 1H), 7.27 (d, *J* = 8.2 Hz, 2H), 7.13 (d, *J* = 2.5 Hz, 1H), 7.00 (dd, *J* = 8.7, 2.4 Hz, 1H), 6.74 (d, *J* = 15.2 Hz, 1H), 6.58 (d, *J* = 8.6 Hz, 1H), 4.26 (br s, 2H), 2.37 (s, 3H). (*See spectrum*)

^{13}C-NMR (75 MHz, $CDCl_3$) δ (ppm) 145.0, 144.5, 137.5, 136.3, 130.0, 127.6, 127.4, 118.5, 118.4, 21.6. (*See spectrum*)

ESI-MS (m/z): $[M + Na]^+$ = calculated: 308.0512, found: 308.0503.

3.- Synthesis of imines

General procedure

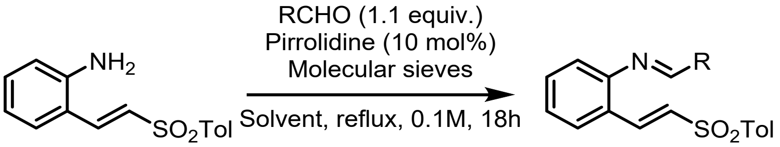

Imines were synthesized according to a literature-reported procedure,[4] starting from the corresponding aldehyde and amine. In a screw-capped vial, all solid reagents were added: the amine (0.9 mmol), activated molecular sieves (1 g per mmol of amine), and the corresponding aldehyde (1 mmol), if it was solid. The vial was evacuated and backfilled with argon three times. The solid mixture was then dissolved in 9 mL of dichloromethane. Subsequently, pyrrolidine (0.01 mmol) and the aldehyde (1 mmol), if it was liquid, were added. The reaction mixture was stirred under reflux for 18 hours.

Upon completion, the crude reaction mixture was filtered through a pad of celite, washed with dichloromethane, and concentrated under reduced pressure. If necessary, the crude product was purified by flash chromatography using a mixture of cyclohexane, ethyl acetate, and triethylamine, as specified for each individual case.

(*E*)-1-Phenyl-*N*-(3-((*E*)-2-tosylvinyl)phenyl)methanimine (1a)

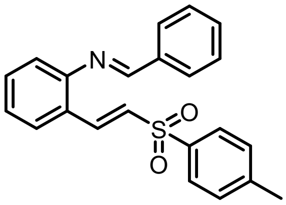

Synthesized according to general procedure starting from (*E*)-2-(2-tosylvinyl)aniline and benzaldehyde to afford **1a** as a yellow syrup in quantitative yield (315 mg, 97%).

^{1}H-NMR (300 MHz, $CDCl_3$) δ (ppm) 8.37 (s, 1H), 8.06 (d, *J* = 15.5 Hz, 1H), 7.92-7.86 (m, 2H), 7.83-7.79 (m, 2H), 7.54-7.49 (m, 3H), 7.42 (td, *J* = 7.7, 1.5 Hz, 1H), 7.33-7.28 (m, 2H), 7.22 (dd, *J* = 7.7, 1.2 Hz, 2H), 7.02 (dd, *J* = 7.9, 1.2 Hz, 1H), 6.96 (d, *J* = 15.5 Hz, 1H), 2.42 (s, 3H). (*See spectrum*)

^{13}C-NMR (75 MHz, $CDCl_3$) δ (ppm) 161.1, 151.5, 144.2, 138.9, 138.1, 135.9, 132.0, 129.9, 129.4, 129.2, 129.0, 128.9, 127.8, 126.6, 126.2, 118.9, 27.0, 21.6. (*See spectrum*)

APCI-QTOF-MS (m/z): $[M + H]^+$ = calculated:362.1234, found: 362.1206.

(*E*)-1-*o*-Tolyl-*N*-(2((*E*)-2-tosylvinyl)phenyl)methanimine (1b)

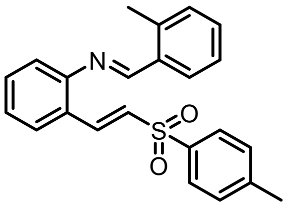

Synthesized according to general procedure starting from (*E*)-2-(2-tosylvinyl)aniline and *o*-tolylbenzaldehyde. The product was purified by column chromatography (cyclohexane: ethyl acetate 8:1 to 7:1 and 1% of triethylamine) to afford **1b** as a yellow syrup (263 mg, 70%).

^{1}H-NMR (300 MHz, $CDCl_3$) δ (ppm) 8.60 (s, 1H), 8.02 (d, *J* = 15.5 Hz, 1H), 7.95 (dd, *J* = 7.7, 1.5 Hz, 1H), 7.78-7.72 (m, 2H), 7.46 (dd, *J* = 7.7, 1.5 Hz, 1H), 7.41-7.31 (m, 2H), 7.30-7.15 (m, 4H), 6.95 (dd, *J* = 7.7, 1.5 Hz, 1H), 6.91 (d, *J* = 15.5 Hz, 1H), 2.56 (s, 3H), 2.37 (s, 3H). (See *spectrum*)

^{13}C-NMR (75 MHz, $CDCl_3$) δ (ppm) 160.1, 152.3, 144.2, 139.2, 139.1, 138.1, 133.8, 132.1, 131.7, 131.4, 130.0, 129.4, 128.9, 128.8, 127.9, 126.6, 126.2, 119.1, 21.7, 19.8. (See spectrum)

APCI-QTOF-MS (m/z): [M + H]$^+$ = calculated:376.1371, found: 376.1370.

(*E*)-1-(4-Fluorophenyl)-*N*-(2-((*E*)-2-tosylvinyl)phenyl)methanimine (1c)

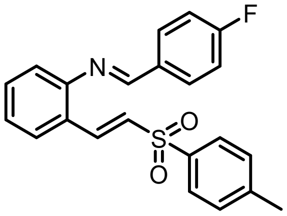

Synthesized according to general procedure starting from (*E*)-2-(2-tosylvinyl)aniline and *p*-fluorobenzaldehyde to afford **1c** as a yellow solid in quantitative yield (339 mg, 99%).

^{1}H-NMR (300 MHz, $CDCl_3$) δ (ppm) 8.31 (s, 1H), 8.04 (d, *J* = 15.5 Hz, 1H), 7.90-7.82 (m, 1H), 7.77 (d, *J* = 8.0 Hz, 2H), 7.48 (d, *J* = 7.6 Hz, 1H), 7.39 (t, *J* = 7.6 Hz, 1H), 7.28 (d, *J* = 8.0 Hz, 2H), 7.23-7.09 (m, 4H), 6.98 (d, *J* = 7.6 Hz, 1H), 6.90 (d, *J* = 15.5 Hz, 1H), 2.40 (s, 3H). (*See spectrum*)

^{13}C-NMR (75 MHz, $CDCl_3$) δ (ppm) 159.5, 151.2, 144.2, 138.7, 138.0, 132.0, 131.2, 131.1, 130.0, 129.3, 128.8, 127.7, 126.6, 126.3, 53.5, 21.6. (See *spectrum*)

^{19}F-NMR (282 MHz, $CDCl_3$) δ (ppm) -106.9 (*See spectrum*)

APCI-QTOF-MS (m/z): [M + H]$^+$ = calculated:380.1120, found: 380.1113.

m.p. = 125-128 ^{o}C.

(*E*)-1-(4-Chlorophenyl)-*N*-(2-((*E*)-2-tosylvinyl)phenyl)methanimine (1d)

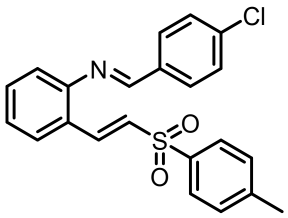

Synthesized according to general procedure starting from (*E*)-2-(2-tosylvinyl)aniline and *p*-chlorobenzaldehyde to afford **1d** as a yellow solid in quantitative yield (351 mg, 98%).

^{1}H-NMR (300 MHz, $CDCl_3$) δ (ppm) 8.31 (s, 1H), 8.03 (d, *J* = 15.5 Hz, 1H), 7.86-7-70 (m, 4H), 7.55-7.15 (m, 8H), 6.99 (d, *J* = 7.5 Hz, 1H), 6.89 (d, *J* = 15.4 Hz, 1H), 2.40 (s, 3H). (<u>*See spectrum*</u>)

^{13}C-NMR (75 MHz, $CDCl_3$) δ (ppm) 159.4, 151.0, 144.5, 138.6, 138.0, 134.3, 132.0, 130.9, 129.9, 129.3, 128.8, 127.7, 126.6, 126.4, 118.8, 21.6, 19.1. (<u>*See spectrum*</u>)

APCI-QTOF-MS (m/z): $[M + H]^+$ = calculated:396.0825, found: 396.0810.

m.p. = 147-150 °C.

(*E*)-1-(4-Bromophenyl)-*N*-(2-((*E*)-2-tosylvinyl)phenyl)methanimine (1e)

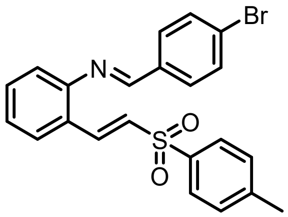

Synthesized according to general procedure starting from (*E*)-2-(2-tosylvinyl)aniline and *p*-bromobenzaldehyde. The product was purified by column chromatography (cyclohexane: ethyl acetate 10:1, 7:1, 2:1 and 1% of triethylamine) to afford **1e** as a yellow syrup (317 mg, 80%).

^{1}H-NMR (300 MHz, $CDCl_3$) δ (ppm) 8.33 (s, 1H), 8.05 (d, *J* = 15.6 Hz, 1H), 7.84-7.78 (m ,2H), 7.78-7.73 (m, 2H), 7.65-7.61 (m, 2H), 7.51 (dd, *J* = 7.8, 1.5 Hz, 1H), 7.42 (td, *J* = 7.7, 1.5 Hz, 1H), 7.34-7.29 (m, 2H), 7.25 (td, *J* = 7.6, 1.3 Hz, 2H), 7.01 (dd, *J* = 7.9, 1.2 Hz, 1H), 6.92 (d, *J* = 15.6 Hz, 1H), 2.43 (s, 3H). (<u>*See spectrum*</u>)

^{13}C-NMR (75 MHz, $CDCl_3$) δ (ppm) 159.8, 151.1, 144.3, 138.7, 138.1, 134.8, 132.4, 132.0, 130.5, 129.5, 128.9, 127.9, 126.8, 126.7, 126.6, 118.8, 21.7. (<u>*See spectrum*</u>)

APCI-QTOF-MS (m/z): $[M + H]^+$ = calculated:440.0320, found: 440.0316.

Elemental Analysis: C = 59.93%, H = 4.44%, N = 3.12%, S = 7.26%.

(*E*)-1-*p*-Tolyl-*N*-(2((*E*)-2-tosylvinyl)phenyl)methanimine (1f)

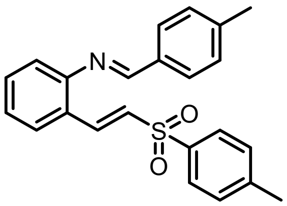

Synthesized according to general procedure starting from (*E*)-2-(2-Tosylvinyl)aniline and *p*-tolylbenzaldehyde to afford **1f** as a yellow solid in quantitative yield (331 mg, 98%).

^{1}H-NMR (300 MHz, $CDCl_3$) δ (ppm) 8.30 (s, 1H), 8.03 (d, *J* = 15.5 Hz, 1H), 7.82-7.70 (m, 4H), 7.47 (d, *J* = 7.7 Hz, 1H), 7.38 (t, *J* = 7.7 Hz, 1H), 7.29-7.23 (m, 4H), 7.19 (t, *J* = 7.7 Hz, 1H), 7.02-6.90 (m, 2H), 2.43 (s, 3H), 2.39 (s, 3H). (*See spectrum*)

^{13}C-NMR (75 MHz, $CDCl_3$) δ (ppm) 161.0, 151.9, 144.2, 142.7, 139.1, 138.2, 133.4, 132.0, 120.0, 129.8, 129.4, 129.3, 129.1, 127.9, 126.6, 126.1, 119.1, 21.8, 21.7. (*See spectrum*)

APCI-QTOF-MS (m/z): [M + H]$^+$ = calculated:376.1371, found: 376.1368.

m.p. = 115-118 ^{o}C.

(*E*)-1-(4-Methoxyphenyl)-*N*-(2-((*E*)-2-tosylvinyl)phenyl)methanimine (1g)

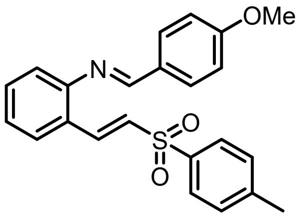

Synthesized according to general procedure starting from (*E*)-2-(2-Tosylvinyl)aniline and *p*-methoxybenzaldehyde to afford **1g** as a yellow solid in quantitative yield (345 mg, 97%).

^{1}H-NMR (300 MHz, $CDCl_3$) δ (ppm) 8.26 (s, 1H), 8.05 (d, *J* = 15.4 Hz, 1H), 7.84-7.74 (m, 4H), 7.46 (d, *J* = 7.6 Hz, 1H), 7.37 (t, *J* = 7.6 Hz, 1H), 7.28 (d, *J* = 7.6 Hz, 1H), 7.18 (t, *J* = 7.6 Hz, 1H), 7.00-6.90 (m, 4H), 3.87 (s, 3H), 2.39 (s, 3H). (*See spectrum*)

^{13}C-NMR (75 MHz, $CDCl_3$) δ (ppm) 162.7, 160.2, 151.8, 144.1, 139.0, 128.1, 121.9, 130.9, 129.9, 129.1, 128.9, 127.7, 126.4, 125.8, 119.0, 114.4, 55.5, 53.5, 52.7, 23.6, 21.6, 19.1. (*See spectrum*)

APCI-QTOF-MS (m/z): [M + H]$^+$ = calculated:392.1330, found: 392.1317.

m.p. = 118-121 ^{o}C.

(*E*)-1-(4-Benzyloxy)phenyl)-*N*-(2-((*E*)-2-tosylvinyl)phenyl)methanimine (1h)

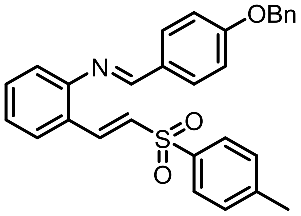

Synthesized according to general procedure starting from (*E*)-2-(2-Tosylvinyl)aniline and *p*-(benzyloxy)benzaldehyde to afford **1h** as a yellow solid in quantitative yield (410 mg, 97%).

^{1}H-NMR (300 MHz, $CDCl_3$) δ (ppm) 8.29 (s, 1H), 8.05 (d, *J* = 15.5 Hz, 1H), 7.86-7.77 (m, 4H), 7.52-7.34 (m, 7H), 7.33-7.27 (m, 2H), 7.20 (td, *J* = 7.6, 1.2, 1H), 7.10-7.04 (m, 4H), 7.02-7.91 (m, 2H), 5.17 (s, 2H), 2.42 (s, 3H). (*See spectrum*)

^{13}C-NMR (75 MHz, $CDCl_3$) δ (ppm) 162.0, 160.2, 151.9, 144.2, 139.2, 128.2, 136.5, 132.0, 131.0, 130.0, 129.3, 129.1, 128.8, 128.3, 127.8, 127.6, 126.6, 125.9, 119.1, 115.4, 70.3, 27.0, 21.7 (*See spectrum*)

APCI-QTOF-MS (m/z): [M + H]$^+$ = calculated:468.1633, found: 468.1622.

m.p. = 144-146 ^{o}C.

(*E*)-1-(4-Methylthio)phenyl)-*N*-(2-((*E*)-2-tosylvinyl)phenyl)methanimine (1i)

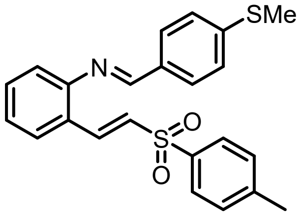

Synthesized according to general procedure starting from (*E*)-2-(2-Tosylvinyl)aniline and 4-(methylthio)benzaldehyde to afford **1i** as a yellow syrup in quantitative yield (342 mg, 93%).

^{1}H-NMR (300 MHz, $CDCl_3$) δ (ppm) 8.31 (s, 1H), 8.06 (d, *J* = 15.5 Hz, 1H), 7.85-7.73 (m, 4H), 7.50 (dd, *J* = 7.8, 1.5 Hz, 1H), 7.41 (td, *J* = 7.7, 1.5 Hz, 1H), 7.35-7.29 (m, 3H), 7.22 (td, *J* = 7.6, 1.3, 1H), 7.01 (dd, *J* = 7.9, 1.2 Hz, 1H), 6.95 (d, *J* = 15.5 Hz, 1H), 2.56 (s, 3H), 2.42 (s, 3H). (*See spectrum*)

^{13}C-NMR (75 MHz, $CDCl_3$) δ (ppm) 160.3, 151.7, 144.3, 144.2, 139.1, 138.2, 132.6, 132.0, 130.0, 129.5, 129.1, 127.9, 126.7, 126.2, 125.9, 119.0, 21.7, 15.2. (*See spectrum*)

APCI-QTOF-MS (m/z): [M + H]$^+$ = calculated:408.1092, found: 408.1090.

Elemental Analysis: C = 67.31%, H = 5.31%, N = 3.45%, S = 15.28%.

N,N-Dimethyl-4-((E)-((2-((E)-2-tosylvinyl)phenyl)imino)methyl)aniline (1j)

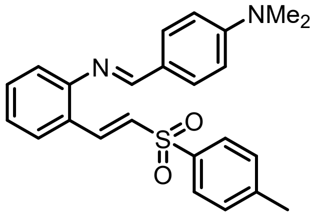

Synthesized according to general procedure starting from (*E*)-2-(2-Tosylvinyl)aniline and 4-(dimethylamino)benzaldehyde to afford **1j** as a yellow solid in quantitative yield (351 mg, 96%).

^{1}H-NMR (300 MHz, $CDCl_3$) δ (ppm) 8.22 (s, 1H), 8.06 (d, *J* = 15.5 Hz, 1H), 7.85-7.78 (m, 2H), 7.78-7.71 (m, 2H), 7.47 (dd, *J* = 7.7, 1.5 Hz, 1H), 7.38 (td, *J* = 7.7, 1.5 Hz, 1H), 7.32-7.26 (m, 2H), 7.16 (td, *J* = 7.6, 1.2 Hz, 1H), 7.06-6.97 (m, 2H), 6.77-6.69 (m, 2H), 3.09 (s, 6H), 2.42 (s, 3H). (*See spectrum*)

^{13}C-NMR (75 MHz, $CDCl_3$) δ (ppm) 160.6, 152.9, 152.7, 144.0, 139.6, 138.4, 131.9, 131.0, 129.9, 129.2, 129.0, 127.8, 126.4, 125.2, 124.2, 119.3, 111.7, 40.3, 21.7. (*See spectrum*)

APCI-QTOF-MS (m/z): [M + H]$^+$ = calculated:405.1636, found: 405.1632.

m.p. = 119-123 ^{o}C.

(E)-N-(2-((E)-2-tosylvinyl)phenyl)-1-(4-trifluoromethyl)phenyl)methanimine (1k)

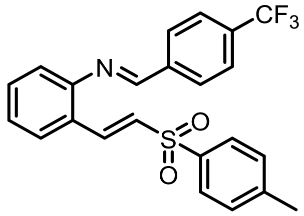

Synthesized according to general procedure starting from (*E*)-2-(2-Tosylvinyl)aniline and 4-(trifluoromethyl)benzaldehyde to afford **1k** as a yellow solid in quantitative yield (377 mg, 98%).

^{1}H-NMR (300 MHz, $CDCl_3$) δ (ppm) 8.36 (s, 1H), 8.00 (d, *J* = 15.5 Hz, 1H), 7.96-7.89 (m, 2H), 7.77-7.71 (m, 2H), 7.68 (d, *J* = 8.1 Hz, 2H), 7.47 (dd, *J* = 7.8, 1.5 Hz, 1H), 7.37 (td, *J* = 7.6, 1.5 Hz, 1H), 7.28-7.19 (m, 4H), 6.97 (dd, *J* = 8.0, 1.3 Hz, 1H), 6.85 (d, *J* = 15.5 Hz, 1H), 2.36 (s, 3H). (*See spectrum*)

^{13}C-NMR (75 MHz, $CDCl_3$) δ (ppm) 159.5, 150.7, 144.4, 138.9, 138.5, 138.0, 132.1, 130.0, 129.6, 129.4, 128.8, 127.8, 126.9, 125.9, 118.1, 21.7. (*See spectrum*)

^{19}F-NMR (282 MHz, $CDCl_3$) δ -62.9 (*See spectrum*)

APCI-QTOF-MS (m/z): [M + H]$^+$ = calculated:430.1088, found: 430.1075.

m.p. = 147-149 ^{o}C.

Methyl 4-((*E*)-((2-((*E*)-2-tosylvinyl)phenyl)imino)methyl)benzoate (1l)

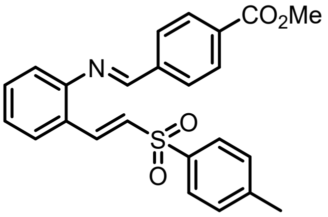

Synthesized according to general procedure starting from (*E*)-2-(2-Tosylvinyl)aniline and methyl 4-formylbenzoate. The product was purified by column chromatography (cyclohexane: ethyl acetate 10:1, 7:1, 2:1 and 1% of triethylamine) to afford **1l** as a yellow solid (261 mg, 69%).

^{1}H-NMR (300 MHz, $CDCl_3$) δ (ppm) 8.43 (s, 1H), 8.15 (d, *J* = 8.3 Hz, 2H), 8.06 (d, *J* = 15.5 Hz, 1H), 7.94 (d, *J* = 8.4 Hz, 2H), 8.81 (d, *J* = 8.3 Hz, 2H), 7.53 (dd, *J* = 7.7, 1.5 Hz, 1H), 7.44 (td, *J* = 7.6, 1.4 Hz, 1H), 7.34-7.27 (m, 3H), 7.06-7.01 (m, 1H), 6.93 (d, *J* = 15.5 Hz, 1H), 3.98 (s, 3H), 2.43 (s, 3H). (*See spectrum*)

^{13}C-NMR (75 MHz, $CDCl_3$) δ (ppm) 166.5, 159.9, 150.9, 144.3, 139.5, 138.5, 138.0, 132.9, 132.0, 130.2, 130.0, 129.7, 120.0, 128.9, 127.8, 126.9, 126.8, 118.7, 52.5, 21.7. (*See spectrum*)

APCI-QTOF-MS (m/z): $[M + H]^+$ = calculated:420.1269, found: 420.1263.

m.p. = 144-146 °C.

Elemental Analysis: C = 68.21%, H = 5.43%, N = 3.31%, S = 7.59%.

(*E*)-1-(4-Nitrophenyl)-*N*-(2-((*E*)-2-tosylvinyl)phenyl)methanimine (1m)

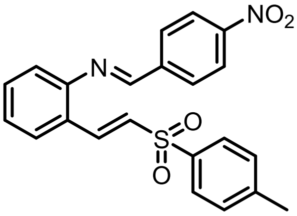

Synthesized according to general procedure starting from (*E*)-2-(2-Tosylvinyl)aniline and *p*-nitrobenzaldehyde to afford **1m** as an orange solid in quantitative yield (352 mg, 96%). It was contaminated with <5% of aldehyde, but it was used without purification.

^{1}H-NMR (300 MHz, $CDCl_3$) δ (ppm) 8.49 (s, 1H), 8.36-8.30 (m, 2H), 8.15-8.02 (m, 3H), 7.85-7.77 (m, 2H), 7.55 (dd, *J* = 7.8, 1.5 Hz, 1H), 7.45 (td, *J* = 7.7, 1.5 Hz, 1H), 7.37-7.27 (m, 3H), 7.07 (dd, *J* = 7.9, 1.2 Hz, 1H), 6.90 (d, *J* = 15.5 Hz, 1H), 2.44 (s, 3H). (*See spectrum*)

^{13}C-NMR (75 MHz, $CDCl_3$) δ (ppm) 158.5, 150.3, 149.7, 144.5, 141.1, 138.3, 137.9, 132.1, 130.1, 129.8, 129.7, 128.8, 127.9, 127.4, 127.2, 124.3, 118.6, 21.7. (*See spectrum*)

APCI-QTOF-MS (m/z): $[M + H]^+$ = calculated:407.1065,;found: 407.1054.

m.p. = 169-172 °C.

Elemental Analysis: C = 64.41%; H = 4.63%, N = 6.93%, S = 7.71%.

(*E*)-1-(Pyridin-4-yl)-*N*-(2-((*E*)-2-tosylvinyl)phenyl)methanimine (1n)

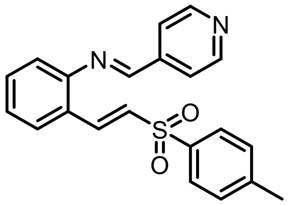

Synthesized according to general procedure starting from (*E*)-2-(2-Tosylvinyl)aniline and isonicotinaldehyde. The product was purified by column chromatography (cyclohexane: ethyl acetate 10:1, 7:1, 2:1 and 1% of triethylamine) to afford **1n** as a brown solid (261 mg, 69%).

^{1}H-NMR (300 MHz, $CDCl_3$) δ (ppm) 8.83-8.74 (m, 2H), 8.39 (s, 1H), 8.07 (d, *J* = 15.5 Hz, 1H), 7.85-7.76 (m, 2H), 7.76-7.67 (m, 2H), 7.53 (dd, *J* = 7.7, 1.5 Hz, 1H), 7.44 (td, *J* = 7.6, 1.4 Hz, 1H), 7.36-7.23 (m, 3H), 7.05 (dd, *J* = 7.9, 1.2 Hz, 1H), 6.91 (d, *J* = 15.5 Hz, 1H), 2.42 (s, 3H). (*See spectrum*)

^{13}C-NMR (75 MHz, $CDCl_3$) δ (ppm) 159.0, 150.8, 150.2, 144.4, 142.3, 138.2, 137.8, 132.0, 130.0, 129.8, 127.8, 127.3, 127.0, 122.4, 118.6, 21.7. (*See spectrum*)

APCI-QTOF-MS (m/z): $[M + H]^+$ = calculated:363.1167, found: 363.1157.

m.p. = 133-135 °C.

Elemental Analysis: C = 69.13%, H = 5.25%, N = 7.97%, S = 8.67%.

(*E*)-1-(Naphthalen-1-yl)-*N*-(2-((*E*)-2-tosylvinyl)phenyl)methanimine (1o)

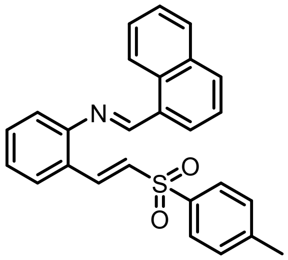

Synthesized according to general procedure starting from (*E*)-2-(2-tosylvinyl)aniline and 1-naphthaldehyde. The product was purified by column chromatography (cyclohexane: ethyl acetate 10:1, 7:1, 2:1 and 1% of triethylamine) to afford **1o** as a yellow syrup (189 mg, 53%).

^{1}H-NMR (300 MHz, $CDCl_3$) δ (ppm) 8.53 (s, 1H), 8.21-8.05 (m, 3H), 7.93 (dt, *J* = 9.0, 3.4 Hz, 3H), 7.83-7.70 (m, 2H), 7.64-7.50 (m, 3H), 7.45 (td, *J* = 7.7, 1.5 Hz, 1H), 7.29 (d, *J* = 3.5 Hz, 1H), 7.08 (dd, *J* = 7.9, 1.2 Hz, 2H), 7.01 (d, *J* = 15.5 Hz, 1H), 2.39 (s, 3H). (*See spectrum*)

^{13}C-NMR (75 MHz, $CDCl_3$) δ (ppm) 161.1, 151.5, 144.2, 138.9, 138.1, 135.3, 133.6, 133.1, 132.1, 132.0, 129.9, 129.5, 129.0, 128.9, 128.1, 128.0, 127.7, 126.8, 126.7, 126.3, 123.8, 11.9, 21.6. (*See spectrum*)

APCI-QTOF-MS (m/z): $[M + H]^+$ = calculated:412.1371, found: 412.1365.

(*E*)-1-(Furan-2-yl)-*N*-(2-((*E*)-2-tosylvinyl)phenyl)methanimine (1p)

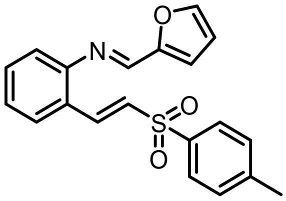

Synthesized according to general procedure starting from (*E*)-2-(2-Tosylvinyl)aniline and furan-2-carbaldehyde. The product was purified by column chromatography (cyclohexane: ethyl acetate 10:1, 7:1, 2:1 and 1% of triethylamine) to afford **1p** as a brown syrup (249 mg, 79%).

^{1}H-NMR (300 MHz, $CDCl_3$) δ (ppm) 8.17 (s, 1H), 8.03 (d, *J* = 15.5 Hz, 1H), 7.85-7.80 (m, 2H), 7.66-7.64 (m, 1H), 7.50 (dd, *J* = 7.7, 1.5, 1H), 7.40 (td, *J* = 7.7, 1.5 Hz, 1H), 7.31 (d, *J* = 8.1 Hz, 2H), 7.22 (td, *J* = 8.0, 3.0 Hz, 1H), 7.05-6.96 (m, 3H), 6.60 (dd, *J* = 3.5, 1.8 Hz, 1H), 2.42 (s, 3H). (*See spectrum*)

^{13}C-NMR (75 MHz, $CDCl_3$) δ (ppm) 152.0, 151.4, 148.7, 146.2, 144.1, 138.8, 138.0, 131.9, 129.9, 128.9, 127.7, 126.4, 126.2, 118.8, 116.7, 112.5, 26.9, 21.6. (*See spectrum*)

APCI-QTOF-MS (m/z): $[M + H]^+$ = calculated:352.1007, found: 352.1005.

Elemental Analysis: C = 68.35%, H = 5.18%, N = 4.16%, S = 9.43%.

(*E*)-1-(Thiophen-2-yl)-*N*-(2-((*E*)-2-tosylvinyl)phenyl)methanimine (1q)

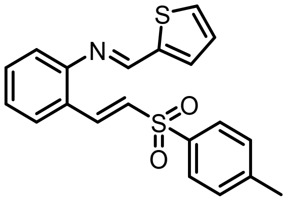

Synthesized according to general procedure starting from (*E*)-2-(2-Tosylvinyl)aniline and thiophene-2-carbaldehyde. The product was purified by column chromatography (cyclohexane: ethyl acetate 10:1, 7:1, 2:1 and 1% of triethylamine) to afford **1o** as a yellow syrup (275 mg, 83%).

^{1}H-NMR (300 MHz, $CDCl_3$) δ (ppm) 8.47 (s, 1H), 7.97 (d, *J* = 15.5 Hz, 1H), 7.86-7.80 (m, 2H), 7.57 (td, *J* = 5.0, 1.1 Hz, 1H), 7.53-7.44 (m, 2H), 7.40 (td, *J* = 7.6, 1.5 Hz, 1H), 7.34-7.28 (m, 2H), 7.22 (td, *J* = 7.6, 1.3 Hz, 1H), 7.16 (dd, *J* = 5.0, 3.7 Hz, 1H), 7.10-6.99 (m, 2H), 2.42 (s, 3H). (*See spectrum*)

^{13}C-NMR (75 MHz, $CDCl_3$) δ (ppm) 153.6, 150.9, 144.1, 142.7, 139.1, 138.1, 132.9, 131.9, 131.5, 130.0, 129.9, 129.7, 128.1, 127.8, 126.7, 126.3, 118.9, 21.7. (*See spectrum*)

APCI-QTOF-MS (m/z): $[M + H]^+$ = calculated:368.0779, found: 368.0527.

Elemental Analysis: C = 64.94%, H = 4.84%, N = 3.82%, S = 16.94%.

(E)-*N*-(4-Methoxy-2-((*E*)-2-tosylvinyl)phenyl)-1-phenylmethanimine (1r)

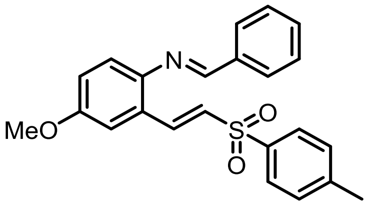

Synthesized according to general procedure starting from (*E*)-4-methoxy-2-(2-tosylvinyl)aniline and benzaldehyde to afford **1r** as a mixture of isomers E and Z (285 mg, 81%).

^{1}H-NMR (300 MHz, $CDCl_3$) δ (ppm) 8.38 (s, 1H), 8.19-8.08 (m, 1H), 7.90-7.76 (m, 4H), 7.31 (d, *J* = 7.9 Hz, 2H), 7.08-6.90 (m, 5H), 3.88 (s, 1H), 3.81 (s, 3H), 2.41 (s, 3H), 2.34 (s, 3H). (*See spectrum*)

^{13}C-NMR (75 MHz, $CDCl_3$) δ (ppm) 159.5, 158.2, 144.8, 144.3, 139.0, 138.1, 136.2, 131.8, 130.0, 129.6, 129.1, 128.2, 128.0, 119.7, 118.3, 112.9, 55.8, 21.7. (*See spectrum*)

ESI-MS (m/z): [M + H]$^+$ = calculated: 392.1320, found: 392.1398

m.p. = 133-137 ^{o}C.

(*E*)-*N*-(4-Chloro-2-((*E*)-2-tosylvinyl)phenyl)-1-phenylmethanimine (1s)

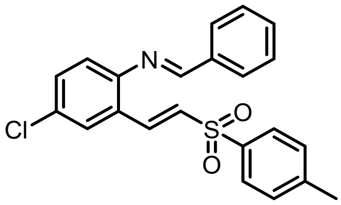

Synthesized according to general procedure starting from (*E*)-4-chloro-2-(2-tosylvinyl)aniline and benzaldehyde to afford **1s** as a yellow solid (242 mg, 68%).

^{1}H-NMR (300 MHz, $CDCl_3$) δ (ppm) 8.35 (s, 1H), 8.01 (d, *J* = 15.6 Hz, 1H), 7.87 (dd, *J* = 8.0, 1.7 Hz, 2H), 7.80 (d, *J* = 8.3 Hz, 2H), 7.57-7.43 (m, 4H), 7.39-7.28 (m, 3H), 7.00-6.91 (m, 2H), 2.42 (s, 3H). (*See spectrum*)

^{13}C-NMR (75 MHz, $CDCl_3$) δ (ppm) 161.5, 149.8, 144.5, 137.7, 137.3, 135.6, 132.3, 131.8, 131.7, 130.6, 130.0, 129.3, 129.1, 128.3, 128.2, 127.9, 120.2, 21.7. (*See spectrum*)

APCI-QTOF-MS (m/z): [M + H]$^+$ = calculated: 396.0804, found: 396.0824.

m.p. = 142-145 ^{o}C.

(*E*)-*N*-(5-Bromo-2-((*E*)-tosylvinyl)phenyl)-1-phenylmethanimine (1t)

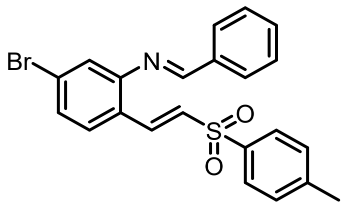

Synthesized according to general procedure starting from (*E*)-5-bromo-2-(2-tosylvinyl)aniline and benzaldehyde to afford **1t** as a yellow solid (377 mg, 95%). This imine was not purified and the product was used as a crude after work-up. NMR presented below corresponds to the crude reaction.

^{1}H-NMR (300 MHz, $CDCl_3$) δ (ppm) 8.36 (s, 1H), 7.96 (d, *J* = 15.5 Hz, 1H), 7.91-7.84 (m, 2H), 7.82-7.76 (m, 2H), 7.61-7.46 (m, 3H), 7.36 (d, *J* = 1.1 Hz, 2H), 7.33-7.26 (m, 2H), 7.18 (t, *J* = 1.2 Hz, 1H), 6.95 (d, *J* = 15.5 Hz, 1H), 2.42 (s, 3H). (*See spectrum*)

^{13}C-NMR (75 MHz, $CDCl_3$) δ (ppm) 162.0, 152.4, 144.7, 137.8, 137.7, 135.5, 132.4, 130.1, 130.0, 129.9, 129.4, 129.2, 129.1, 127.9, 125.7, 125.7, 122.1, 21.7. (*See spectrum*)

APCI-QTOF-MS (m/z): $[M + H]^+$ = calculated: 440.0299, found: 440.0300.

m.p. = 170-172 ^{o}C.

(*E*)-1-Phenyl-*N*-(6-((*E*)-2-tosylvinyl)benzo[*d*][1,3]dioxol-5-yl)methanimine (1u)

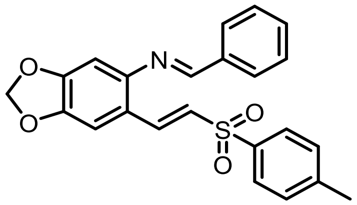

Synthesized according to general procedure starting from (*E*)-6-(2-tosylvinyl)benzo[*d*][1,3]dioxol-5-amine and benzaldehyde to afford **1u** as a yellow syrup (172 mg, 47%).

^{1}H-NMR (300 MHz, $CDCl_3$) δ (ppm) 8.37 (s, 1H), 8.20 (d, *J* = 15.6 Hz, 1H), 7.89 (dd, *J* = 7.6, 2.1 Hz, 2H), 7.84-7.78 (m, 2H), 7.54-7.47 (m, 3H), 7.33-7.28 (m, 2H), 6.95 (s, 1H), 6.72 (d, *J* = 15.6 Hz, 1H), 6.65 (s, 2H), 2.42 (s, 3H). (*See spectrum*)

^{13}C-NMR (75 MHz, $CDCl_3$) δ (ppm) 159.7, 151.0, 146.8, 146.7, 144.1, 138.5, 138.4, 136.0, 131.9, 129.2, 129.0, 127.8, 126.4, 121.2, 106.4, 102.1, 99.2, 21.7. (*See spectrum*)

APCI-QTOF-MS (m/z): $[M + H]^+$ = calculated: 406.1092, found: 406.1106

(*E*)-1-Cyclopropyl-*N*-(2-((*E*)-2-tosylvinyl)phenyl)methanimine (1v)

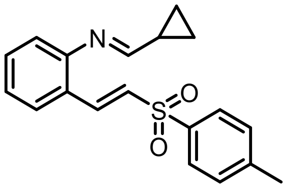

Synthesized according to general procedure starting from (*E*)-2-(2-Tosylvinyl)aniline and cyclopropanecarbaldehyde to afford **1v** as a yellow syrup in quantitative yield (287 mg, 98%). Due to the volatility of the product, the ^{1}H-NMR spectrum displays signals corresponding to cyclohexane

^{1}H-NMR (300 MHz, $CDCl_3$) δ (ppm) 8.47 (s, 1H), 7.97 (d, *J* = 15.5 Hz, 1H), 7.86-7.80 (m, 2H), 7.57 (td, *J* = 5.0, 1.1 Hz, 1H), 7.53-7.44 (m, 2H), 7.40 (td, *J* = 7.6, 1.5 Hz, 1H), 7.34-7.28 (m, 2H), 7.22 (td, *J* = 7.6, 1.3 Hz, 1H), 7.16 (dd, *J* = 5.0, 3.7 Hz, 1H), 7.10-6.99 (m, 2H), 2.42 (s, 3H). (*See spectrum*)

^{13}C-NMR (75 MHz, $CDCl_3$) δ (ppm) 153.6, 150.9, 144.1, 142.7, 139.1, 138.1, 132.9, 131.9, 131.5, 130.0, 129.9, 129.7, 128.1, 127.8, 126.7, 126.3, 118.9, 21.7. (*See spectrum*)

APCI-QTOF-MS (m/z): $[M + H]^+$ = calculated: 326.1194, found: 326.1205.

(*E*)-2,2-Dimethyl-*N*-(2-((*E*)-2-tosylvinyl)phenyl)propan-1-imine (1w)

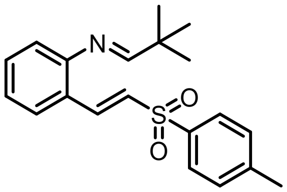

Synthesized according to general procedure starting from (*E*)-2-(2-Tosylvinyl)aniline and pivalaldehyde to afford **1w** as a yellow syrup (114 mg, 37%).

^{1}H-NMR (300 MHz, $CDCl_3$) δ (ppm) 7.88-7.76 (mm, 3H), 7.62 (s, 1H), 7.42 (dd, *J* = 7.8, 1.5 Hz, 1H), 7.38-7.28 (m, 3H), 7.15 (td, *J* = 7.6, 1.3 Hz, 1H), 7.00 (d, *J* = 15.5 Hz, 1H), 6.78 (dd, *J* = 7.9, 1.3 Hz, 1H), 2.42 (s, 3H), 1.18 (s, 9H). (*See spectrum*)

^{13}C-NMR (75 MHz, $CDCl_3$) δ (ppm) 174.5, 152.2, 144.3, 138.9, 138.2, 131.9, 130.0, 129.3, 128.8, 127.9, 125.6, 125.5, 119.6, 37.5, 26.8, 21.7. (*See spectrum*)

APCI-QTOF-MS (m/z): $[M + H]^+$ = calculated: 342.1507, found: 342.1520.

4.- Optimization of the quinoline formation

Additional information about the experiments of the optimization of the 2-phenylquinoline (**2a**) formation are collected in Table S1.

Table S1: Reaction of imines with diboron reagents

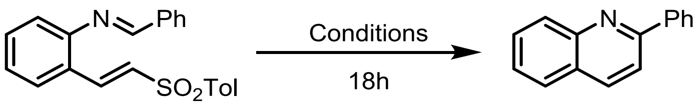

1a **2a**

Entry	Solvent	B-B (Equiv.)	Pyridine	Yield (%)[a]
**1**	Mesytilene (reflux)	B_2nep_2 (1.0)	4,4'-bipy (30 mol%)	69
**2**	Toluene (reflux)	B_2nep_2 (2.0)	4,4'-bipy (30 mol%)	51
**3**	Toluene (70 ºC)	B_2nep_2 (1.0)	4,4'-bipy (30 mol%)	60
**4**	Toluene	B_2nep_2 (30 mol%)	4,4'-bipy (30 mol%)	22
**5**	Toluene	B_2pin_2 (30 mol%)	4,4'-bipy (30 mol%)	19
**7**	Toluene	B_2nep_2 (1.0)	4,4'-bipy (20 mol%)	0
**7**	Toluene	B_2nep_2 (1.0)	4,4'-bipy (10 mol%)	0
**8**	Toluene	B_2nep_2 (1.0)	4,4'-bipy (5 mol%)	0

All experiments were performed following conditions indicated in the manuscript (Table 1) and the procedure described in pg.2. [a] Yields determined adding CH_2Br_2 as external standard after the indicated reaction time.

Another reaction was performed using B_2nep_2 and $PPh_3Pyr^+ OTf^-$.

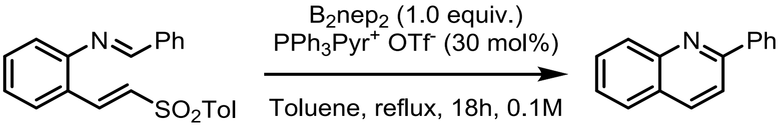

1a **2a** (50%)

4.1.- Formation of quinoline with imine in situ formation

To circumvent the purification and isolation of the imine intermediate, an experiment was conducted in which all reagents were combined in a single vial to evaluate the feasibility of a one-pot reaction, wherein imine formation would occur in situ prior to quinoline synthesis.

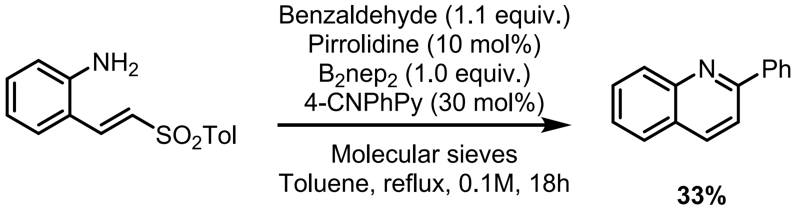

Experimental procedure: They were prepared combining general procedure of imine formation (*see pg.10*) and general procedure of quinoline formation (*see pg. 23*). In a screw-capped vial, all the solids were added, amine (0.11 mmol, 30.1 mg), activated molecular sieves (110 mg), B_2nep_2 (0.11 mmol, 24.9 mg) and the pyridine (0.033 mmol, 6 mg). The vial was evacuated and backfilled with argon three times. Then, the mixture

of solids was dissolved in 1.1 mL of dry toluene. Then the pyrrolidine (0.011 mmol, 1 µL) and the benzaldehyde (0.121 mmol, 12.3 µL) were added. The solution was stirred for 18 hours at reflux. Afterward the toluene was removed under reduced pressure and CH_2Br_2 was added as external standard to check the yield by ^{1}H-NMR. The yield obtained was lower than performing both steps separately.

5.- Formation of quinoline via cyclization reaction using diboron reagents and imines

General procedure of quinoline formation

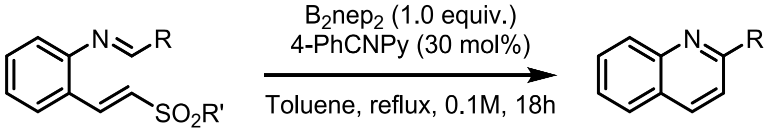

In a screw-capped vial, B_2nep_2 (0.11 mmol, 24.9 mg) was added along with the pyridine (0.033 mmol, 6 mg) and the corresponding imine (0.11 mmol). The vial was evacuated and backfilled with argon three times. The mixture of solids was dissolved in 1.1 mL of dry toluene and the solution was stirred for 18 hours at reflux. Afterward, the solvent was removed under under reduced pressure and the mixture was purified by flash chromatography using cyclohexane/ethyl acetate mixture indicated for each case.

2-Phenylquinoline (2a)

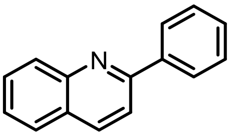

Synthesized according to general procedure of quinoline formation starting from (*E*)-1-phenyl-*N*-(3-((*E*)-2-tosylvinyl)phenyl)methanimine (**1a**). The product was purified by column chromatography (cyclohexane: ethyl acetate 100: 0, 20:1 and 10:1) to afford **2a** (108.5 mg, 82%). Spectroscopic data were in accordance with the literature.[5] **^{1}H-NMR (300 MHz, $CDCl_3$)** δ (ppm) 8.23-8.17 (m, 4H), 7.88 (d, *J* = 8.6 Hz, 1H), 7.83 (d, *J* = 8.1 Hz, 1H), 7.76-7.72 (m, 1H), 7.56-7.46 (m, 4H). (*See spectrum*)

2-(*o*-Tolyl)quinoline (2b)

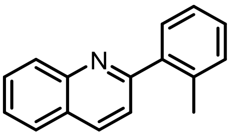

Synthesized according to general procedure of quinoline formation starting from (*E*)-1-*o*-tolyl-*N*-(2((*E*)-2-tosylvinyl)phenyl)methanimine **(1b)**. The product was purified by column chromatography (cyclohexane: ethyl acetate 100: 0, 20:1 and 10:1) to afford **2b** (14.2 mg, 59%). Spectroscopic data were in accordance with the literature.[6] **^{1}H-NMR (300 MHz, $CDCl_3$)** δ (ppm) 8.15 (t, *J* = 9.5 Hz, 2H), 7.80 (d, *J* = 8.0 Hz, 1H), 7.72−7.68 (m, 1H), 7.53−7.48 (m, 3H), 7.31−7.29 (m, 3H), 2.40 (s, 3H). (*See spectrum*)

2-(4-Fluorophenyl)quinoline (2c)

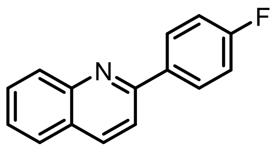

Synthesized according to general procedure of quinoline formation starting from (*E*)-1-(4-fluorophenyl)-*N*-(2-((*E*)-2-tosylvinyl)phenyl)methanimine **(1c)**. The product was purified by column chromatography (cyclohexane: ethyl acetate 100: 0, 20:1 and 10:1) to afford **2c** (20.9 mg, 85%). Spectroscopic data were in accordance with the literature.[7] **^{1}H-NMR (300 MHz, CDCl$_3$)** δ (ppm) 8.27-8.10 (m, 4H), 7.88-7.78 (m, 2H), 7.77-7.70 (m, 1H), 7.58-7.50 (m, 1H), 7.24-7.15 (m, 2H). (*See spectrum*)

2-(4-Chlorophenyl)quinoline (2d)

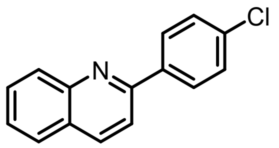

Synthesized according to general procedure of quinoline formation starting from (*E*)-1-(4-chlorophenyl)-*N*-(2-((*E*)-2-tosylvinyl)phenyl)methanimine **(1d)**. The product was purified by column chromatography (cyclohexane: ethyl acetate 100: 0, 20:1 and 10:1) to afford **2d** (18.5 mg, 85%). Spectroscopic data were in accordance with the literature.[8] **^{1}H-NMR (300 MHz, CDCl$_3$)** δ (ppm) 8.19 (d, *J* =8.8 Hz, 1H), 8.15 (d, *J* = 8.7 Hz, 1H), 8.11-8.09 (m, 2H), 7.81 (d, *J* = 8.7 Hz, 2H), 7.74-7.71 (m, 1H), 7.54-7.52 (m, 1H), 7.50-7.47 (m, 2H). (*See spectrum*)

2-(4-Bromophenyl)quinoline (2e)

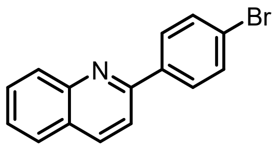

Synthesized according to general procedure of quinoline formation starting from (*E*)-1-(4-bromophenyl)-*N*-(2-((*E*)-2-tosylvinyl)phenyl)methanimine **(1e)**. The product was purified by column chromatography (cyclohexane: ethyl acetate 100: 0, 20:1 and 10:1) to afford **2e** (21.9 mg, 70%). Spectroscopic data were in accordance with the literature.[9] **^{1}H-NMR (300 MHz, CDCl$_3$)** δ (ppm) 8.23 (d, *J* = 8.7 Hz, 1H), 8.16 (d, *J* = 8.4 Hz, 1H), 8.09-8.03 (m, 2H), 7.87-7.81 (m, 2H), 7.74 (ddd, *J* = 8.5, 6.9, 1.5 Hz, 1H), 7.69-7.63 (m, 2H), 7.54 (ddd, *J* = 8.2, 6.9 1.2 Hz, 1H). (*See spectrum*)

2-(*p*-Tolyl)quinoline (2f)

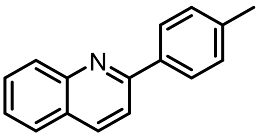

Synthesized according to general procedure of quinoline formation starting from (*E*)-1-*p*-tolyl-*N*-(2((*E*)-2-tosylvinyl)phenyl)methanimine **(1f)**. The product was purified by column chromatography (cyclohexane: ethyl acetate 100: 0, 20:1 and 10:1) to afford **2f** (15.0 mg, 62%). Spectroscopic data were in accordance with the literature.[7] **^{1}H-NMR (300 MHz, $CDCl_3$)** δ (ppm) 8.10 (dd, *J* = 16.4, 8.6 Hz, 2H), 8.00 (d, *J* = 8.2 Hz, 2H), 7.78 (d, *J* = 8.6 Hz, 1H), 7.74 (d, *J* = 7.2 Hz, 1H), 7.64 (t, *J* = 7.0 Hz, 1H), 7.43 (t, *J* = 7.5 Hz, 1H), 7.26 (d, *J* = 8.1 Hz, 2H), 2.36 (s, 3H). (*See spectrum*)

2-(4-Methoxyphenyl)quinoline (2g)

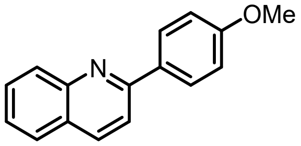

Synthesized according to general procedure of quinoline formation starting from (*E*)-1-(4-methoxyphenyl)-*N*-(2-((*E*)-2-tosylvinyl)phenyl)methanimine **(1g)**. The product was purified by column chromatography (cyclohexane: ethyl acetate 100: 0, 20:1 and 10:1) to afford **2g** (20.2 mg, 78%). Spectroscopic data were in accordance with the literature.[6] **^{1}H-NMR (300 MHz, $CDCl_3$)** δ (ppm) 8.17-8.12 (m, 4H), 7.83-7.78 (m, 2H), 7.72-7.68 (m, 1H), 7.50-7.46 (m, 1H), 7.04 (d, *J* = 8.7 Hz, 2H), 3.87 (s, 3H). (*See spectrum*)

2-(4-(Benzyloxy)phenyl)quinoline (2h)

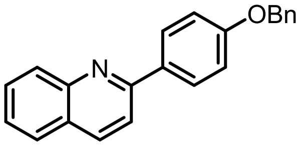

Synthesized according to general procedure of quinoline formation starting from (*E*)-1-(4-benzyloxy)phenyl)-*N*-(2-((*E*)-2-tosylvinyl)phenyl)methanimine **(1h)**. The product was purified by column chromatography (cyclohexane: ethyl acetate 100: 0, 20:1 and 10:1) to afford **2h** (19.2 mg, 56%). Spectroscopic data were in accordance with the literature.[10] **^{1}H-NMR (300 MHz, $CDCl_3$)** δ (ppm) 8.18 (d, *J* = 8.6 Hz, 1H), 8.15-8.13 (m, 3H), 7.83 (d, *J* = 6.0 Hz, 1H), 7.81 (d, *J* = 6.0 Hz, 1H), 7.72-7.70 (m, 1H), 7.51-7.47 (m, 3H), 7.41 (t, *J* = 6.0 Hz, 2H), 7.36-7.33 (m, 1H), 7.14-7.12 (m, 2H), 5.16 (s, 2H). (*See spectrum*)

2-(4-(Methylthio)phenyl)quinoline (2i)

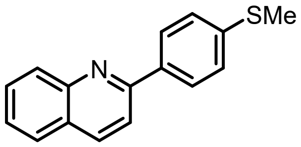

Synthesized according to general procedure of quinoline formation starting from (*E*)-1-(4-methylthio)phenyl)-*N*-(2-((*E*)-2-tosylvinyl)phenyl)methanimine **(1i)**. The product was purified by column chromatography (cyclohexane: ethyl acetate 100: 0, 20:1 and 10:1) to afford **2i** (13.8 mg, 55%). Spectroscopic data were in accordance with the literature.[11] **^{1}H-NMR (300 MHz, $CDCl_3$)** δ (ppm) 8.23-8.14 (m, 2H), 8.11 (d, *J* = 8.1 Hz, 2H), 7.85-7.80 (m, 2H), 7.72 (t, *J* = 7.6 Hz, 1H), 7.51 (t, *J* = 7.5 Hz, 1H), 7.38 (d, *J* = 8.1 Hz, 2H), 2.54 (s, 3H). (*See spectrum*)

***N*, *N*-Dimethyl-4-(quinoline-2-yl)aniline (2j)**

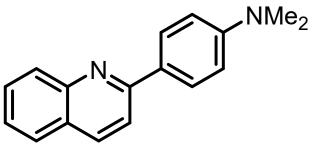

Synthesized according to general procedure of quinoline formation starting from *N,N*-dimethyl-4-((*E*)-((2-((*E*)-2-tosylvinyl)phenyl)imino)methyl)aniline **(1j)**. The product was purified by column chromatography (cyclohexane: ethyl acetate 100: 0, 20:1 and 10:1) to afford **2j** (23.2 mg, 85%). Spectroscopic data were in accordance with the literature.[12] **^{1}H-NMR (300 MHz, $CDCl_3$)** δ (ppm) 8.17-8.06 (m, 4H), 7.84 (d, *J* = 8.6 Hz, 1H), 7.80-7.74 (m, 1H), 7.72-7.65 (m, 1H), 7.50-7.42 (m, 1H), 6.89-6.79 (m, 2H), 3.06 (s, 6H). (*See spectrum*)

2-(4-(Trifluoromethyl)phenyl)quinoline (2k)

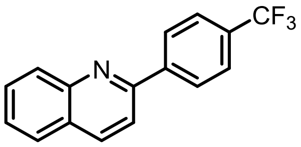

Synthesized according to general procedure of quinoline formation starting from (*E*)-*N*-(2-((*E*)-2-tosylvinyl)phenyl)-1-(4-trifluoromethyl)phenyl)methanimine **(1k)**. The product was purified by column chromatography (cyclohexane: ethyl acetate 100: 0, 20:1 and 10:1) to afford **2k** (17.9 mg, 60%). Spectroscopic data were in accordance with the literature.[5] **^{1}H-NMR (300 MHz, $CDCl_3$)** δ (ppm) 8.28-8.24 (m, 3H), 8.18 (d, *J* = 8.5 Hz, 1H), 7.87 (d, *J* = 8.5 Hz, 1H), 7.84 (d, *J* = 8.0 Hz, 1H), 7.77 (d, *J* = 8.0 Hz, 2H), 7.76-7.71 (m, 1H), 7.59-7.52 (m, 1H). (*See spectrum*)

Methyl-4-(quinoline-2-yl)benzoate (2l)

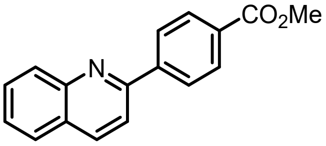

Synthesized according to general procedure of quinoline formation starting from methyl 4-((*E*)-((2-((*E*)-2-tosylvinyl)phenyl)imino)methyl)benzoate **(1l)**. The product was purified by column chromatography (cyclohexane: ethyl acetate 100: 0, 20:1 and 10:1) to afford **2l** (14.5 mg, 50%). Spectroscopic data were in accordance with the literature.[9] **^{1}H-NMR (300 MHz, $CDCl_3$)** δ (ppm) 8.26-8.18 (m, 6H), 7.90 (d, *J* = 8.5 Hz, 1H), 7.84 (d, *J* = 8.1 Hz, 1H), 7.75 (t, *J* = 7.6 Hz, 1H), 7.56 (t, *J* = 7.5 Hz, 1H), 3.96 (s, 3H). (*See spectrum*)

2-(Napthalen-1-yl)quinoline (2o)

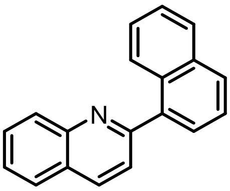

Synthesized according to general procedure of quinoline formation starting from methyl (*E*)-1-(naphthalen-1-yl)-*N*-(2-((*E*)-2-tosylvinyl)phenyl)methanimine **(1o)**. The product was purified by column chromatography (cyclohexane: ethyl acetate 100: 0, 20:1 and 10:1) to afford **2o** (5.6 mg, 20%). Spectroscopic data were in accordance with the literature.[9] **^{1}H-NMR (300 MHz, $CDCl_3$)** δ (ppm) 8.51 (s, 1H), 8.27 (d, *J* = 8.6 Hz, 1H), 8.13 (t, *J* = 7.9 Hz, 2H), 7.93-7.86 (m, 3H), 7.81-7.76 (m, 1H), 7.73 (d, *J* = 8.1 Hz, 1H), 7.65 (t, *J* = 7.9 Hz, 1H), 7.45-7.41 (m, 3H). (*See spectrum*)

2-(Furan-2-yl)quinoline (2p)

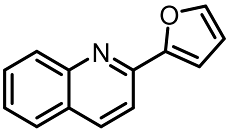

Synthesized according to general procedure of quinoline formation starting from methyl ((*E*)-1-(furan-2-yl)-*N*-(2-((*E*)-2-tosylvinyl)phenyl)methanimine **(1p)**. The product was purified by column chromatography (cyclohexane: ethyl acetate 100: 0, 20:1 and 10:1) to afford **2p** (11.8 mg, 55%). Spectroscopic data were in accordance with the literature.[7] **^{1}H-NMR (300 MHz, $CDCl_3$)** δ (ppm) 8.14 (t, *J* = 8.5 Hz, 1H), 7.83 (d, *J* = 8.3 Hz, 1H), 7.78 (dd, *J* = 8.0, 1.5 Hz, 1H), 7.70 (ddd, *J* = 8.5, 6.9, 1.5 Hz, 1H), 7.63 (dd, *J* = 1.8, 0.8 Hz, 1H), 7.53-7.44 (m, 1H), 7.22 (dd, *J* = 3.4, 0.9 Hz, 1H), 6.59 (dd, *J* = 3.4, 1.8 Hz, 1H). (*See spectrum*)

2-(Thiophen-2-yl)quinoline (2q)

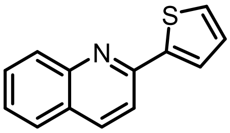

Synthesized according to general procedure of quinoline formation starting from methyl (*E*)-1-(thiophen-2-yl)-*N*-(2-((*E*)-2-tosylvinyl)phenyl)methanimine **(1q)**. The product was purified by column chromatography (cyclohexane: ethyl acetate 100: 0, 20:1 and 10:1) to afford **2q** (17.4 mg, 75%). Spectroscopic data were in accordance with the literature.[5] **^{1}H-NMR (300 MHz, $CDCl_3$)** δ (ppm) 8.12 (t, *J* = 9.0 Hz, 2H), 7.79 (t, *J* = 9.0 Hz, 2H), 7.77-7.65 (m, 2H), 7.53-7.44 (m, 2H), 7.16 (dd, *J* = 5.1, 3.7 Hz, 1H). (*See spectrum*)

6-Methoxy-2-phenylquinoline (2r)

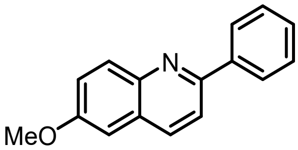

Synthesized according to general procedure of quinoline formation starting from methyl (E)-*N*-(4-methoxy-2-((*E*)-2-tosylvinyl)phenyl)-1-phenylmethanimine **(1r)**. The product was purified by column chromatography (cyclohexane: ethyl acetate 20:1, 15:1 and 10:1) to afford **2r** (26.7 mg, 87%). Spectroscopic data were in accordance with the literature.[13] **^{1}H-NMR (300 MHz, $CDCl_3$)** δ (ppm) 8.15-8.11 (m, 3H), 8.07 (d, *J* = 9.2 Hz, 1H), 7.84 (d, *J* = 8.8 Hz, 1H), 7.54-7.50 (m, 2H), 7.47-7.42 (m, 1H), 7.39 (dd, *J* = 9.2, 2.8 Hz, 1H), 7.10 (d, *J* = 2.8 Hz, 1H), 3.95 (s, 3H). (*See spectrum*)

6-Chloro-2-phenylquinoline (2s)

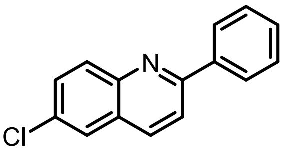

Synthesized according to general procedure of quinoline formation starting from methyl (E)-*N*-(4-chloro-2-((*E*)-2-tosylvinyl)phenyl)-1-phenylmethanimine **(1s)**. The product was purified by column chromatography (cyclohexane: ethyl acetate 100:0, 100:1 and 50:1) to get **2s** (18.2 mg, 69%). Spectroscopic data were in accordance with the literature.[7] **^{1}H-NMR (300 MHz, $CDCl_3$)** δ (ppm) 8.17-8.14 (m, 3H), 8.11 (d, *J* = 9.1 Hz, 1H), 7.91 (d, *J* = 8.2 Hz, 1H), 7.82 (d, *J* = 2.3 Hz, 1H), 7.67 (dd, *J* = 8.9, 2.5 Hz, 1H), 7.56-7.46 (m, 3H). (*See spectrum*)

7-Bromo-2-phenylquinoline (2t)

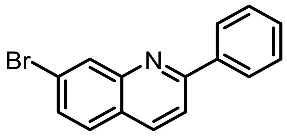

Synthesized according to general procedure of quinoline formation starting from methyl (E)-*N*-(5-bromo-2-((*E*)-2-tosylvinyl)phenyl)-1-phenylmethanimine **(1t)**. The product was purified by column chromatography (cyclohexane: ethyl acetate 100:0, 100:1 and 50:1) to get **2t** (21.9 mg, 70%). Spectroscopic data were in accordance with the literature.[14] **^{1}H-NMR (300 MHz, $CDCl_3$)** δ (ppm) 11.88 (s, 1H), 8.16 (d, *J* = 8.3 Hz, 1H), 7.69 (q, *J* = 2.2 Hz, 2H), 7.39-7.32 (m, 6H), 7.22 (td, *J* = 5.5, 3.3 Hz, 2H), 7.09.-7.06 (m, 2H). (*See spectrum*)

6-Phenyl-[1,3]dioxolo[4,5-*g*]quinoline (2u)

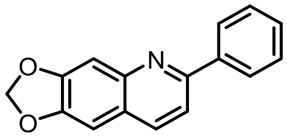

Synthesized according to general procedure of quinoline formation starting from methyl (*E*)-1-phenyl-*N*-(6-((*E*)-2-tosylvinyl)benzo[*d*][1,3]dioxol-5-yl)methanimine **(1u)**. The product was purified by column chromatography (cyclohexane: ethyl acetate 100: 0, 100:1 and 50:1) to afford **2u** (15.1 mg, 55%). Spectroscopic data were in accordance with the literature.[15] **^{1}H-NMR (300 MHz, $CDCl_3$)** δ (ppm) 8.16 (d, *J* = 7.6 Hz, 2H), 8.03 (d, *J* = 8.5 Hz, 1H), 7.75 (d, *J* = 8.5 Hz, 1H), 7.58-7.42 (m, 4H), 7.09 (s, 1H), 6.13 (s, 2H). (*See spectrum*)

6.- Mechanistic studies

6.1.- Autocatalysis (control experiment)

Substituted pyridines act as organocatalysts in this reaction, leading to quinoline derivatives as final products. Since both pyridines and quinolines belong to the class of aza-heteroaromatic compounds, we decided to evaluate whether quinoline itself could also catalyze the process. To this end, the reaction was performed using the product quinoline as the catalyst.

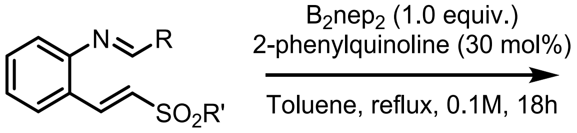

In a screw-capped vial, B_2nep_2 (0.11 mmol, 24.9 mg), 2-phenylquinoline (0.033 mmol, 6.8 mg), and the corresponding imine (0.11 mmol, x mg) were added. The vial was evacuated and backfilled with argon three times. The solids were dissolved in 1.1 mL of dry toluene, and the solution was stirred under reflux for 18 hours. After solvent removal under vacuum, the crude mixture was analyzed by ^{1}H NMR, showing no reaction. To confirm this, CH_2Br_2 (7.7 µL, 0.11 mmol) was added as an external standard. NMR analysis revealed that over 98% of the imine remained unreacted, and only the initially added 2-phenylquinoline was present (≈30% relative to CH_2Br_2), thus ruling out the possibility of autocatalysis.

6.2.- Identification of intermediates and by-products

As discussed in the main text, although slight differences in yield are observed, quinoline formation occurs using 4-CNPy (**P6**) with both B_2nep_2 (**B1**) and B_2pin_2 (**B2**), suggesting that the same reaction mechanism is likely followed in both cases. The proposed mechanism shown in Scheme 3 and Figure 1 is supported by the detection of three types of species: PinB–O–Bpin / NepB–O–Bnep, *p*-tolyl disulfide, and the protonated form of **IVa(5)** and/or **IVa(6)**. The evidence for the formation of these species is discussed below.

A. Detection of pinB-O-Bpin and nepB-O-Bnep (by-products)

PinB–O–Bpin was unambiguously identified by ^{11}B-NMR, displaying a characteristic signal at 22.4 ppm (Figure 1). Its detection by ^{1}H-NMR is hindered by signal overlap with the starting diboron reagent, highlighting the importance of ^{11}B-NMR for its identification. The formation of this boron-containing by-product is attributed to the deoxygenating ability of diboron reagents. As described in the manuscript, the reaction can proceed with substoichiometric amounts of diboron, however, one equivalent is employed to increase the yield. This justifies the use of one equivalent of B_2pin_2 in the reaction: part of the diboron reagent is consumed during the deoxygenation of sulfinic acid–derived intermediates, effectively being sacrificed and no longer available for the main catalytic cycle.

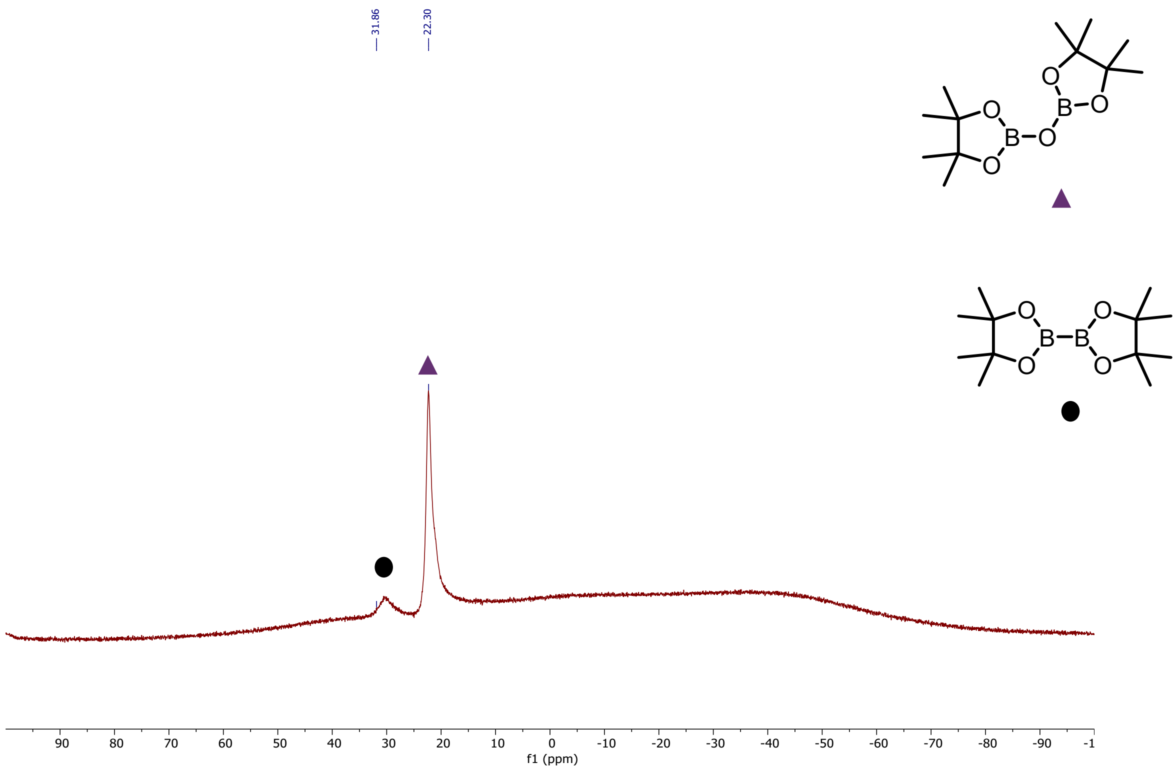

Figure 1. ^{11}B-NMR signal of PinB–O–Bpin at 22.4 ppm.

NepB–O–Bnep was identified by GC-MS analysis of the crude reaction mixture obtained using B_2nep_2 as the diboron source, 4-CNPhPy as the pyridine catalyst, and imine **1a**. The compound was detected at 3.3 min with a molecular ion peak at *m/z* 243.1576 corresponding to $[M+H]^+$ (Figure 2).

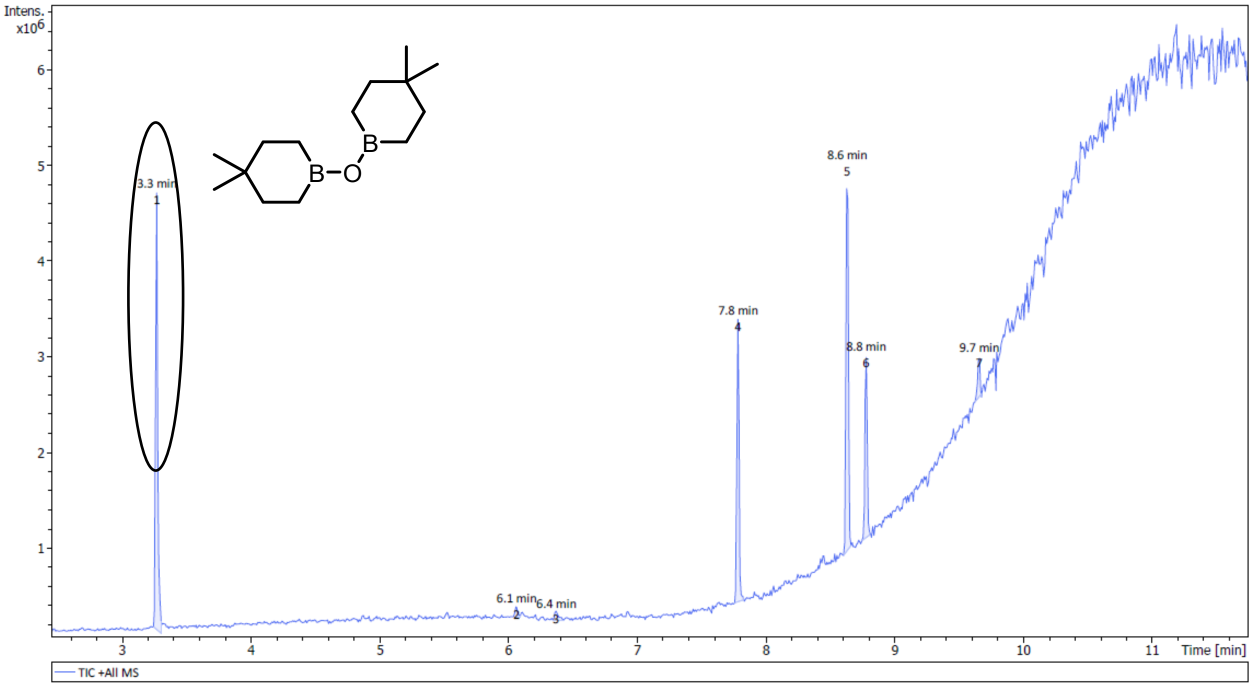

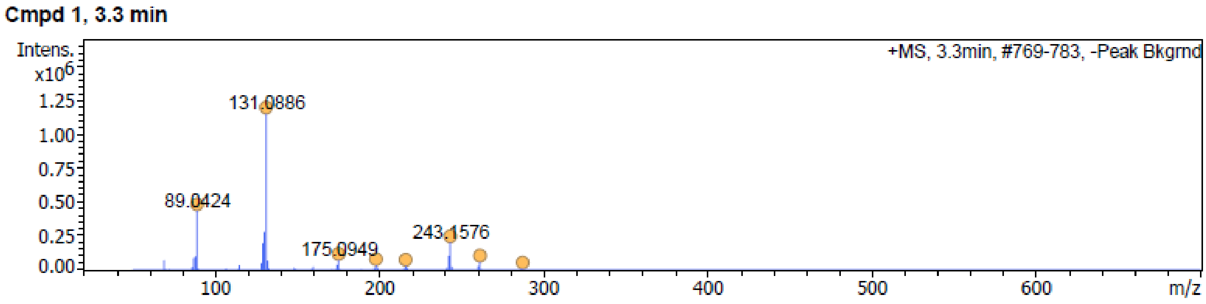

Meas. m/z	#	Ion Formula	m/z	err [ppm]	mSigma	#mSigma	Score	rdb (neutral)	e⁻ Conf	N-Rule
89.0424	1	C5H4BN	89.0431	8.9	21.5	1	98.40	5.5	odd	ok
89.0424	2	C4H9S	89.0419	-5.5	27.5	2	100.00	3.0	even	ok
131.0886	1	C5H12BO3	131.0874	-8.1	1.6	1	100.00	1.0	even	ok
131.0886	2	C7H15S	131.0889	2.6	28.2	2	63.87	3.0	even	ok
175.0949	1	C5H13B2O5	175.0944	-1.8	13.5	1	100.00	1.0	even	ok
175.0949	2	C4H7B2N7	175.0944	-2.1	15.7	2	74.82	6.5	odd	ok
175.0949	3	C6H17B2S2	175.0952	3.5	41.5	3	53.77	4.0	even	ok
175.0949	4	C5H14BN3OS	175.0945	-1.5	136.9	4	0.64	3.5	odd	ok
198.0883	1	C8H16B2S2	198.0874	-2.7	146.1	1	0.10	6.5	odd	ok
198.0883	2	C8H8B2N4O	198.0879	-0.5	193.7	2	0.00	8.5	odd	ok
198.0883	3	C9H15BO2S	198.0880	-0.3	231.6	3	0.00	5.5	odd	ok
216.0990	1	C8H18B2OS2	216.0980	-3.2	151.5	1	100.00	5.5	odd	ok
216.0990	2	C8H10B2N4O2	216.0984	-1.2	179.3	2	14.12	7.5	odd	ok
216.0990	3	C15H11BN	216.0979	-3.8	230.5	3	0.10	12.0	even	ok
216.0990	4	C9H17BO3S	216.0986	-1.0	246.9	4	0.03	4.5	odd	ok
243.1576	1	C10H21B2O5	243.1570	-0.9	16.7	1	100.00	2.0	even	ok
243.1576	2	C11H17B2N4O	243.1583	4.7	18.9	2	59.18	7.0	even	ok

Figure 2. GC-MS detection of NepB–O–Bnep at 3.3 min, $[M+H]^+$ = 243.1576.

B. Detection of *p*-tolyl disulfide (by-product)

p-Tolyl disulfide was detected by ^{1}H-NMR of the crude reaction mixture obtained using B_2nep_2 as the diboron source, 4-CNPhPy as the pyridine catalyst, and imine **1a** (Figure 3). The ratio observed in the crude was determined by integration using CH_2Br_2 as internal standard. **^{1}H-NMR (300 MHz, $CDCl_3$)** δ (ppm) 7.37 (d, *J* = 8.0 Hz, 4H), 7.09 (d, *J* = 8.0 Hz, 4H), 2.30 (s, 6H)

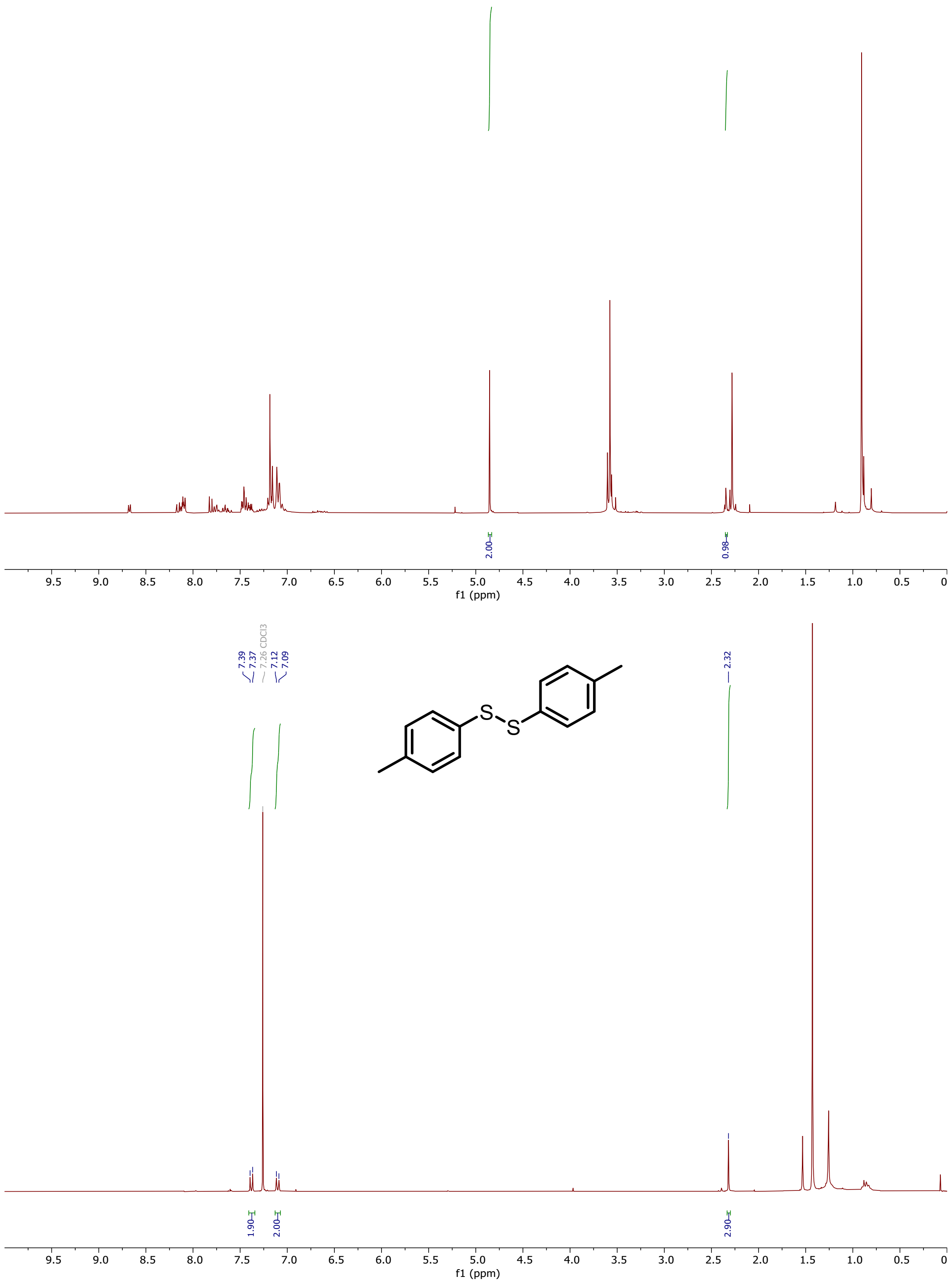

Figure 3. ^{1}H-NMR of *p*-tolyl disulfide in a crude (up). ^{1}H-NMR of *p*-tolyl disulfide isolated (down).

p-Tolyl disulfide was also identified by GC-MS analysis of the crude reaction mixture obtained using B_2nep_2 as the diboron source, 4-CNPhPy as the pyridine catalyst, and imine **1a**. The compound was detected at 8.8 min with a molecular ion peak at *m/z* 246.0533 corresponding to M^+ (Figure 4).

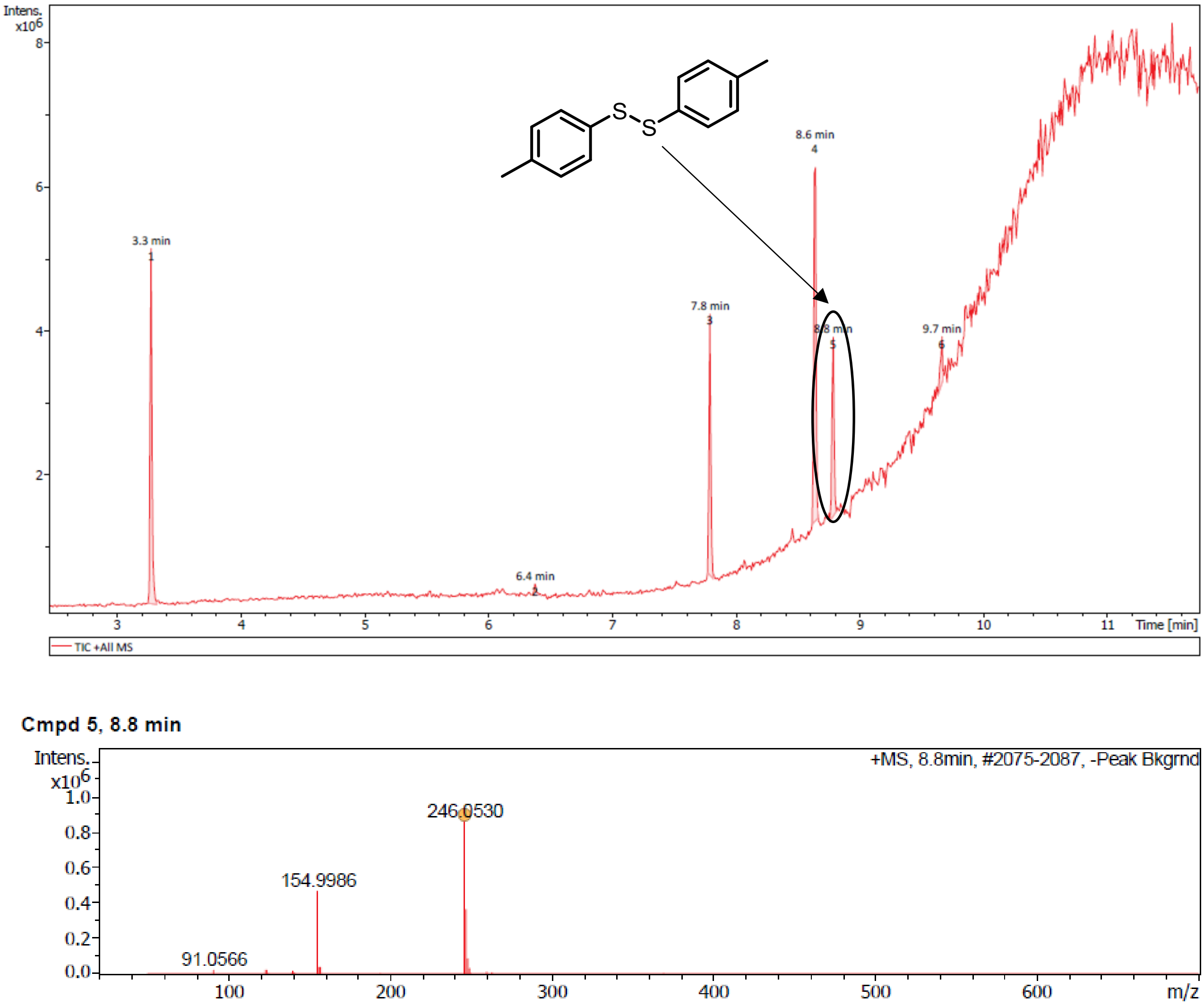

Meas. m/z	#	Ion Formula	m/z	err [ppm]	mSigma	#mSigma	Score	rdb (neutral)	e⁻ Conf	N-Rule
246.0530	1	C14H14S2	246.0531	0.5	127.5	1	100.00	12.5	odd	ok
246.0530	2	C14H6N4O	246.0536	2.4	155.8	2	10.36	14.5	odd	ok
246.0530	3	C12H4N7	246.0523	-3.1	162.6	3	5.89	15.0	even	ok
246.0530	4	C13H10O5	246.0523	-3.1	167.9	4	4.07	9.5	odd	ok
246.0530	5	C6H10N6O3S	246.0530	-0.3	189.9	5	1.04	7.5	odd	ok

Figure 4. GC-MS detection of *p*-tolyl disulfide (Tol–S–S–Tol) at 8.8 min, M^+ = 246.0533.

C. Detection of Intermediates IV.

The detection of a molecular ion at *m/z* 476.2066 ([M+H]$^+$) by mass spectrometry (APCI, direct sample introduction at 250 °C) supports the proposed mechanism (Figure 5). This signal corresponds to the hydrogenated form of a radical intermediate structurally equivalent to **IVa(5)** or **IVa(6)** (Figure 1 in the main text), resulting from hydrogen atom transfer (HAT), as expected given the radical nature of these species. Although the DFT calculations were performed using simplified models (B_2pin_2 and a phenyl-substituted imine), the experimentally observed intermediate—obtained from a reaction using B_2nep_2, 4-CNPhPy, and imine **1a**—can be considered the corresponding experimental analogue. Notably, the signal intensity was higher after 1 hour than after 18 hours of reaction, indicating that this species is transient. This observation supports the formation of **IVa-type** radical intermediates and aligns with the proposed reversibility of the five-membered pathway. The data are consistent with a scenario in which **IVa(6)**, stabilized by a benzylic radical, undergoes sulfonyl radical elimination to afford the thermodynamic product, whereas **IVa(5)**, being less stable, is more prone to revert.

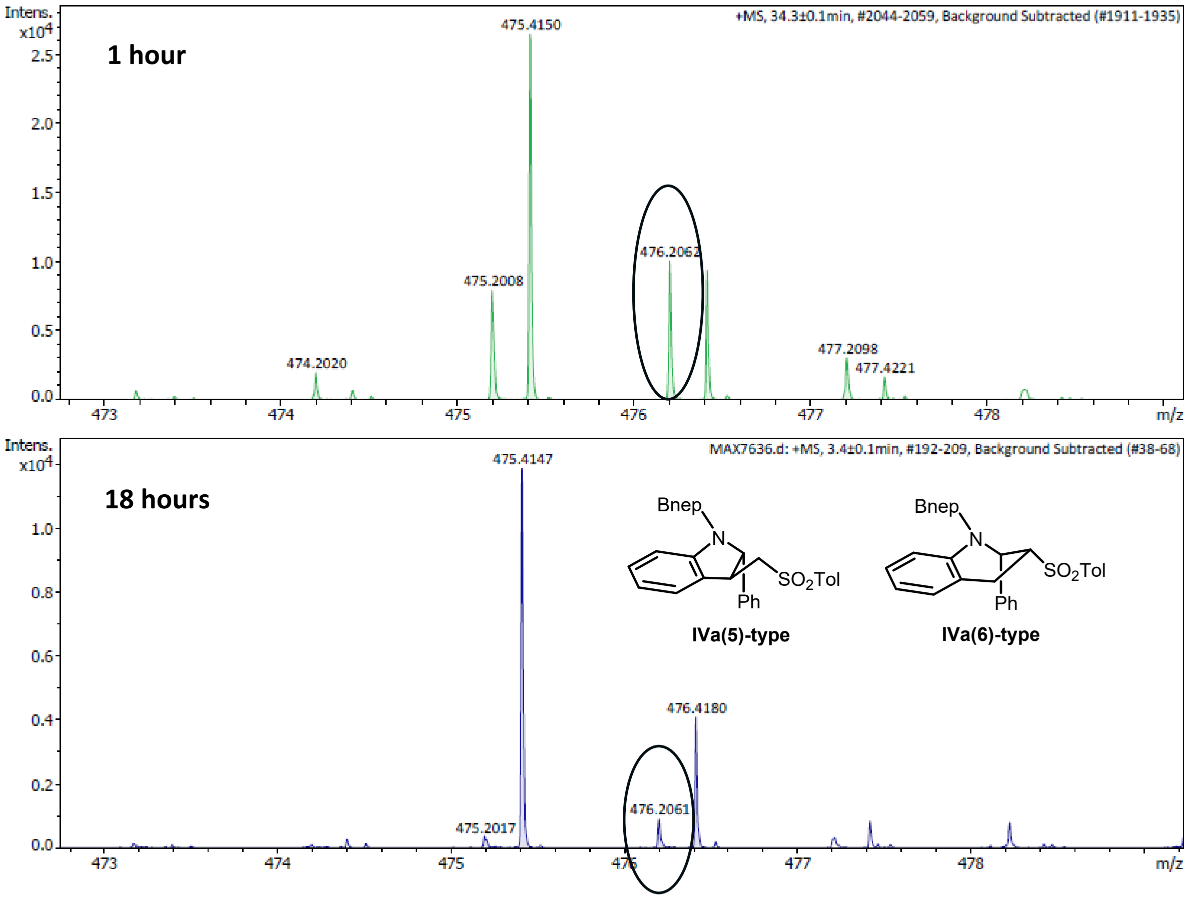

Figure 5. APCI mass spectra (1 h and 18 h) showing the signal at *m/z* 476.2066 ([M+H]$^+$), assigned to the hydrogenated form of a radical intermediate of type **IVa(5)/IVa(6)**.

7.- Experimental spectra

(*E*)-2-(2-Tosylvinyl)aniline (SM1)

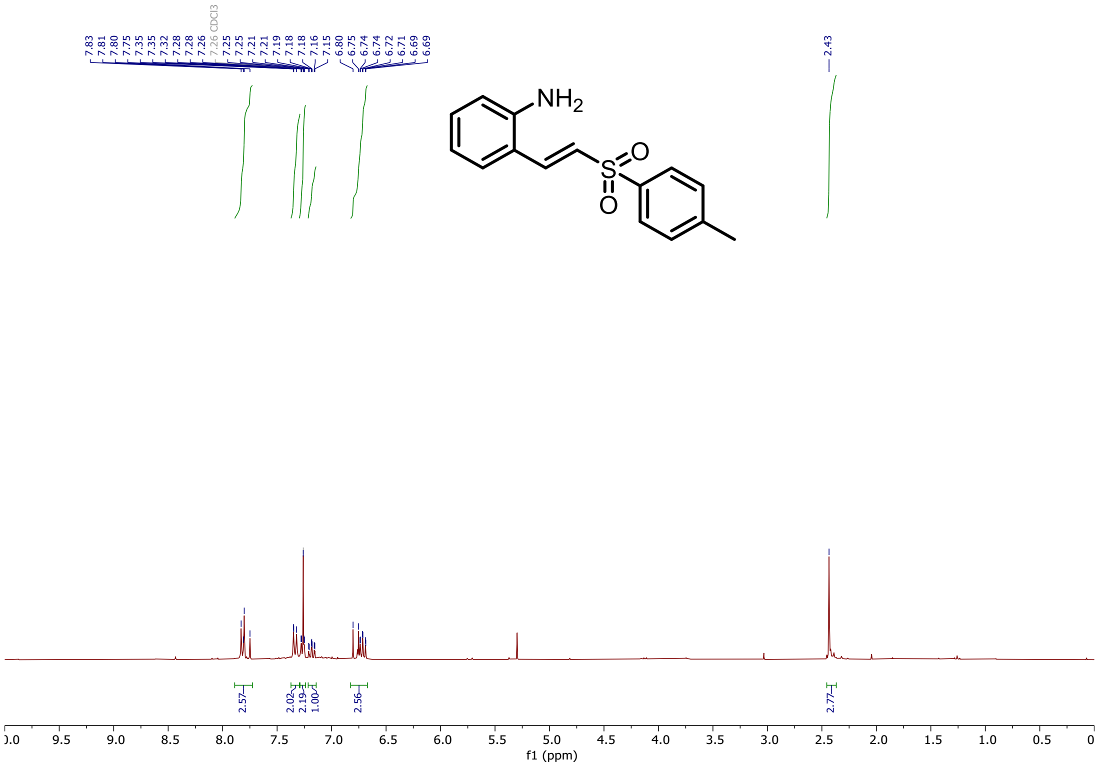

SM1 (^{13}C-NMR)

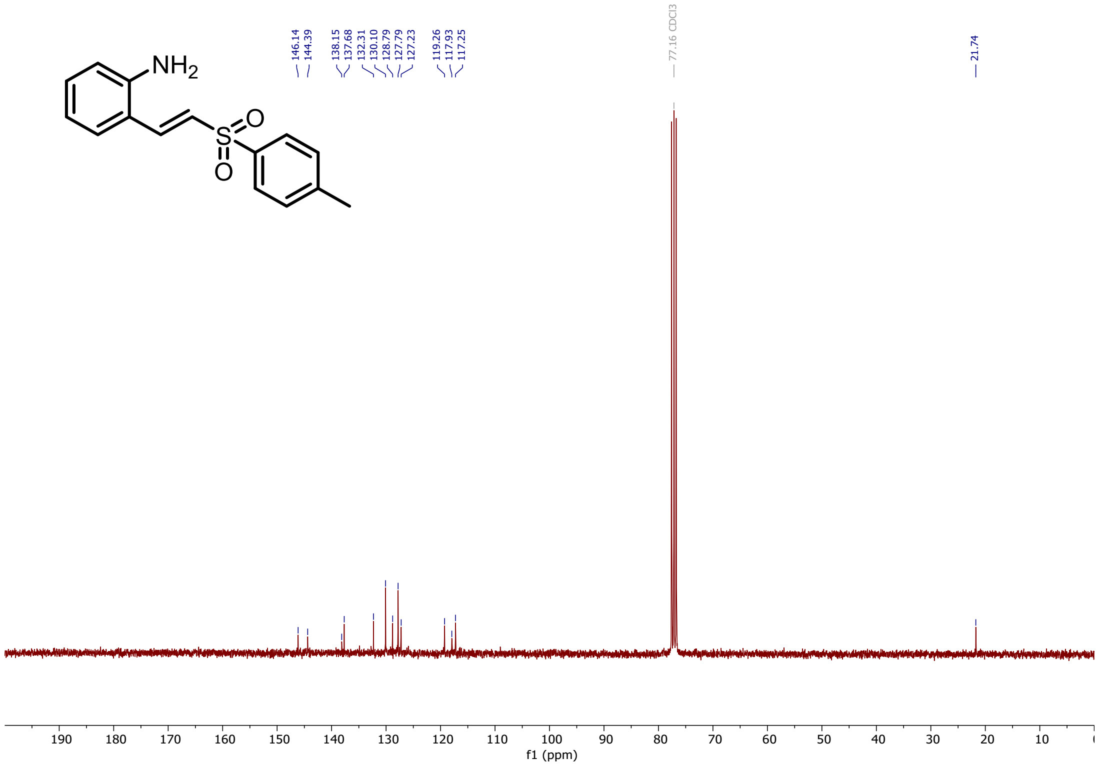

(*E*)-5-Bromo-2-(2-tosylvinyl)aniline (SM2)

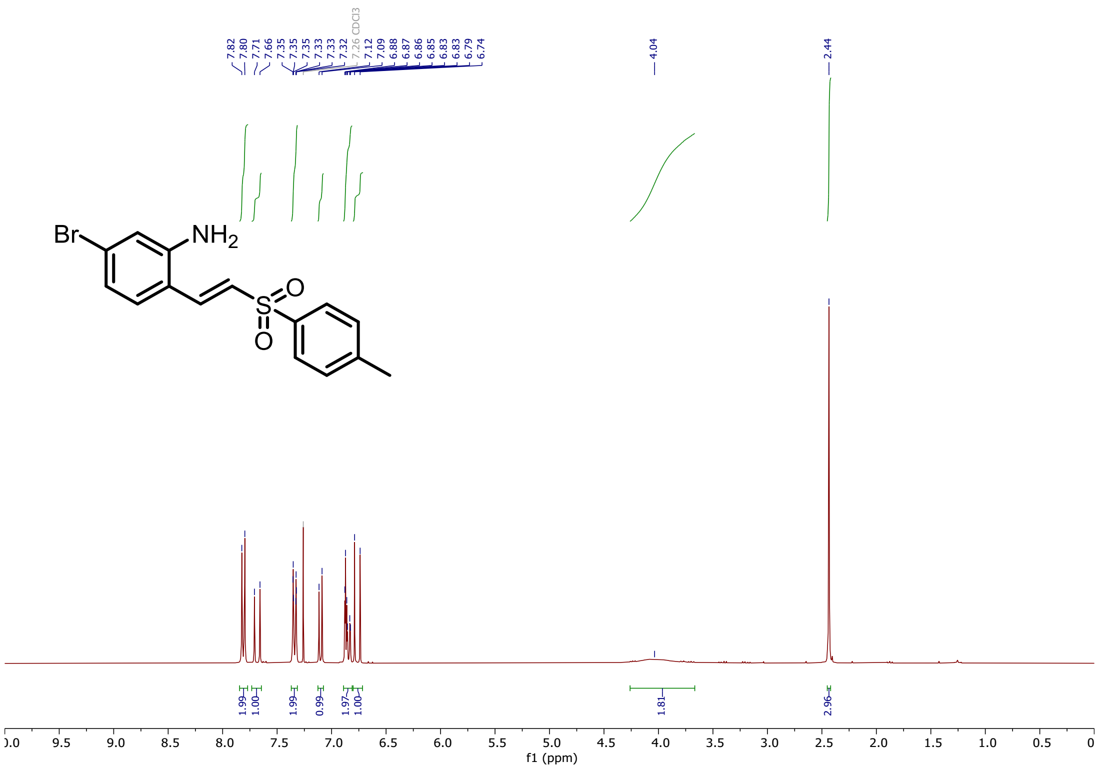

SM2 (^{13}C-NMR)

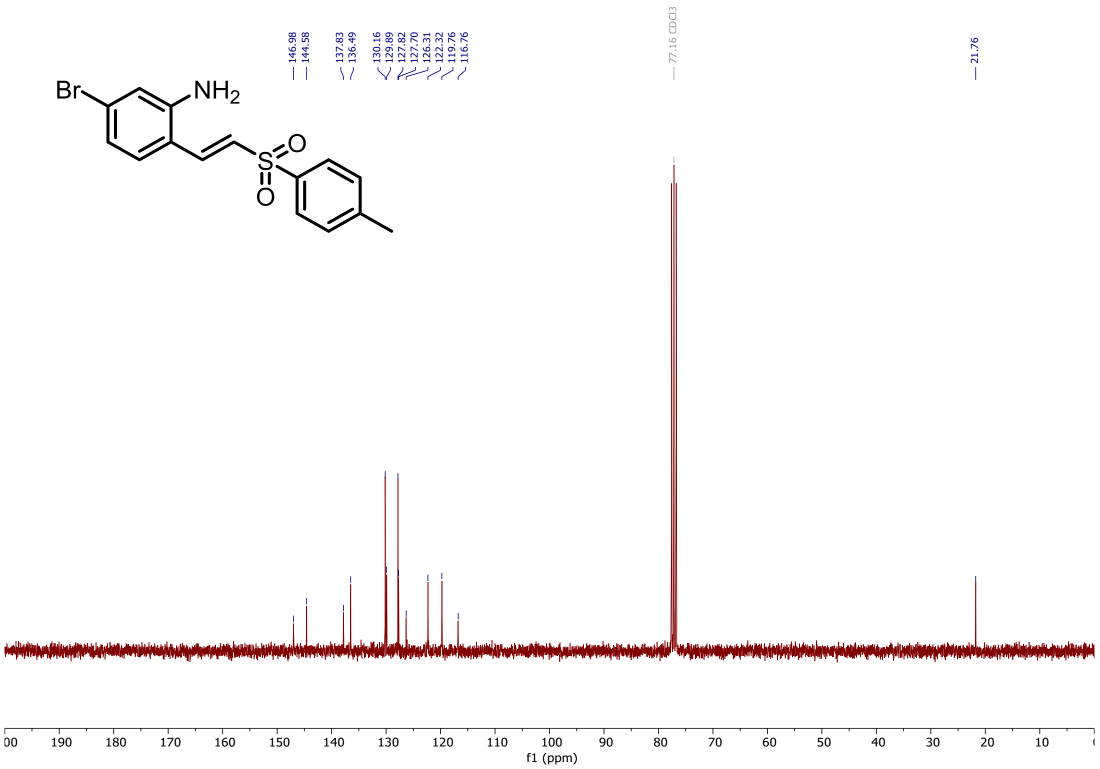

(*E*)-6-(2-Tosylvinyl)benzo[*d*][1,3]dioxol-5-amine (SM3)

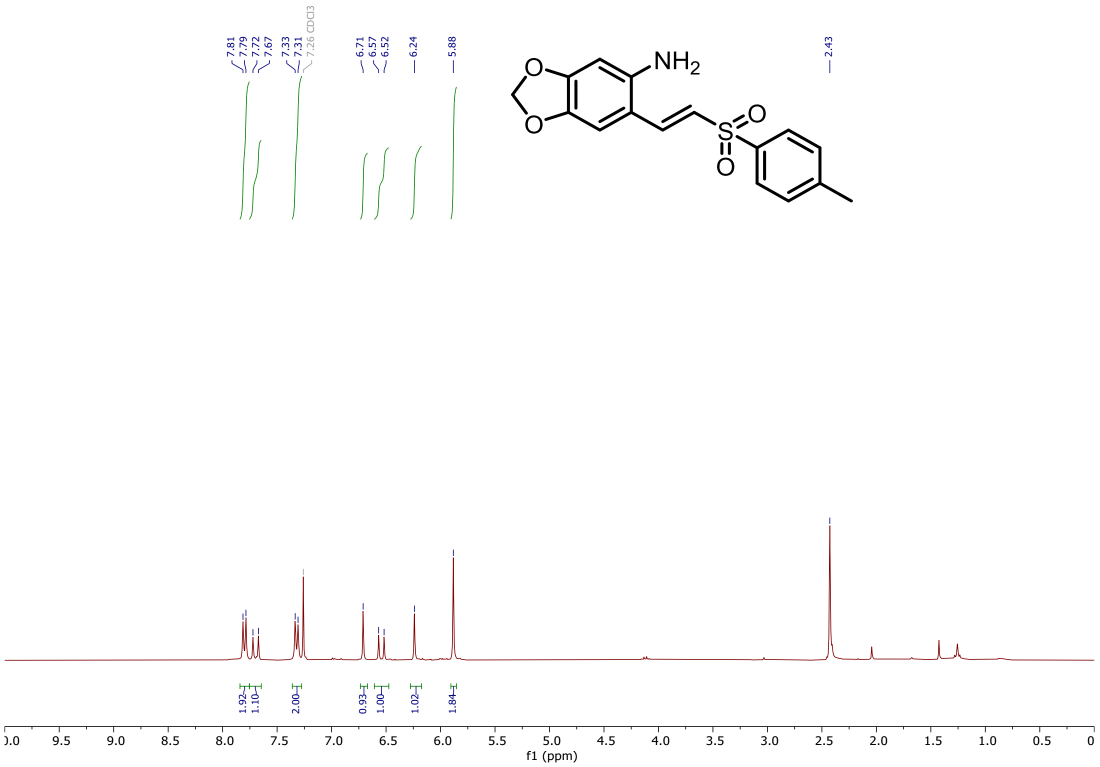

SM3 (^{13}C-NMR)

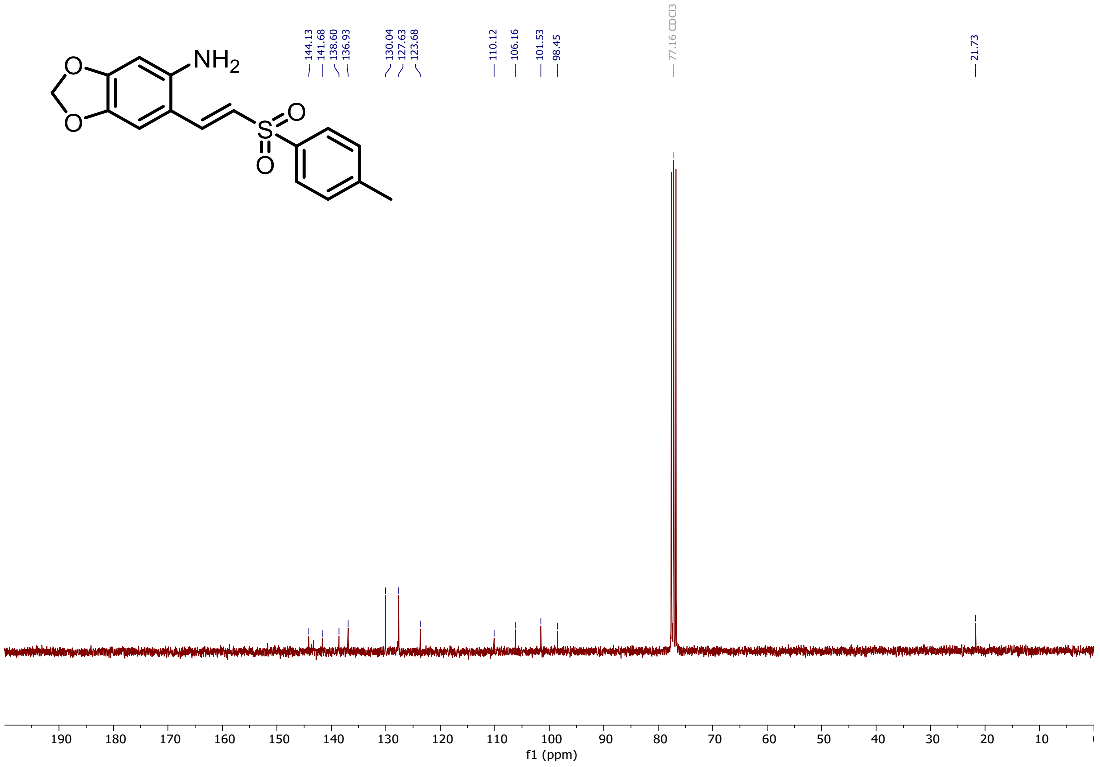

(*Z*)-4-Methoxy-1-nitro-2-(2-tosylvinyl)benzene + (*E*)- 4-methoxy-1-nitro-2-(2-tosylvinyl)benzene (SM4)

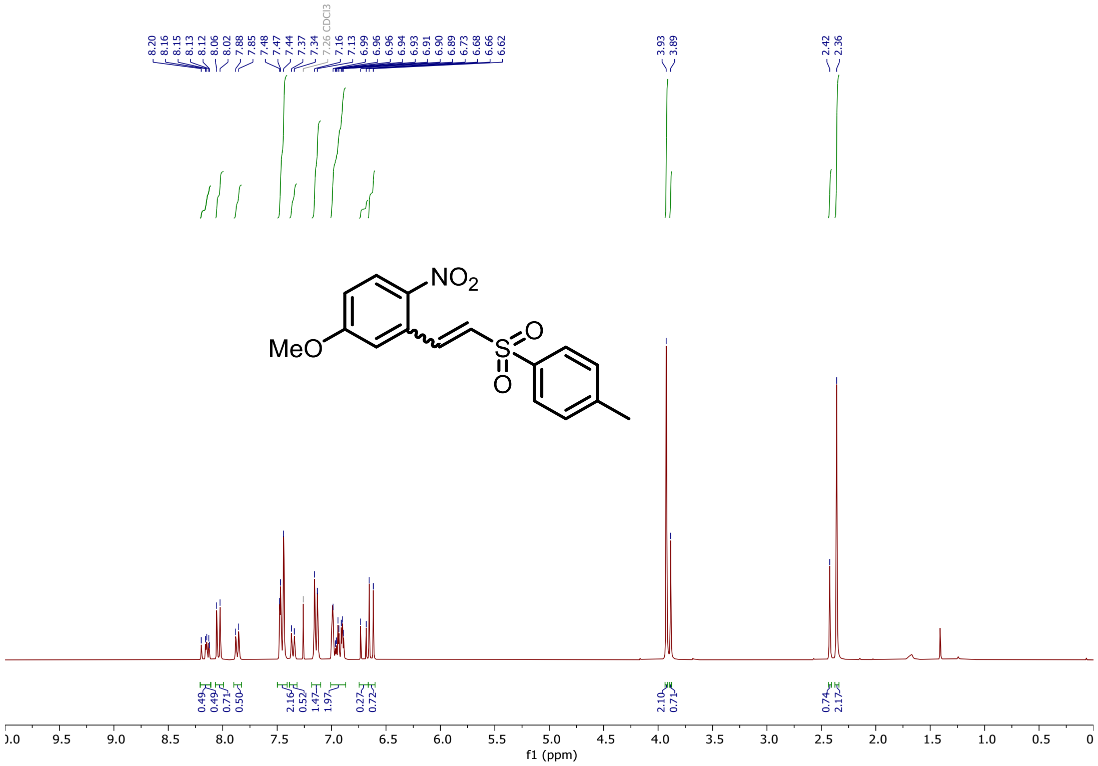

SM4 (^{13}C-NMR)

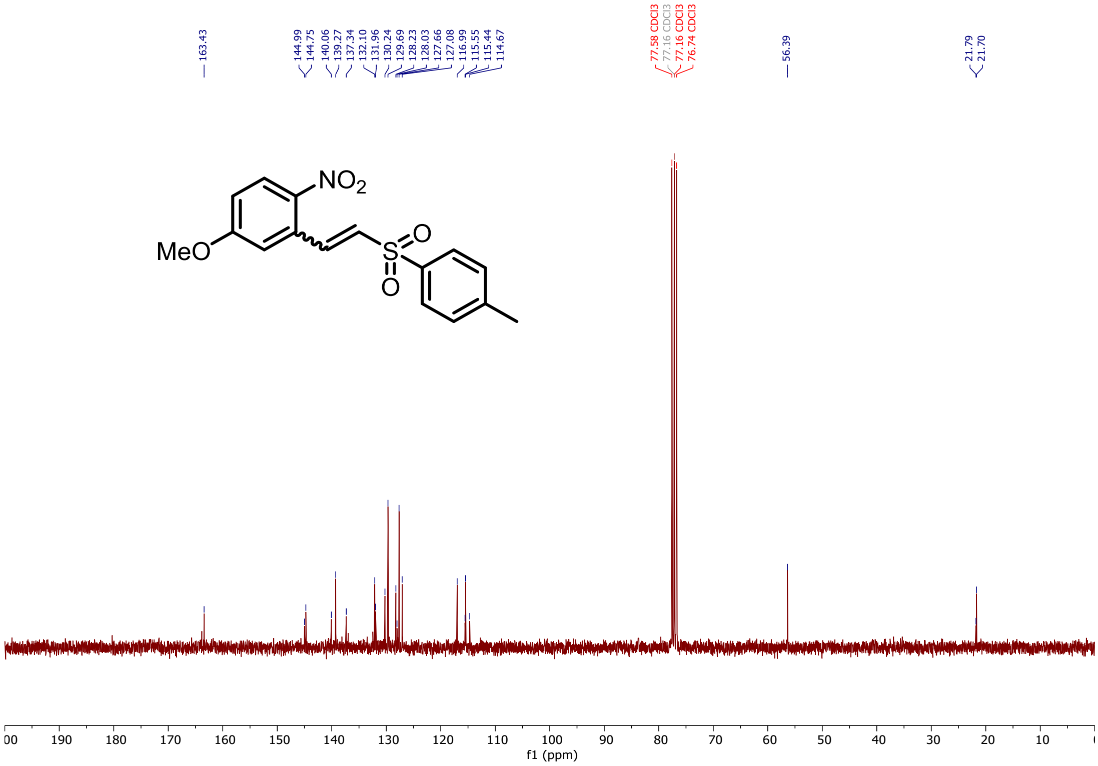

(*Z*)-4-Chloro-1-nitro-2-(2-tosylvinyl)benzene + (*E*)- 4-chloro-1-nitro-2-(2-tosylvinyl)benzene (SM5)

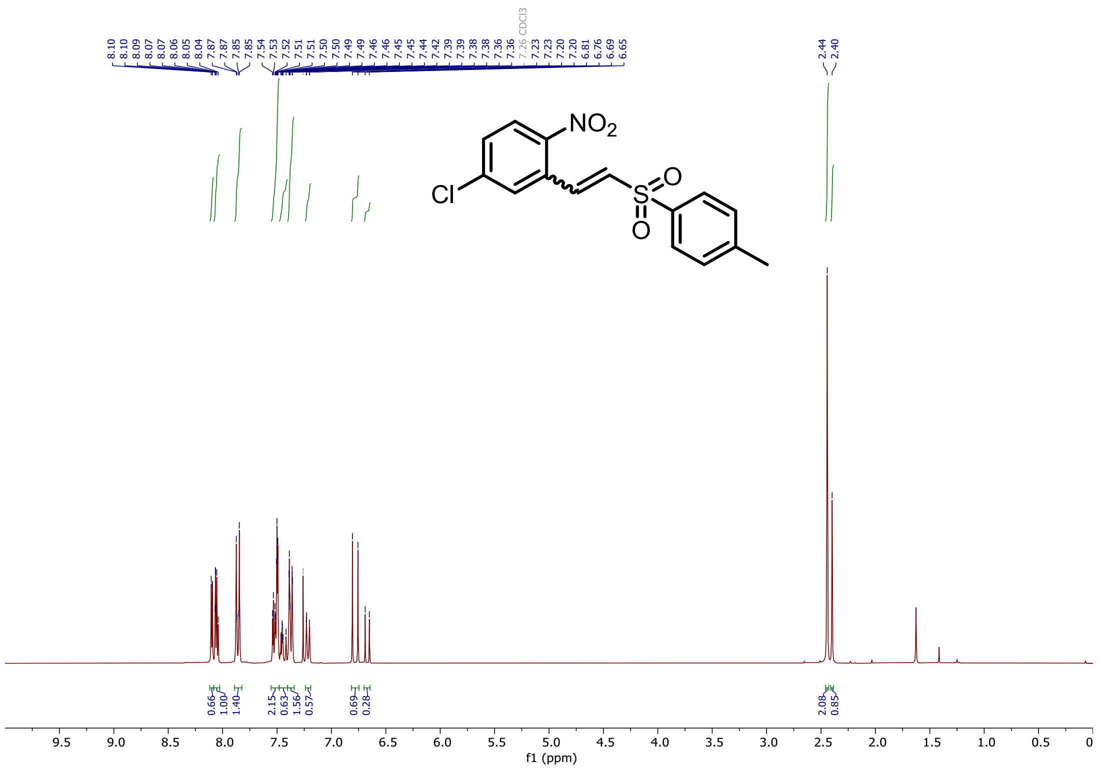

SM5 (^{13}C-NMR)

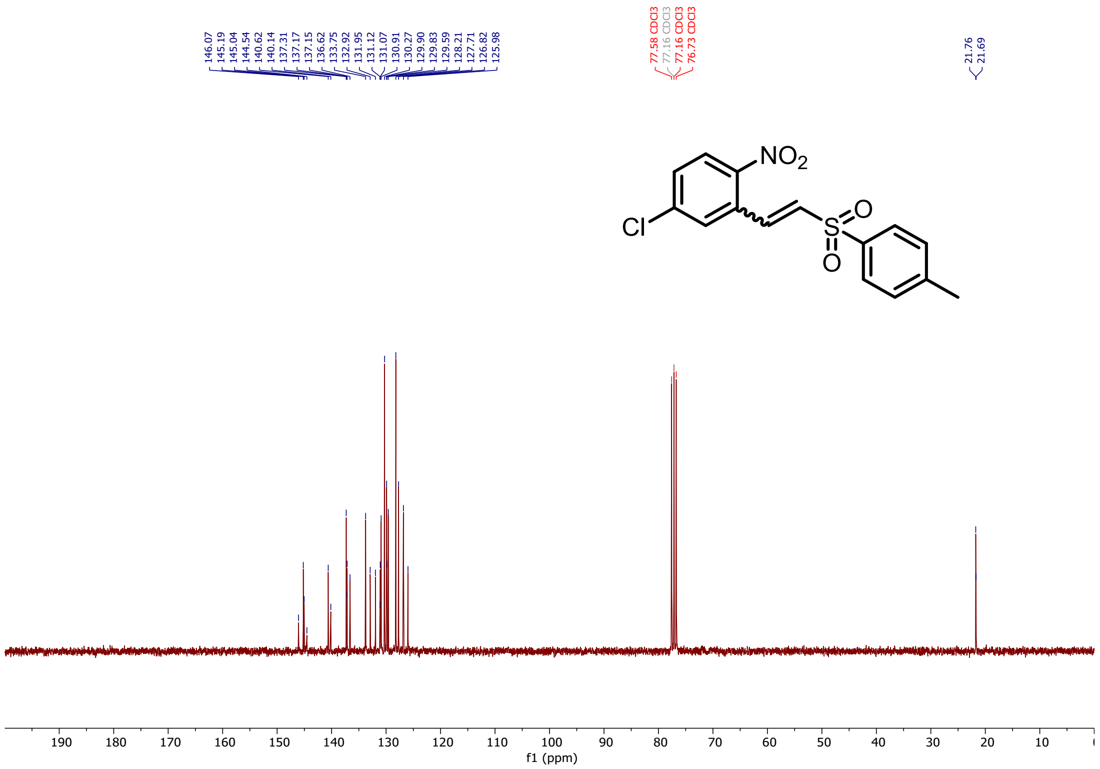

6-Aminobenzo[d][1,3]dioxole-5-carbaldehyde (SM6)

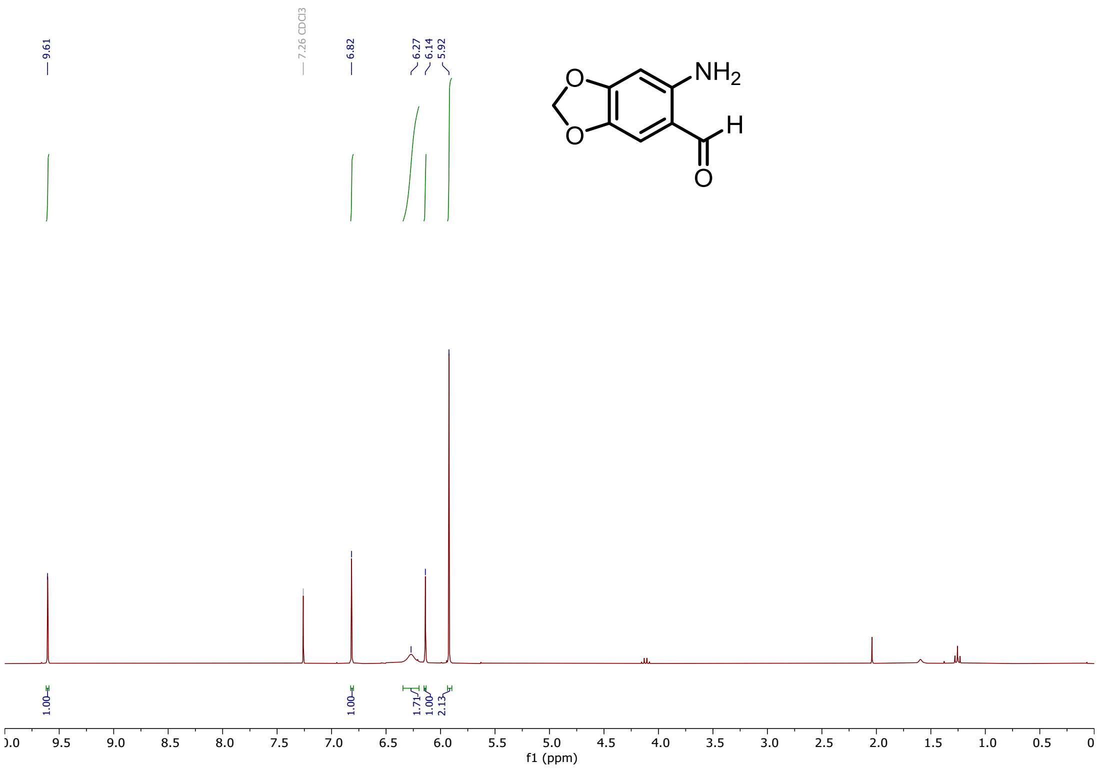

(*Z*)-4-Methoxy-2-(2-tosylvinyl)aniline + (*E*)-4-methoxy-2-(2-tosylvinyl)aniline (SM7)

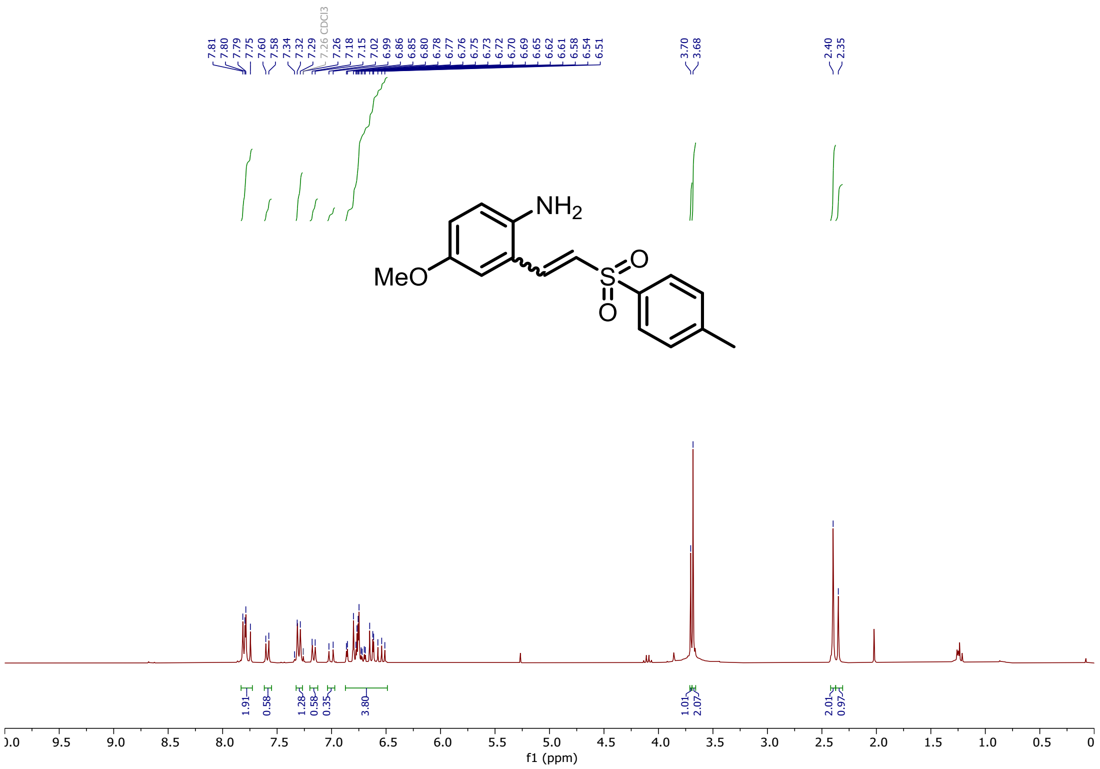

SM7 (^{13}C-NMR)

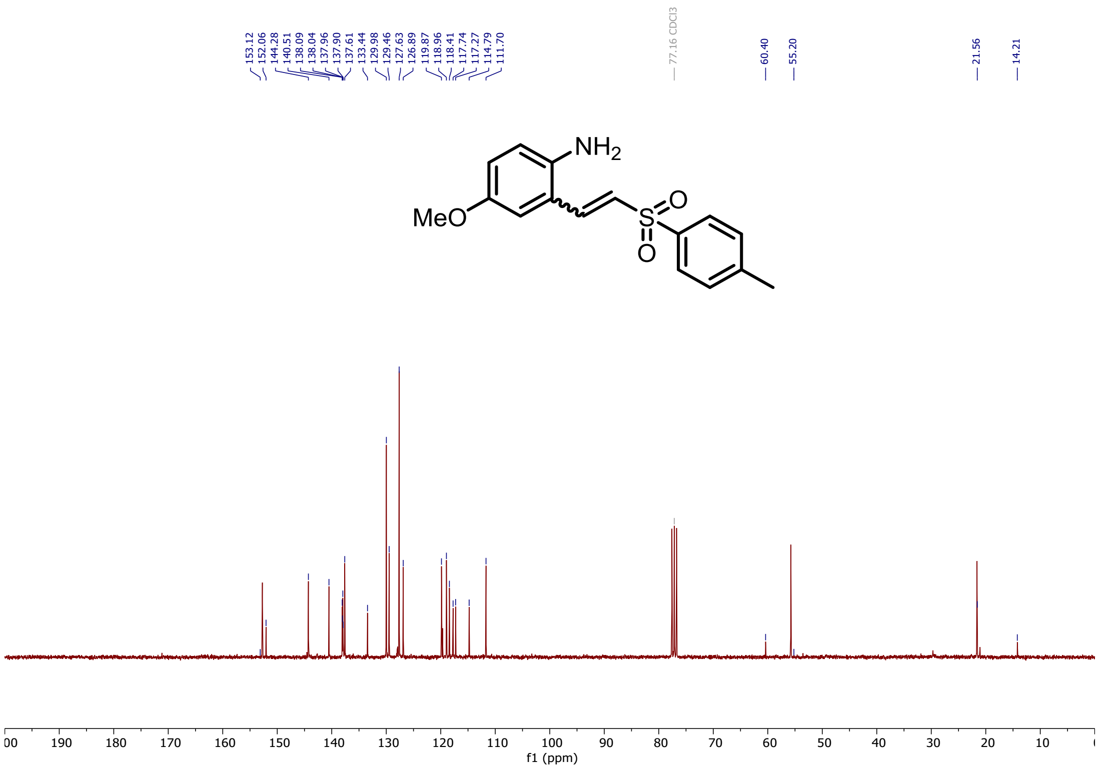

(*E*)-4-Chloro-2-(2-tosylvinyl)aniline (SM8)

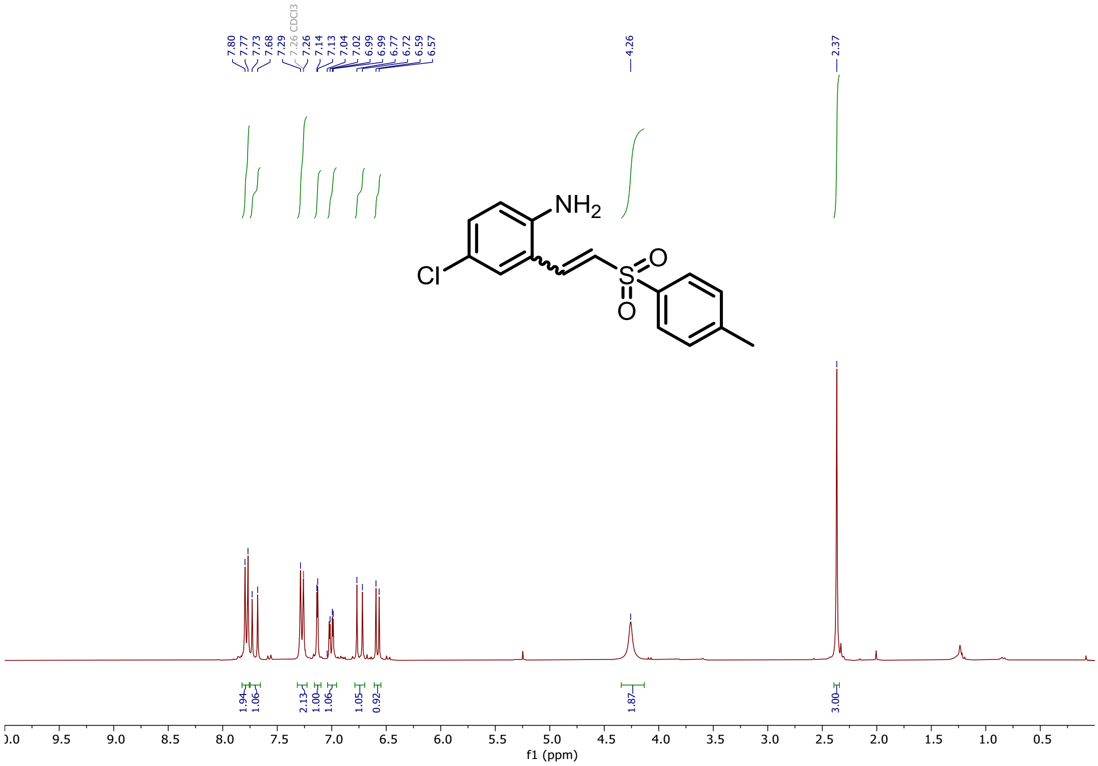

SM8 (^{13}C-NMR)

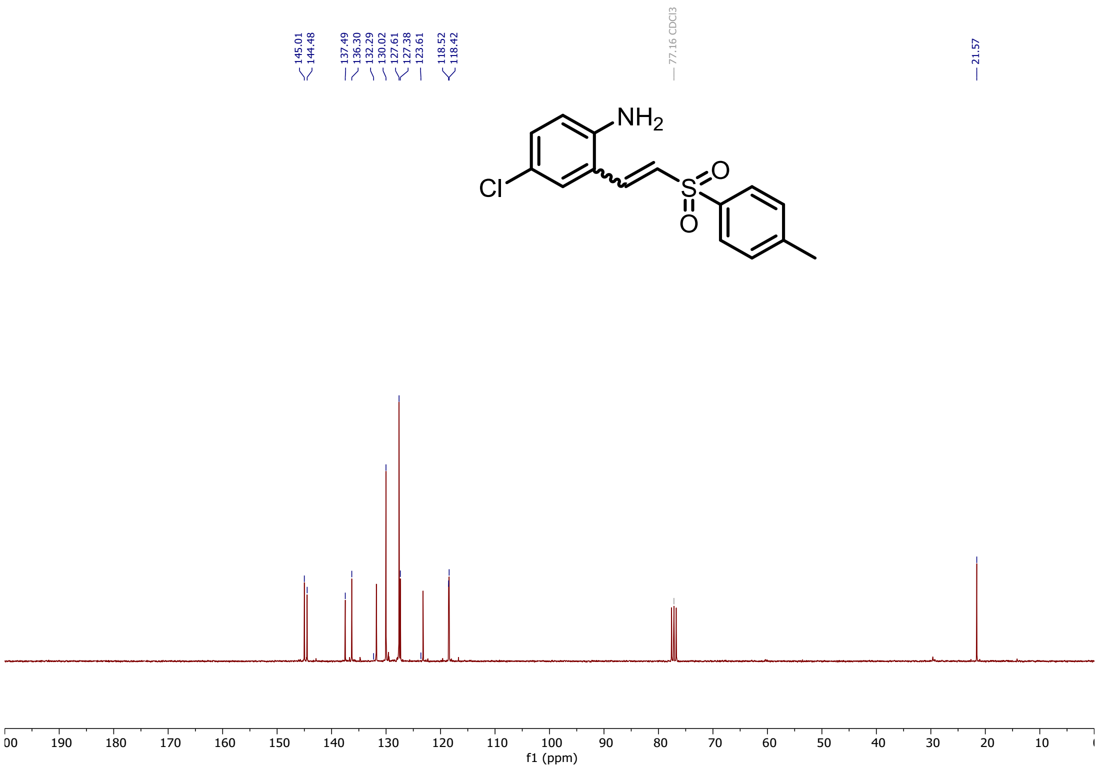

(*E*)-1-Phenyl-*N*-(3-((*E*)-2-tosylvinyl)phenyl)methanimine (1a)

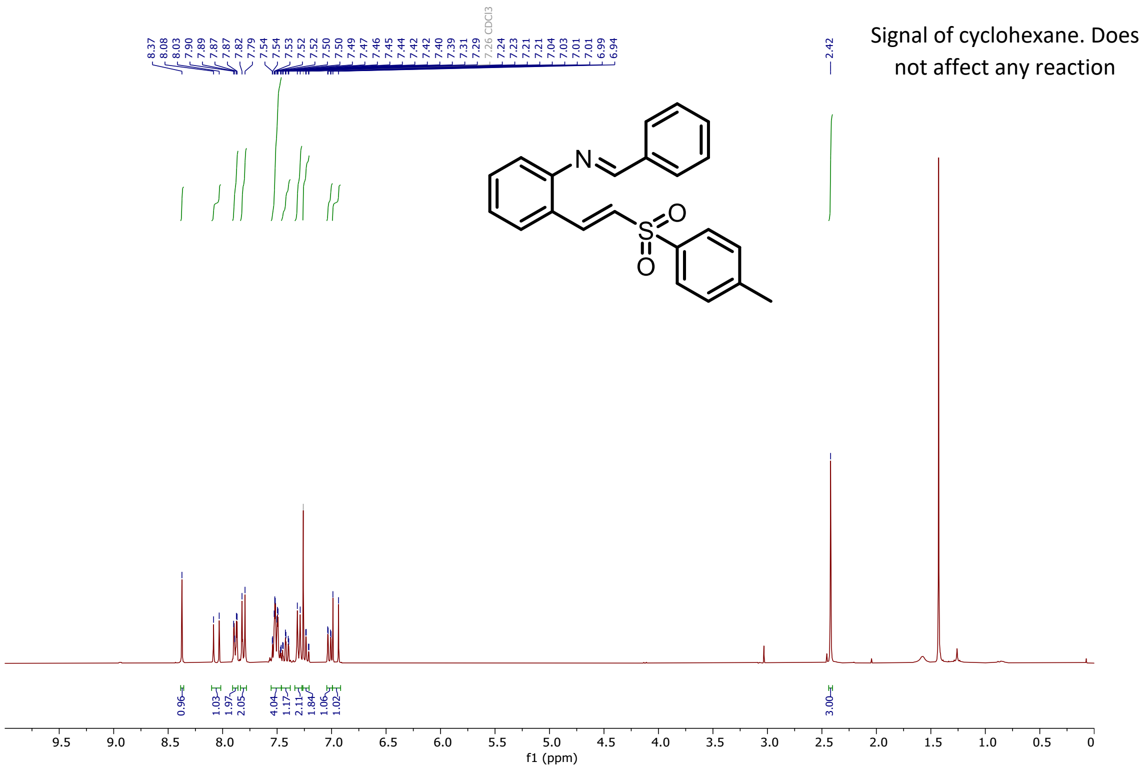

1a (^{13}C-NMR)

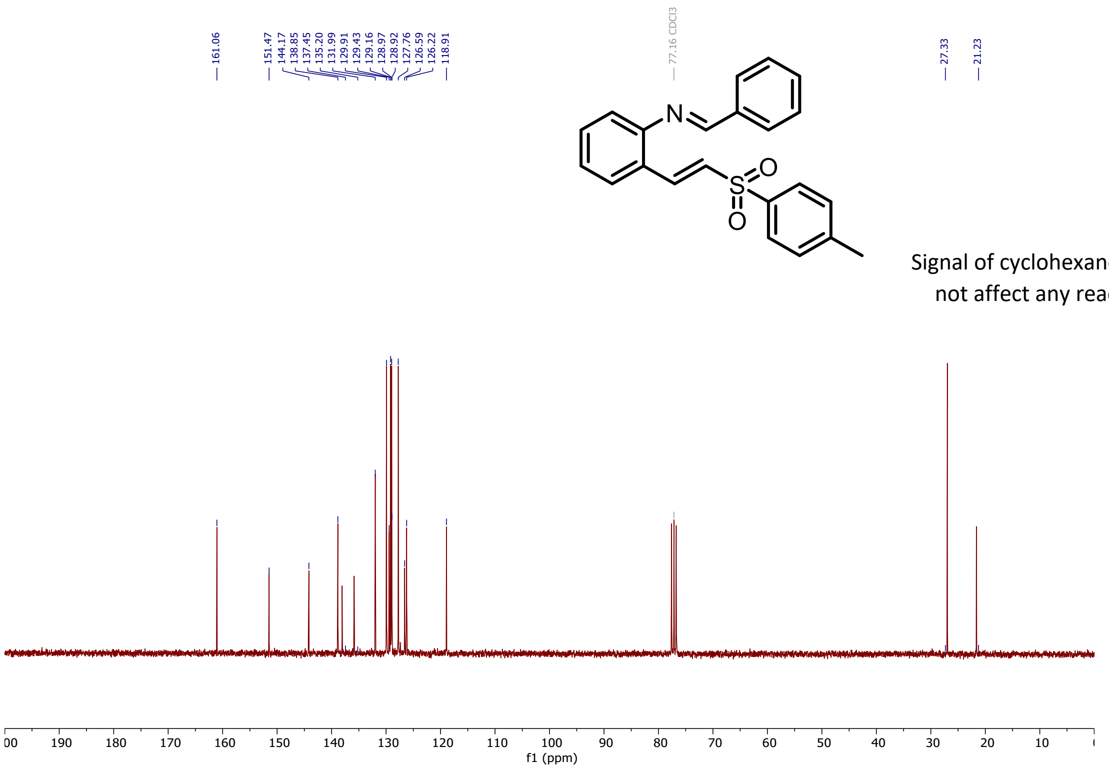

(*E*)-1-*o*-Tolyl-*N*-(2((*E*)-2-tosylvinyl)phenyl)methanimine (1b)

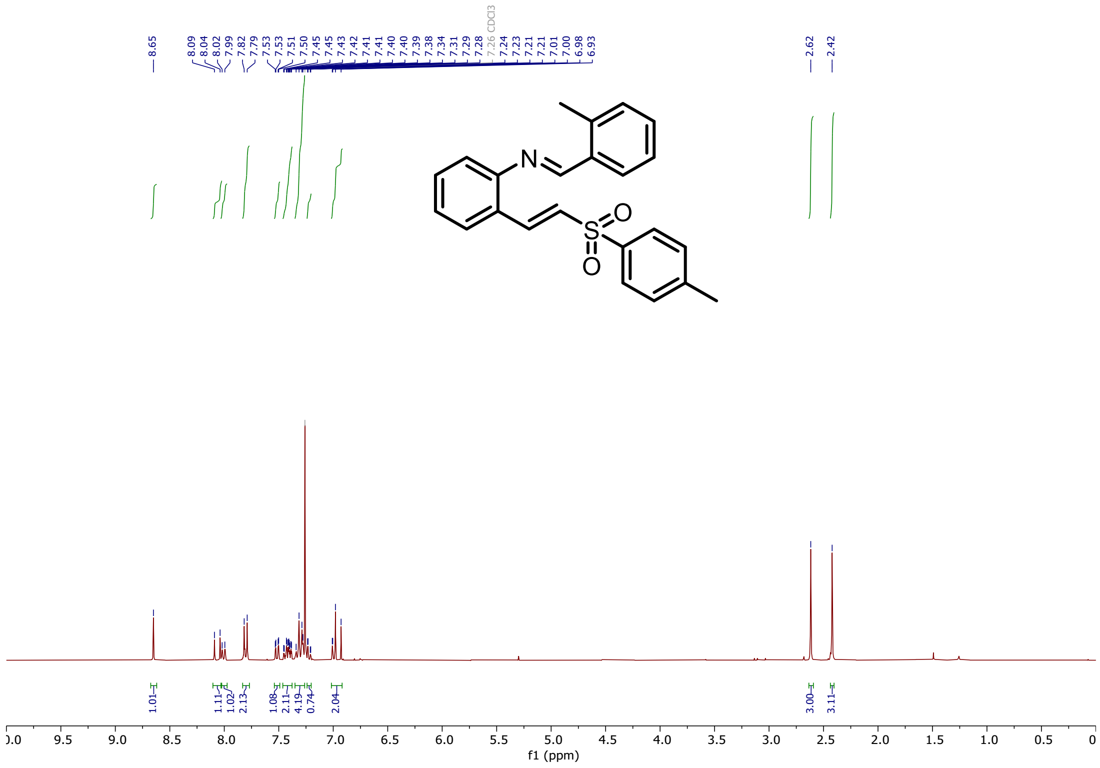

1b (^{13}C-NMR)

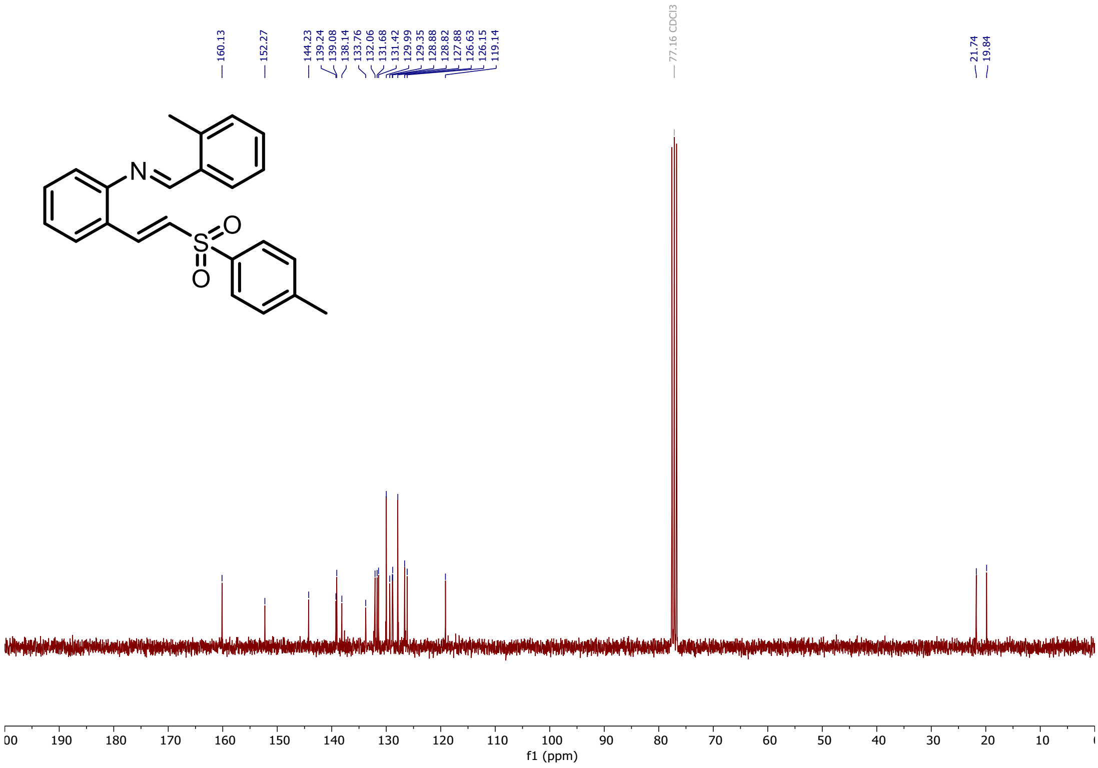

(*E*)-1-(4-Fluorophenyl)-*N*-(2-((*E*)-2-tosylvinyl)phenyl)methanimine (1c)

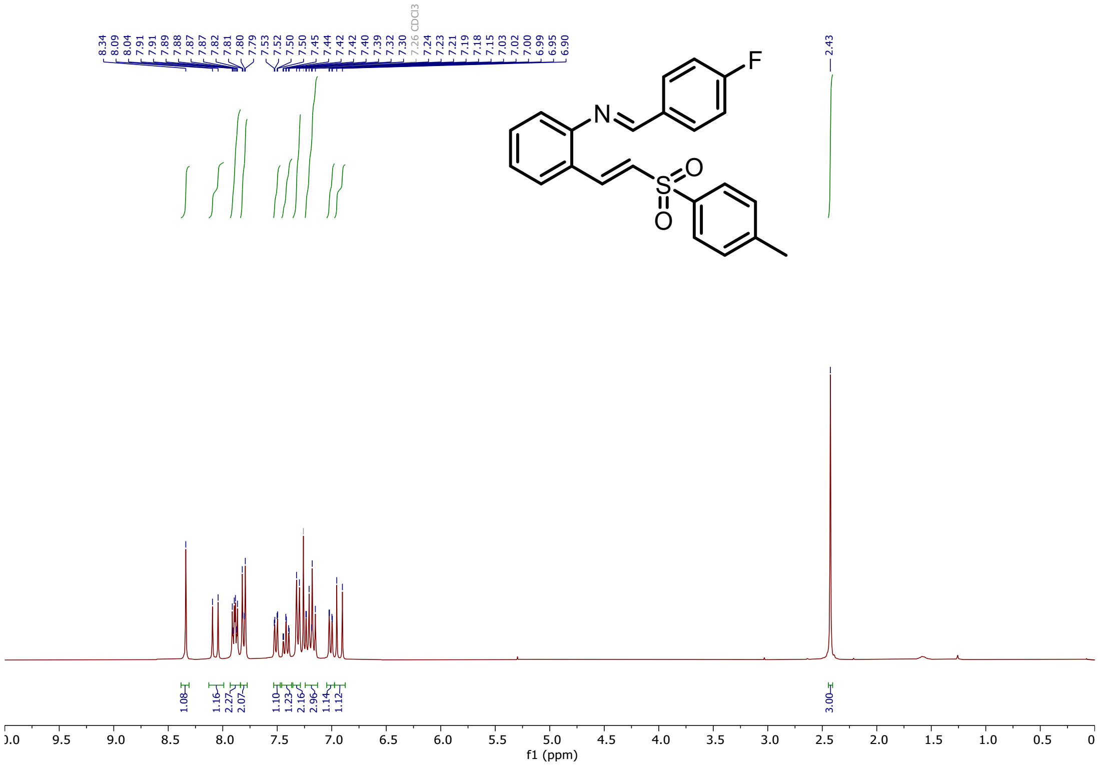

1c (^{13}C-NMR)

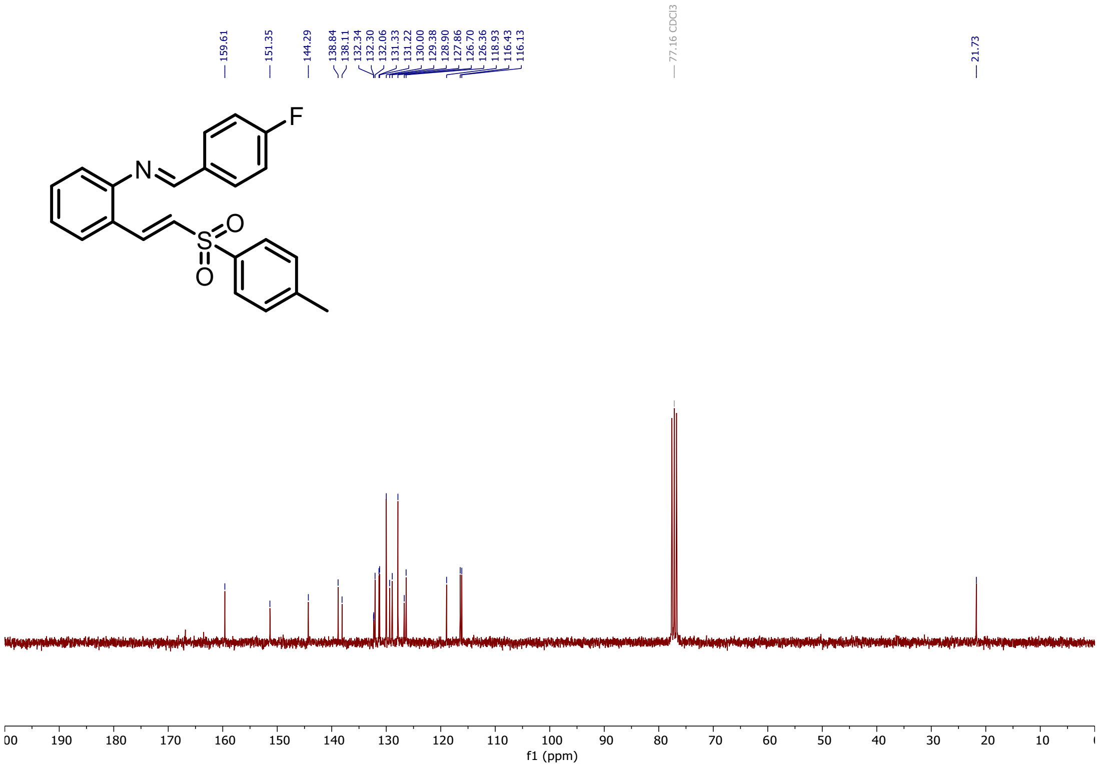

1c (^{19}F-NMR)

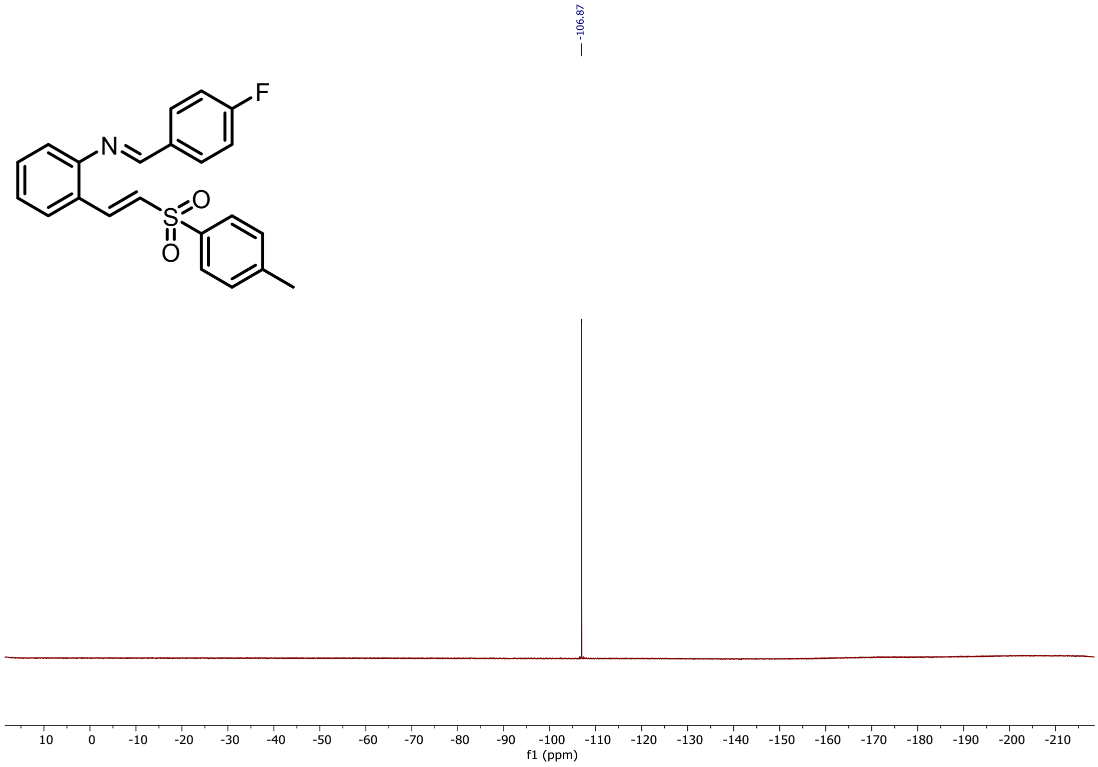

(*E*)-1-(4-Chlorophenyl)-*N*-(2-((*E*)-2-tosylvinyl)phenyl)methanimine (1d)

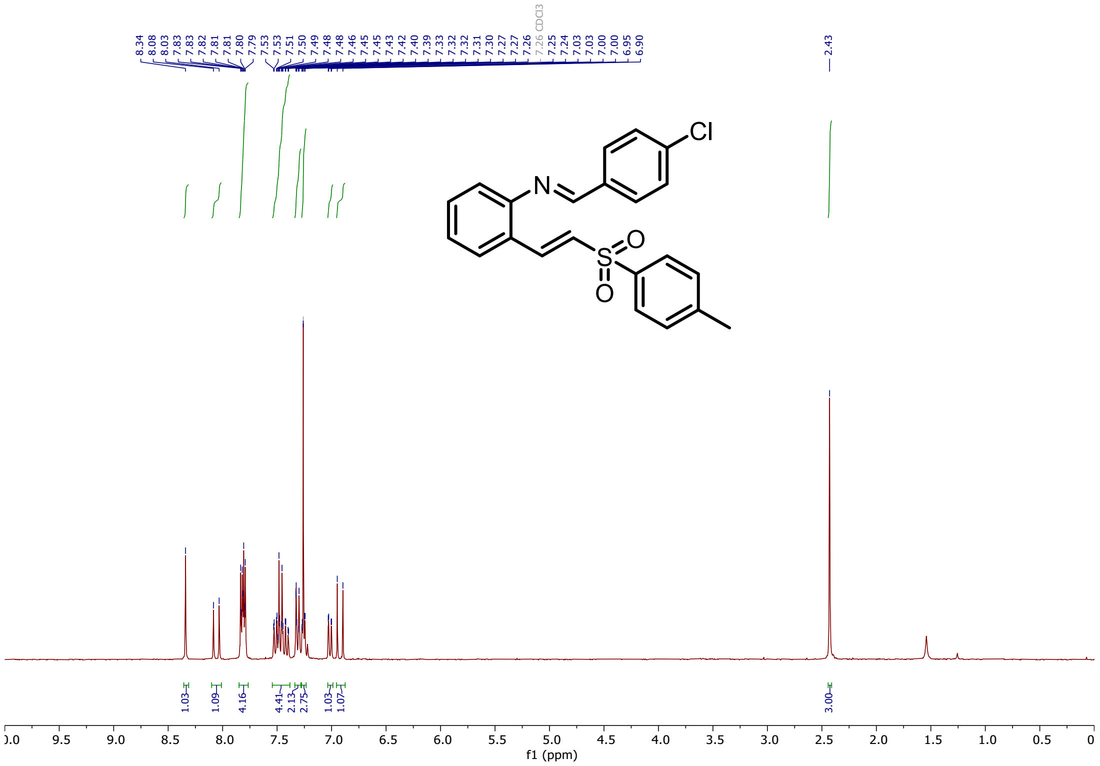

1d (^{13}C-NMR)

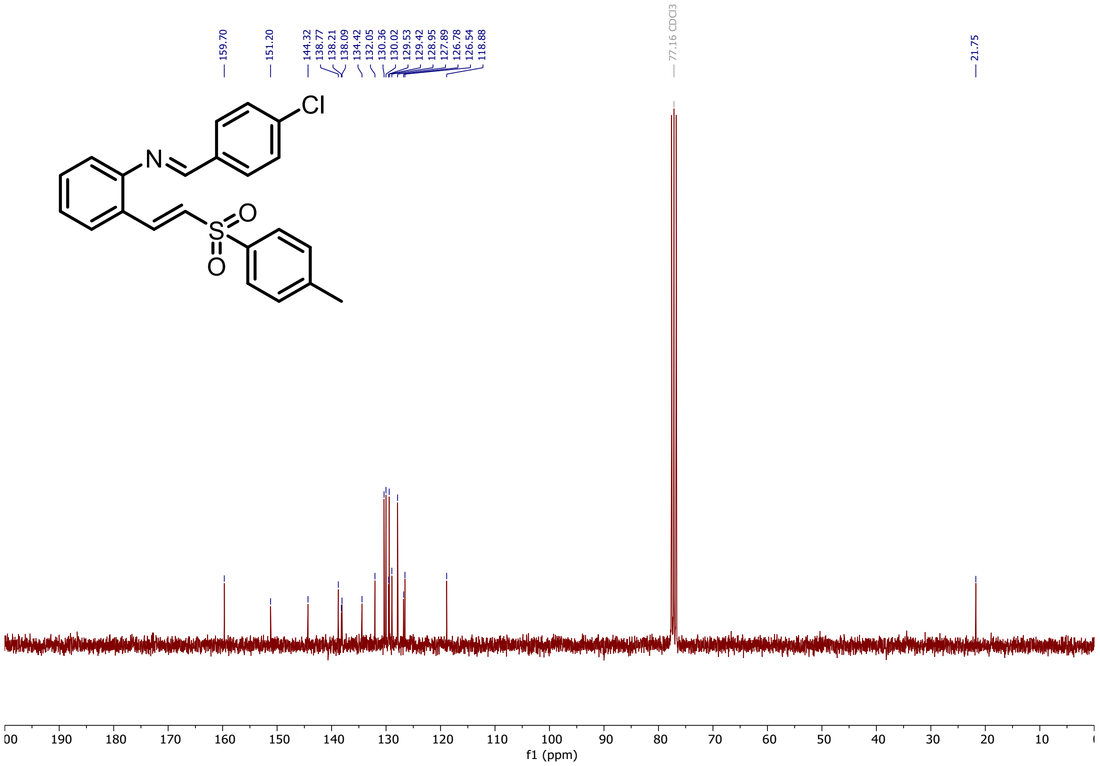

(*E*)-1-(4-Bromophenyl)-*N*-(2-((*E*)-2-tosylvinyl)phenyl)methanimine (1e)

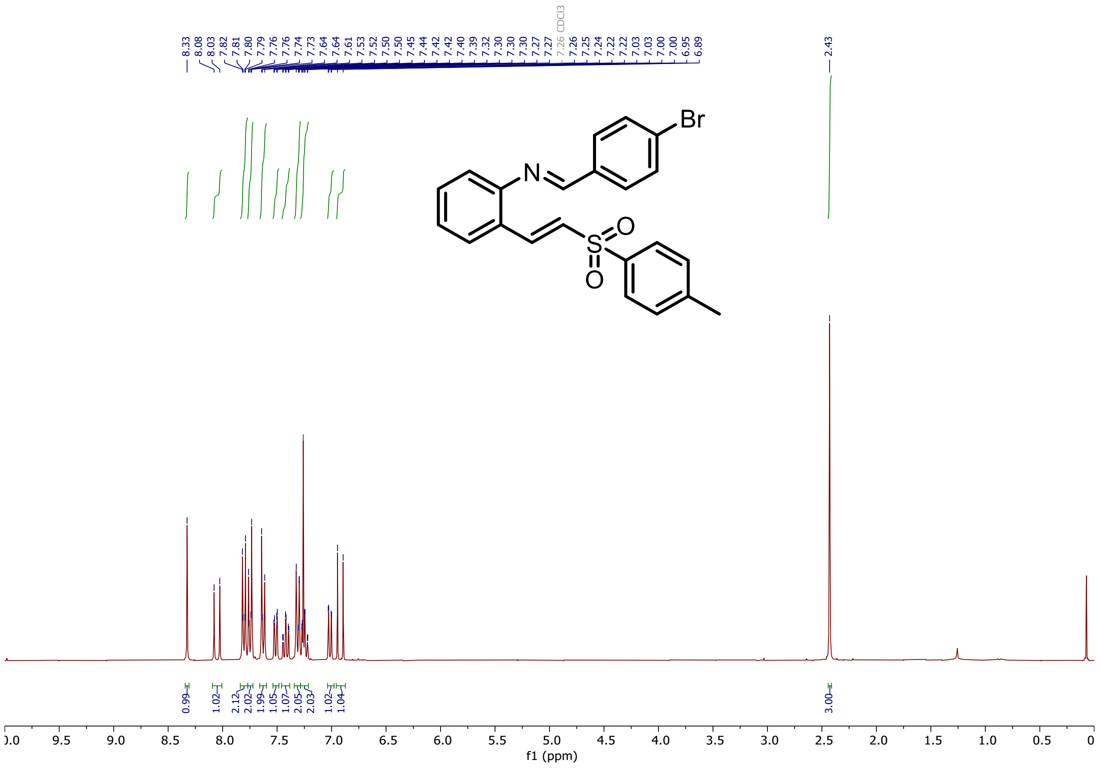

1e (^{13}C-NMR)

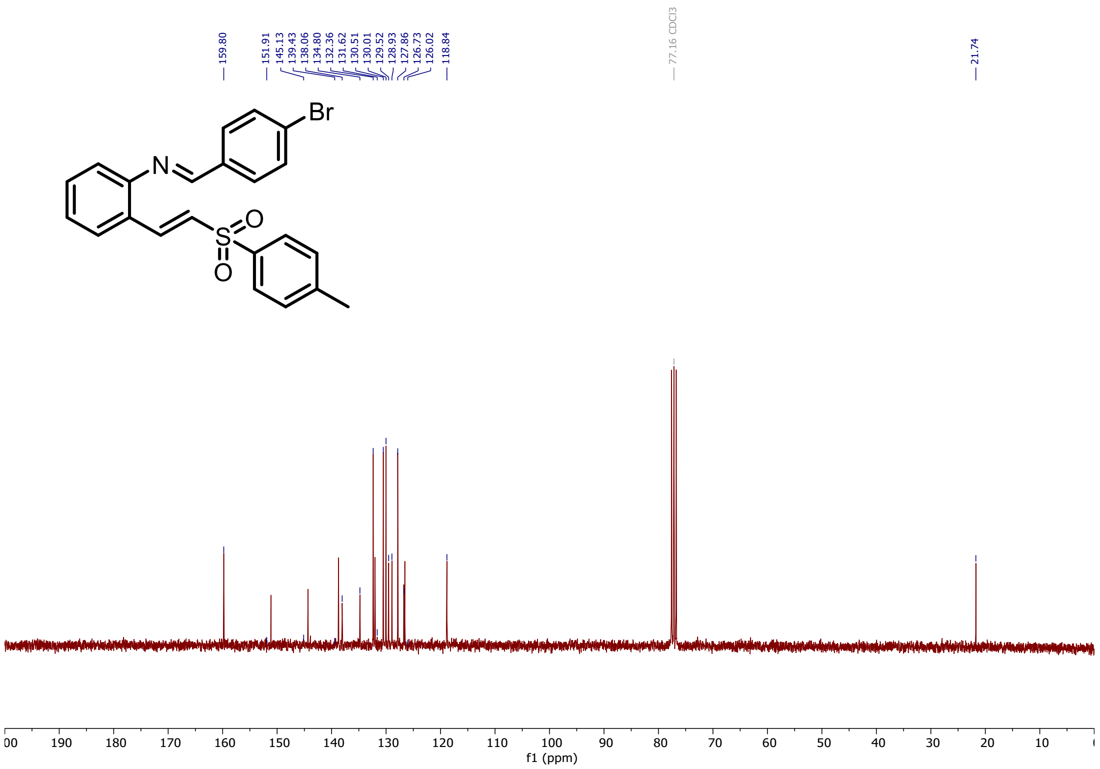

(*E*)-1-*p*-Tolyl-*N*-(2((*E*)-2-tosylvinyl)phenyl)methanimine (1f) (CH_2Br_2 and CH_2Cl_2 are present in the spectrum, but their presence do not affect the yield).

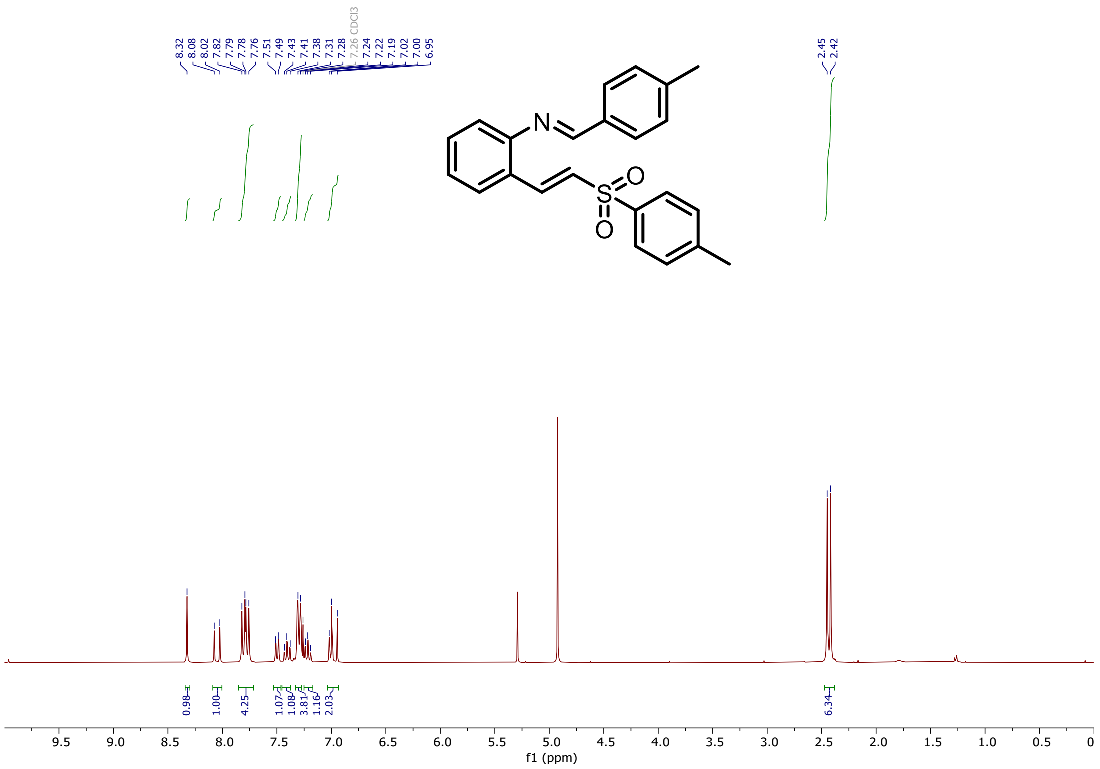

1f (^13^C-NMR)

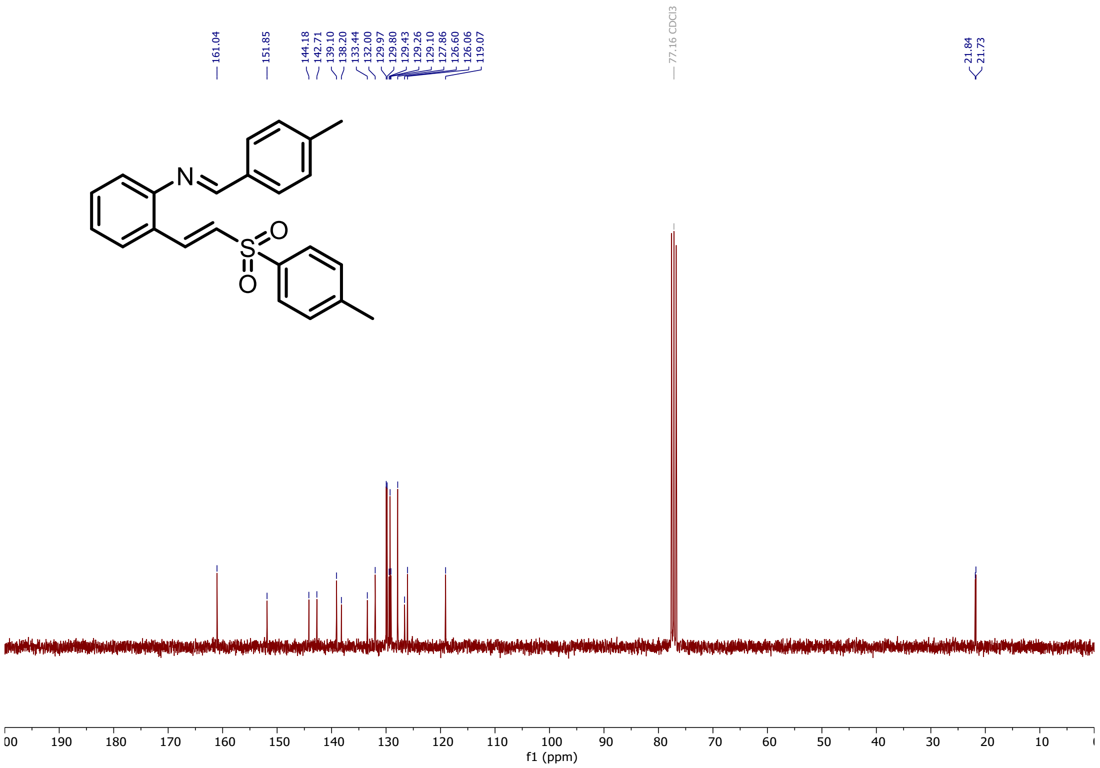

(*E*)-1-(4-Methoxyphenyl)-*N*-(2-((*E*)-2-tosylvinyl)phenyl)methanimine (1g) (CH_2Br_2 and CH_2Cl_2 are present in the spectrum, but their presence do not affect the yield).

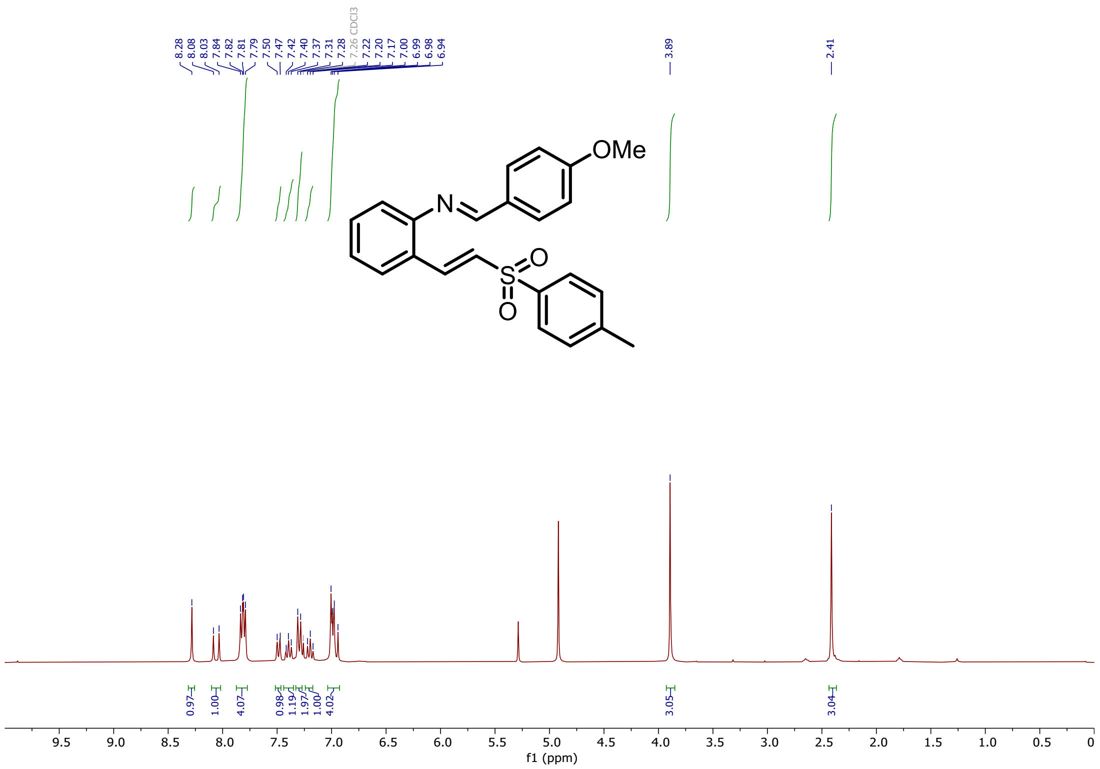

1g (^13C-NMR)

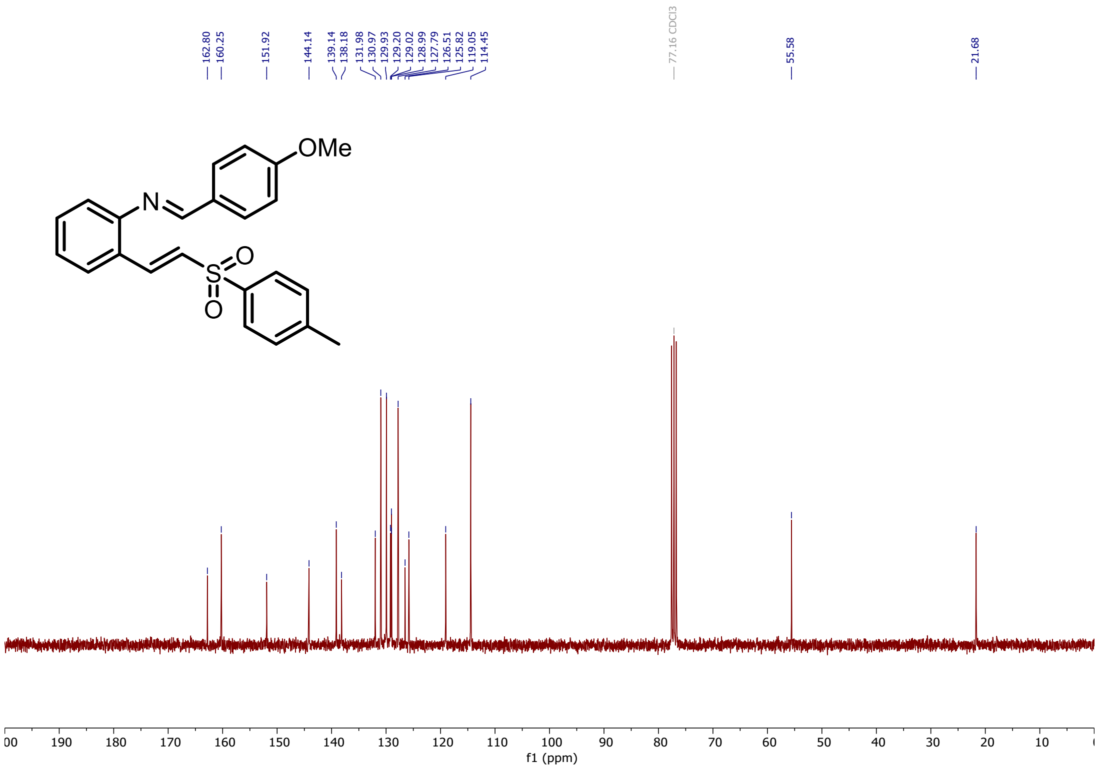

(*E*)-1-(4-Benzyloxy)phenyl)-*N*-(2-((*E*)-2-tosylvinyl)phenyl)methanimine (1h)

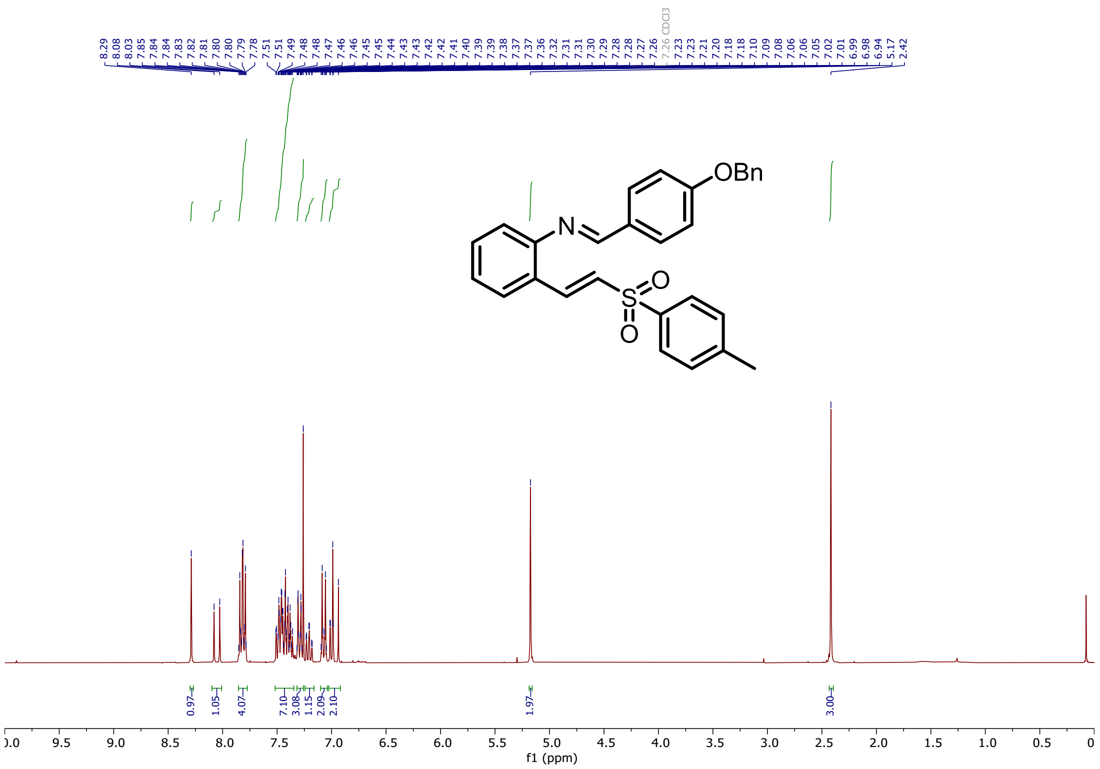

1h (^{13}C-NMR)

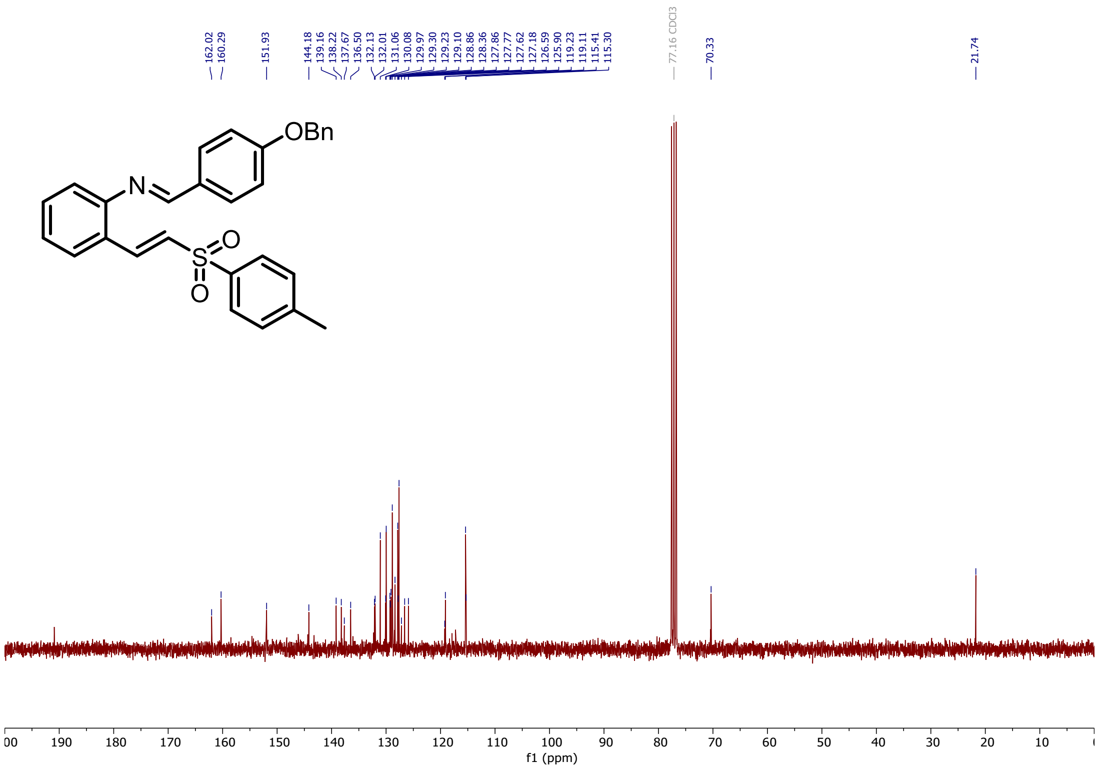

(*E*)-1-(4-Methylthio)phenyl)-*N*-(2-((*E*)-2-tosylvinyl)phenyl)methanimine (1i)

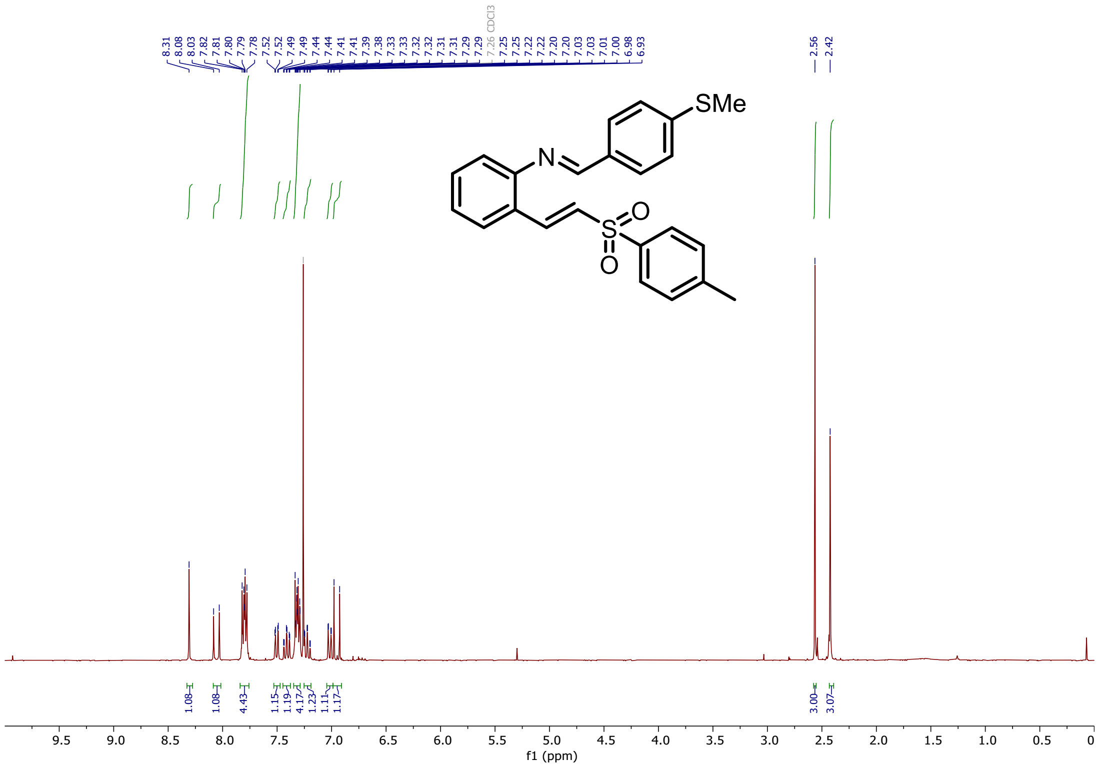

1i (^{13}C-NMR)

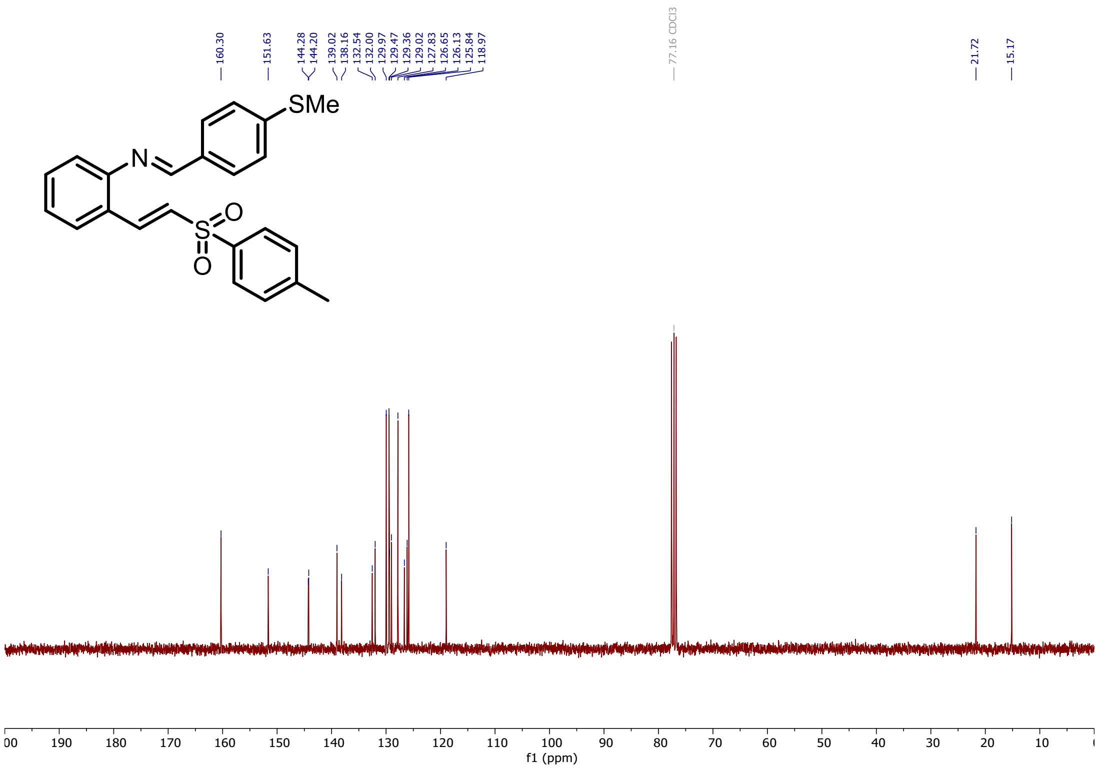

_N,N_-Dimethyl-4-((_E_)-((2-((_E_)-2-tosylvinyl)phenyl)imino)methyl)aniline (1j)

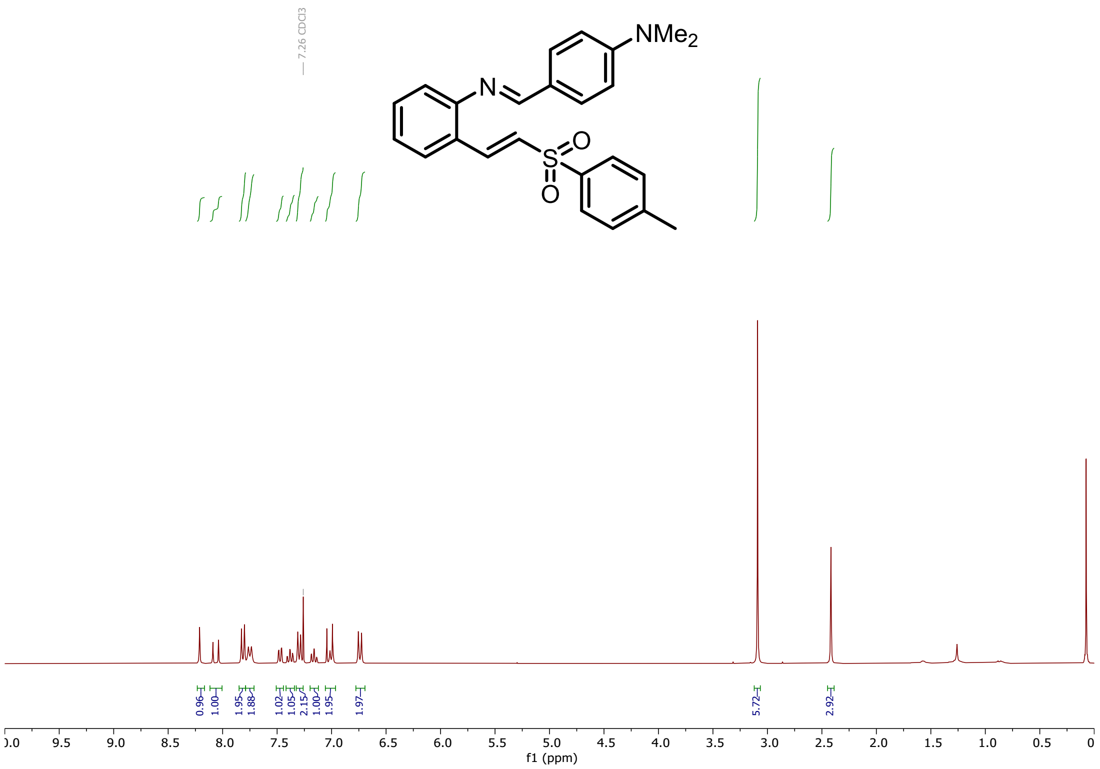

1j (^{13}C-NMR)

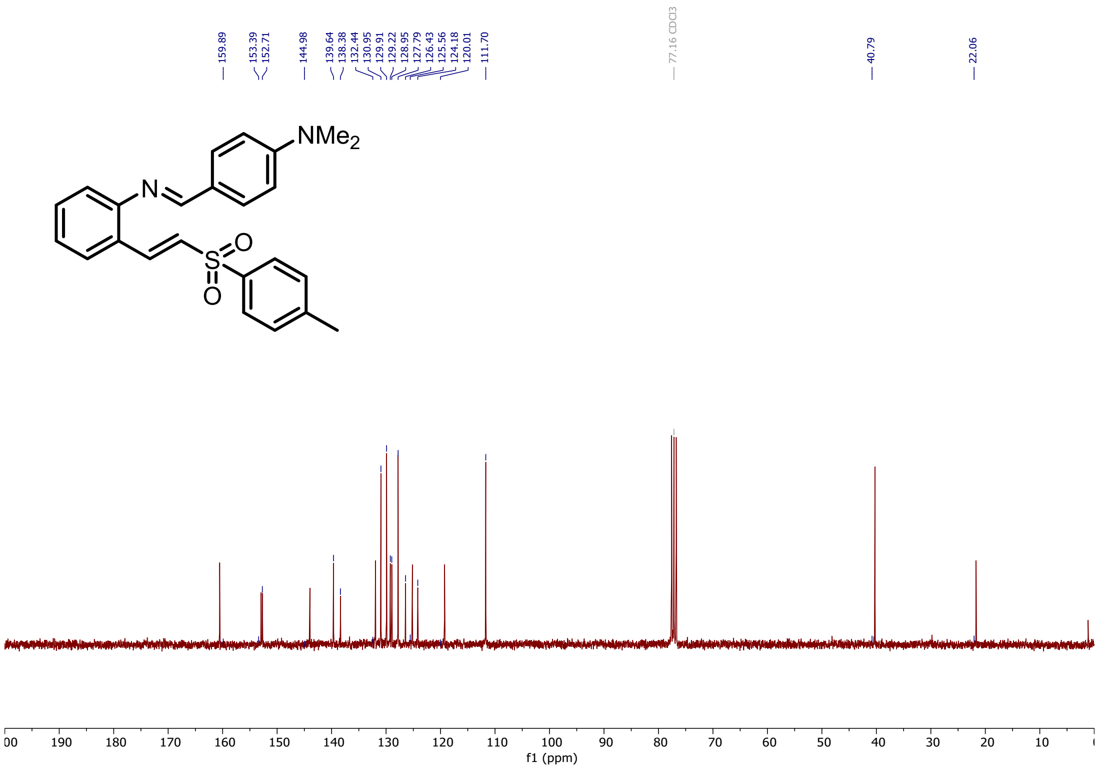

(*E*)-*N*-(2-((*E*)-2-tosylvinyl)phenyl)-1-(4-trifluoromethyl)phenyl)methanimine (1k)

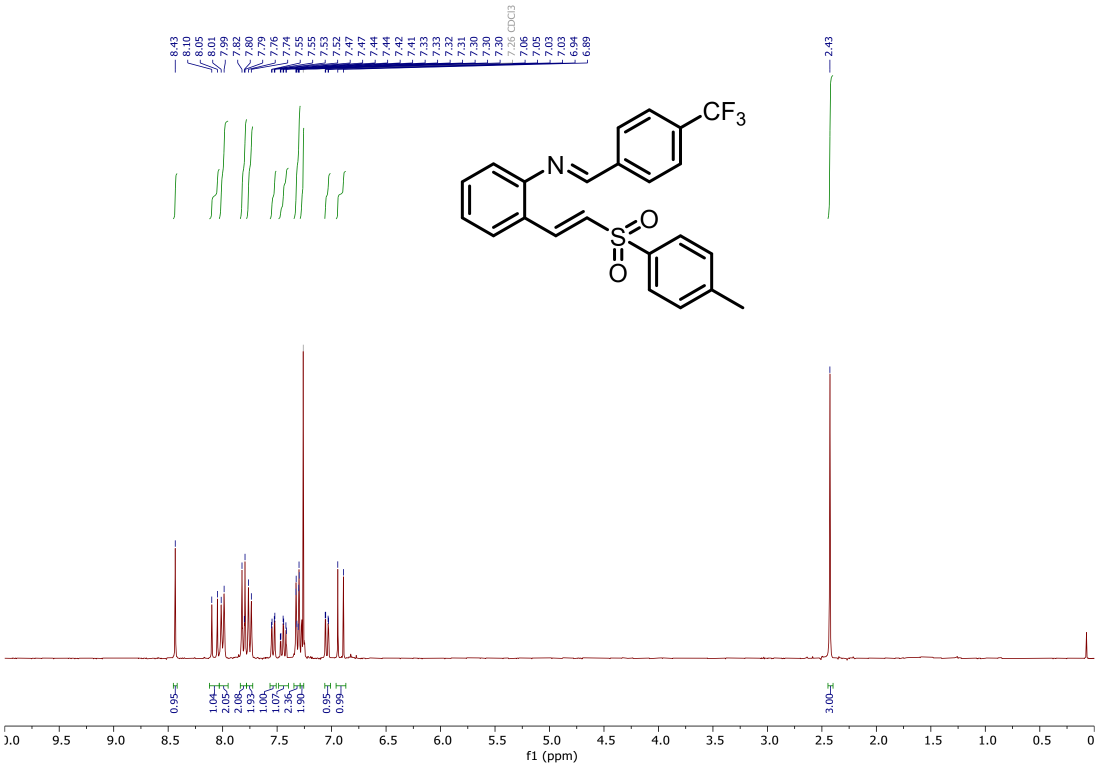

1k (^{13}C-NMR)

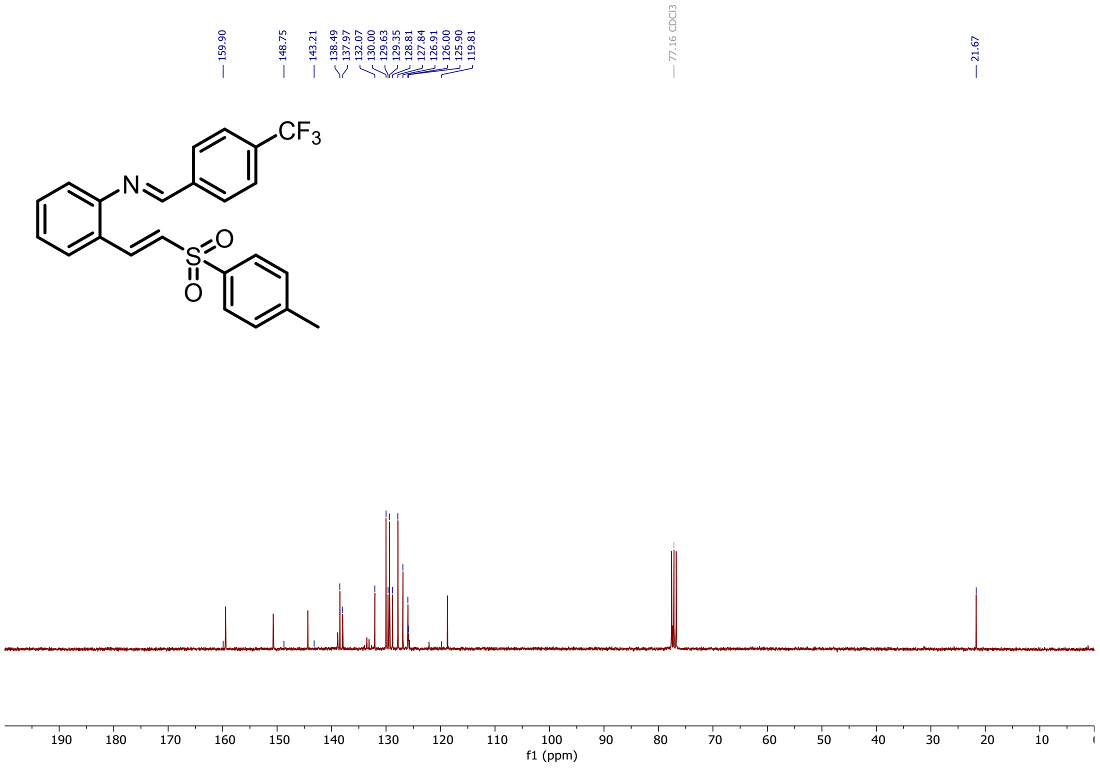

1k (^{19}F-NMR)

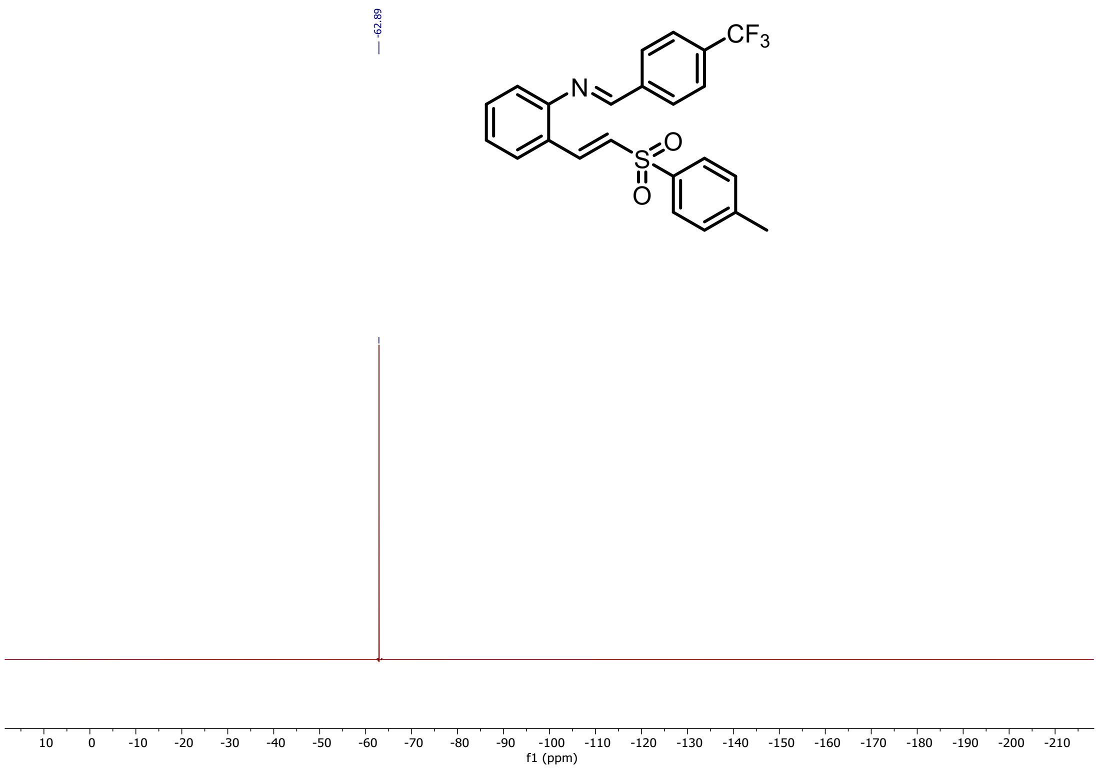

Methyl 4-((*E*)-((2-((*E*)-2-tosylvinyl)phenyl)imino)methyl)benzoate (1l)

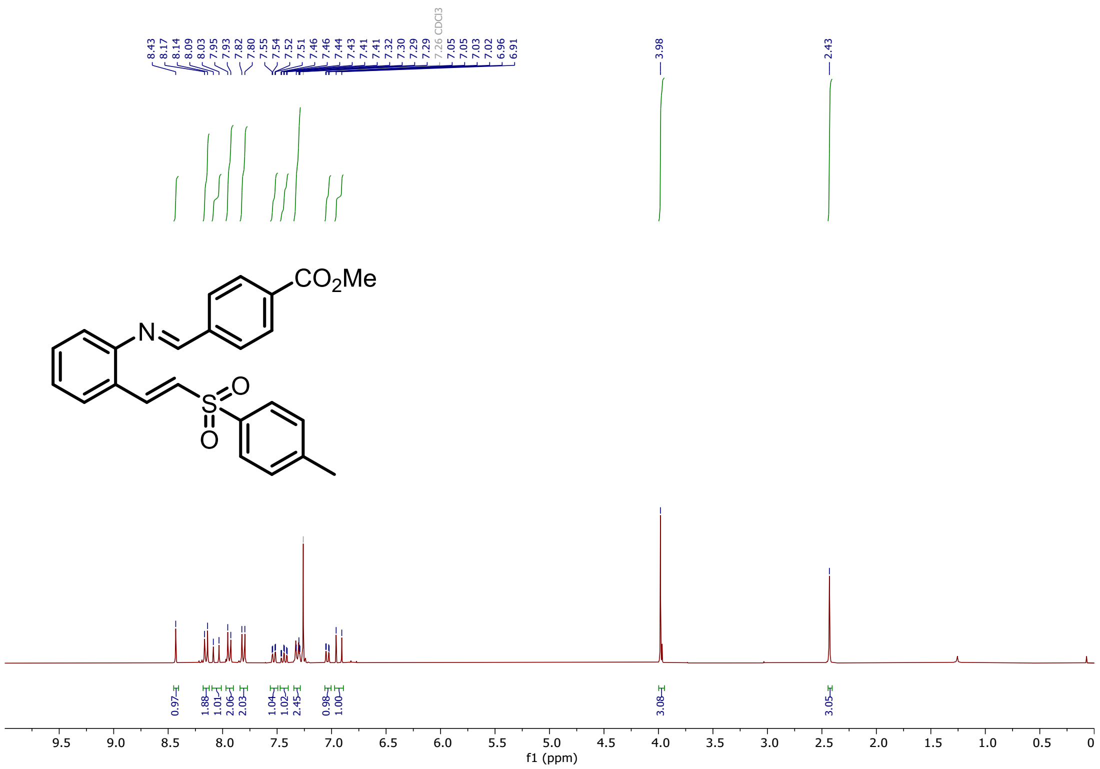

1l (^{13}C-NMR)

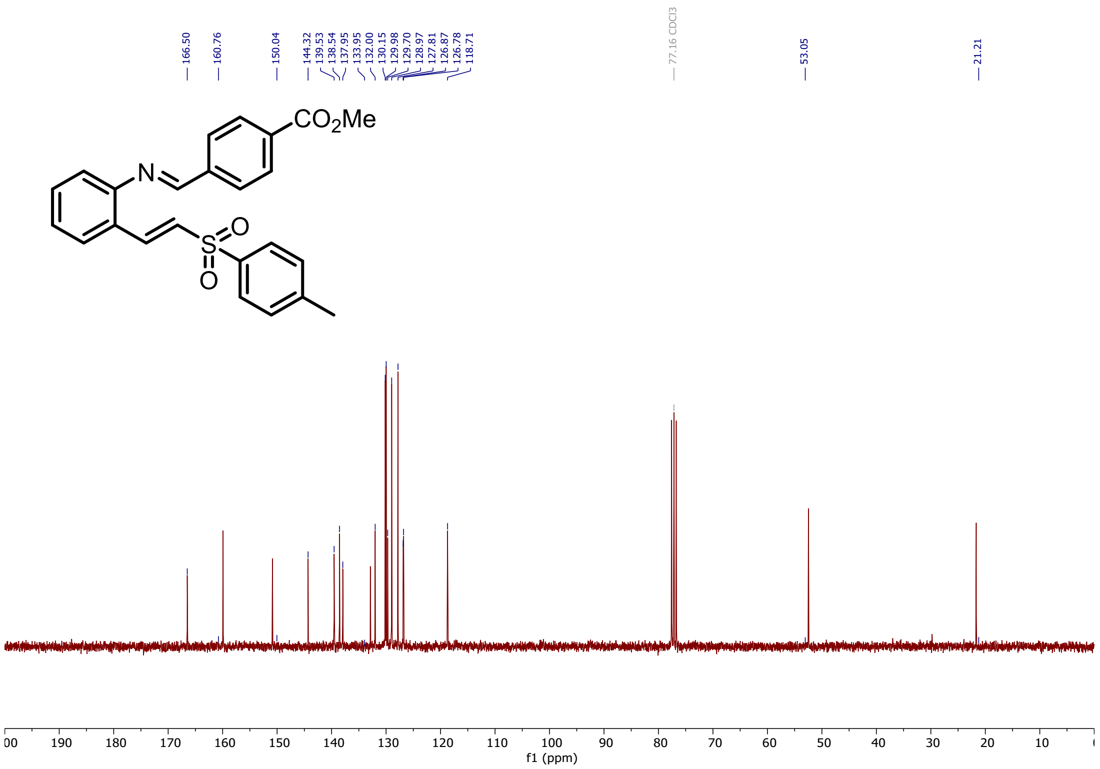

(*E*)-1-(4-Nitrophenyl)-*N*-(2-((*E*)-2-tosylvinyl)phenyl)methanimine (1m) (CH_2Cl_2 present in the spectrum, does not affect the yield).

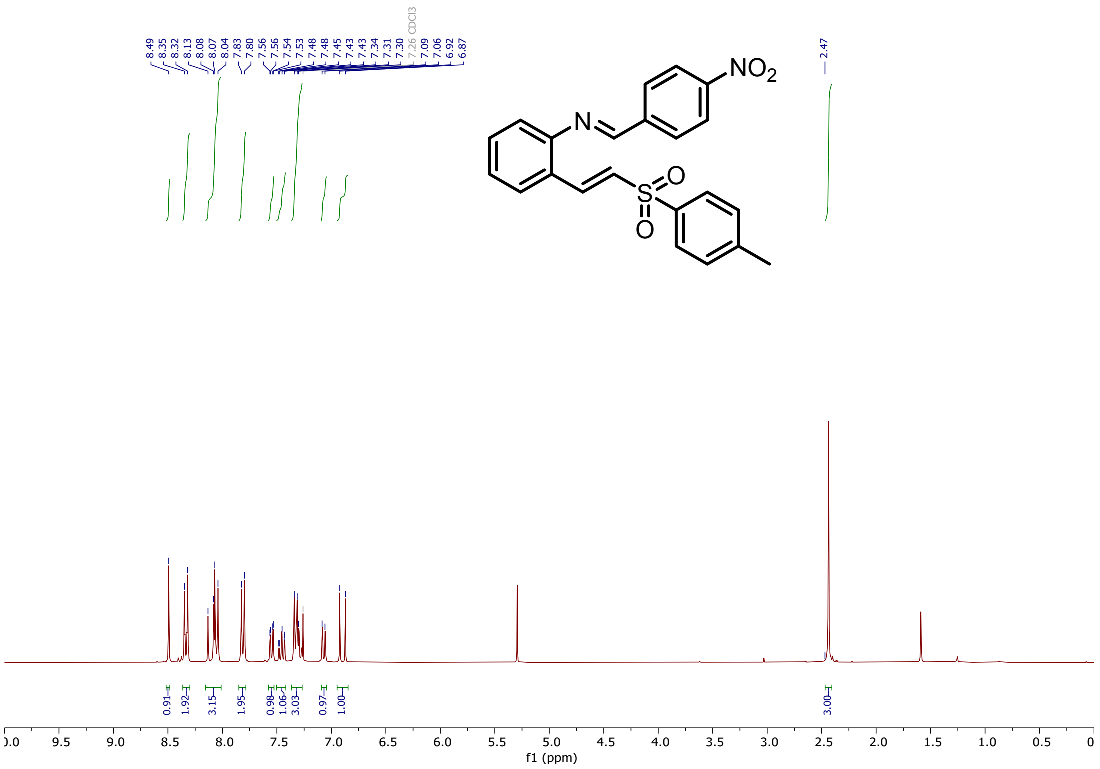

1m (^{13}C-NMR)

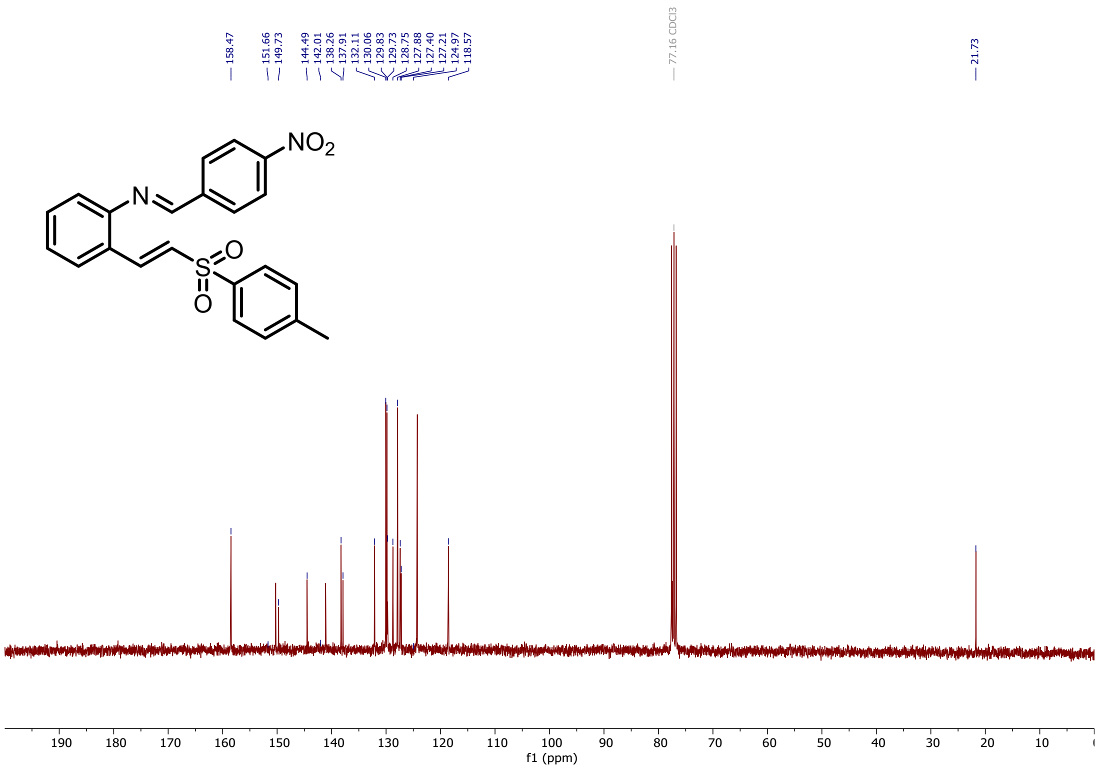

(*E*)-1-(Pyridin-4-yl)-*N*-(2-((*E*)-2-tosylvinyl)phenyl)methanimine (1n)

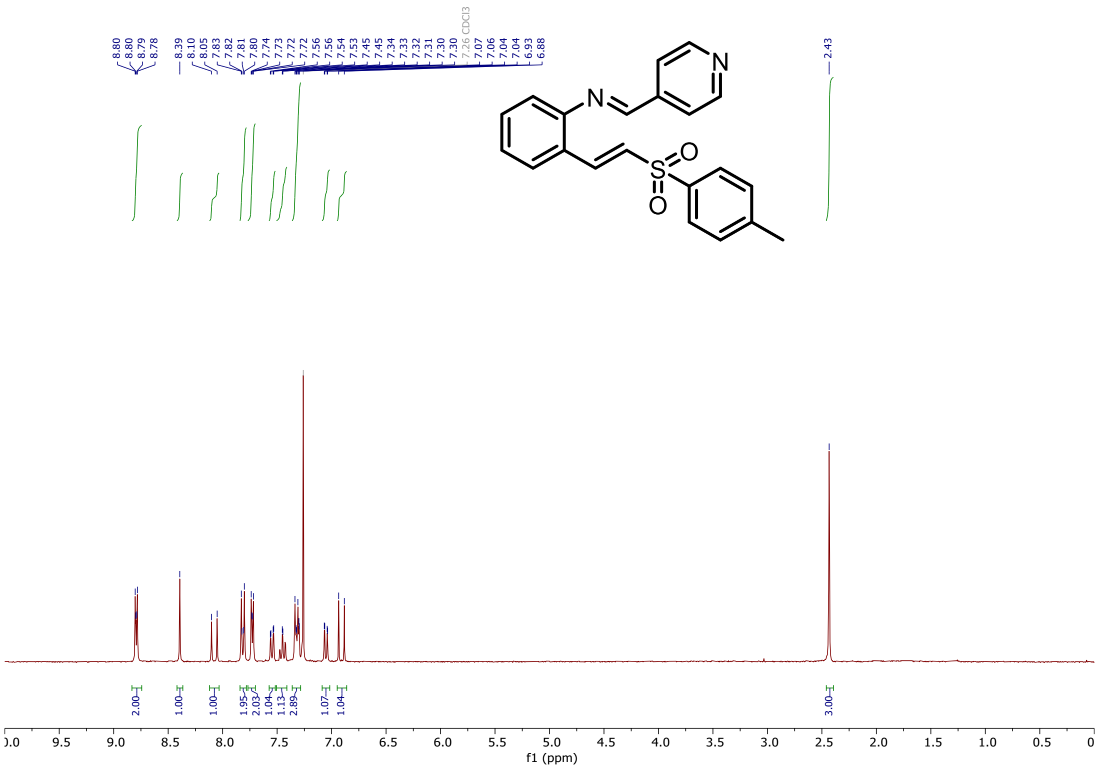

1n (^{13}C-NMR)

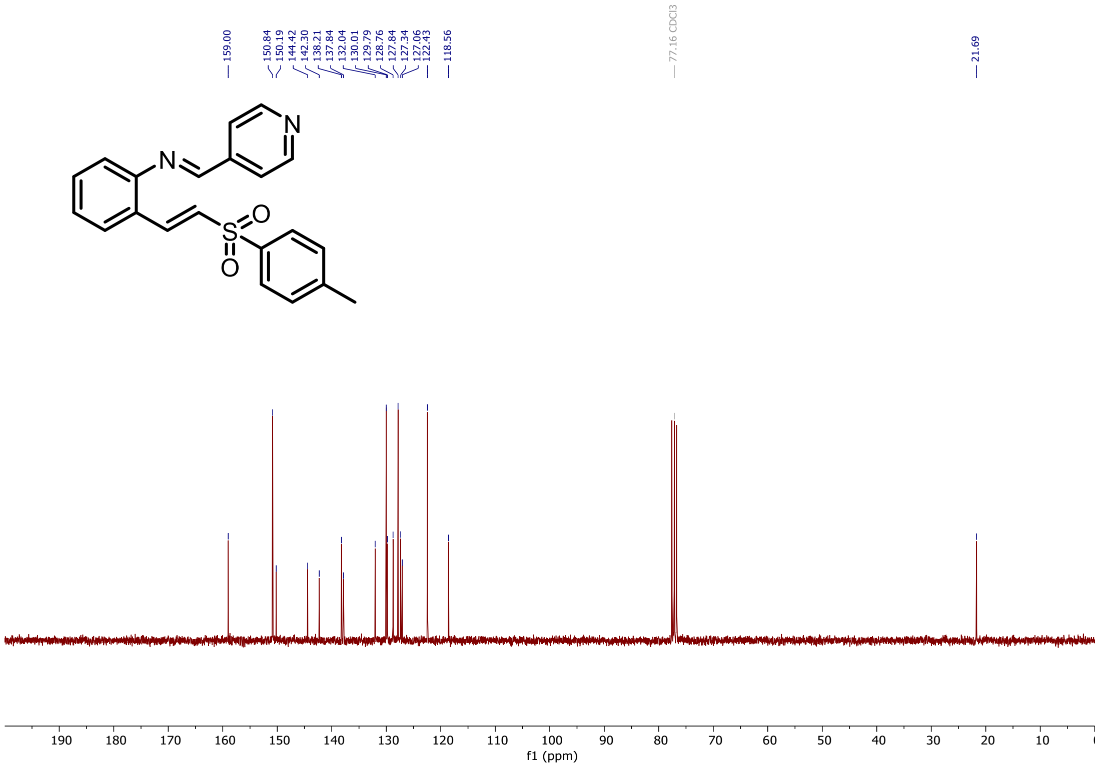

(*E*)-1-(Naphthalen-1-yl)-*N*-(2-((*E*)-2-tosylvinyl)phenyl)methanimine (1o)

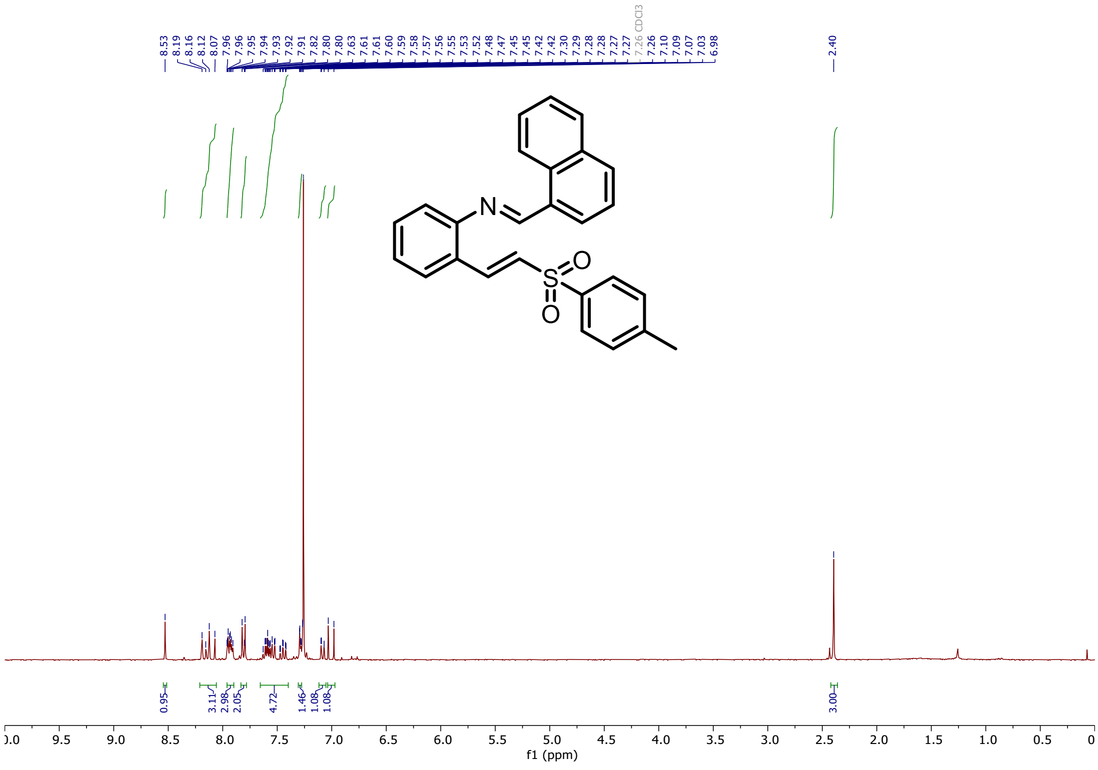

1o (^{13}C-NMR)

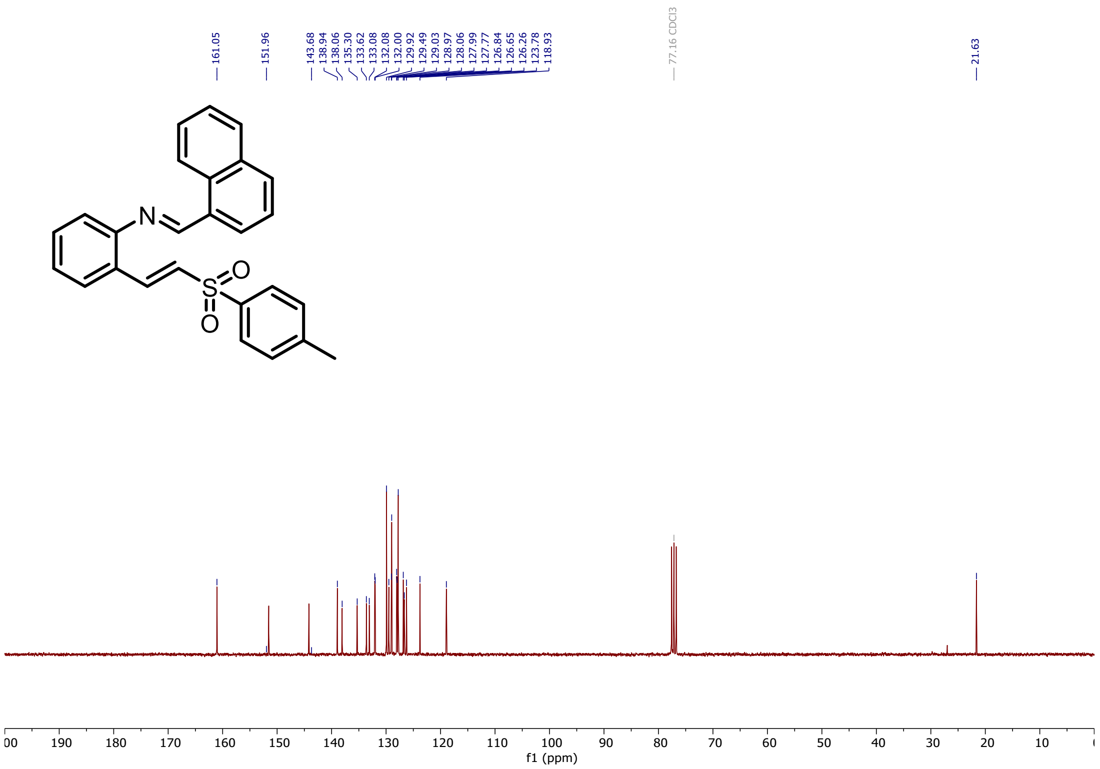

(*E*)-1-(Furan-2-yl)-*N*-(2-((*E*)-2-tosylvinyl)phenyl)methanimine (1p)

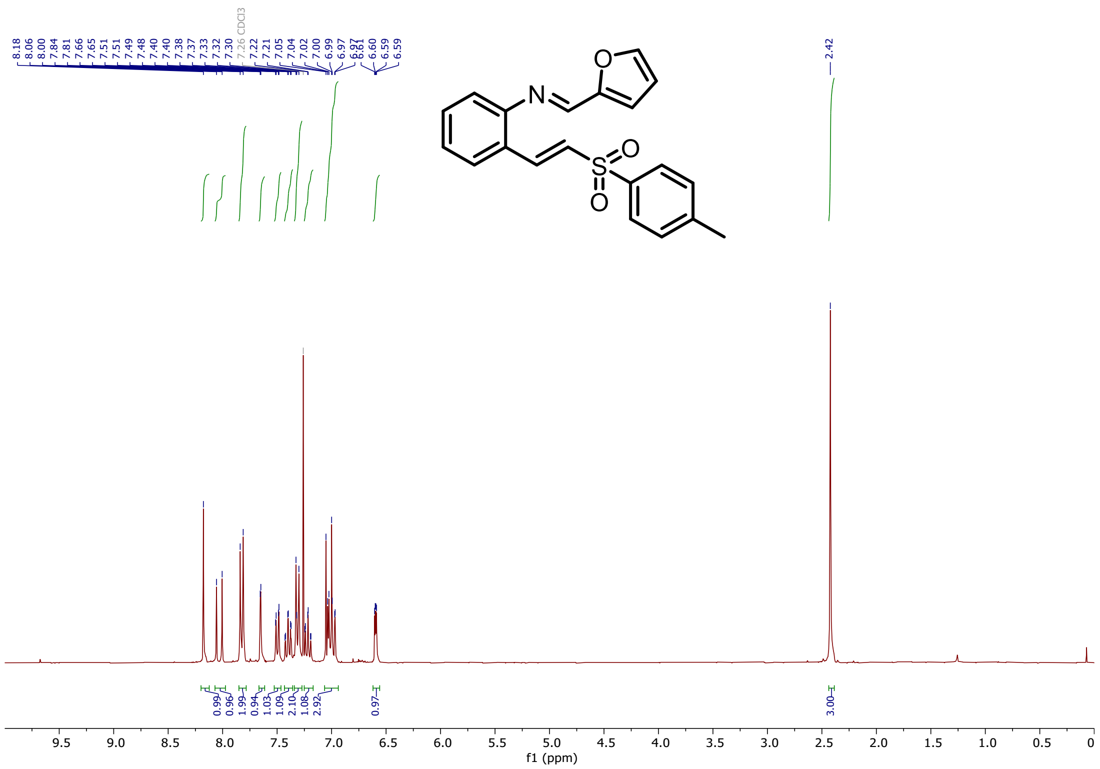

1p (^{13}C-NMR)

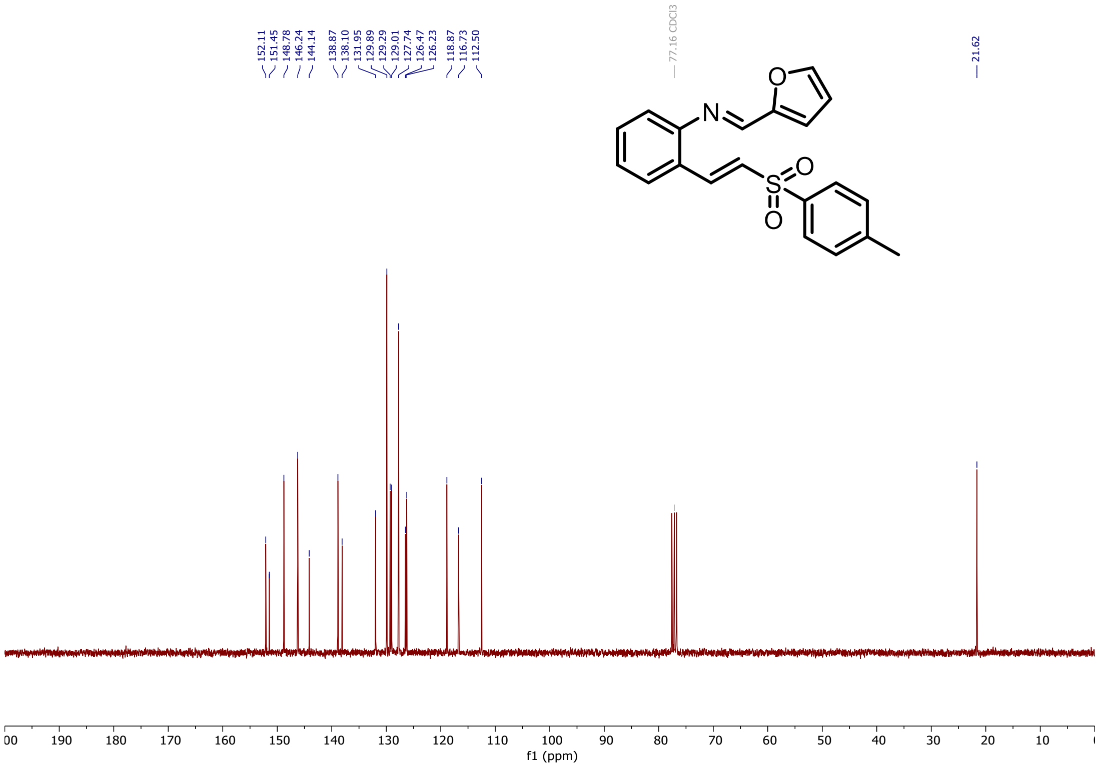

(*E*)-1-(Thiophen-2-yl)-*N*-(2-((*E*)-2-tosylvinyl)phenyl)methanimine (1q)

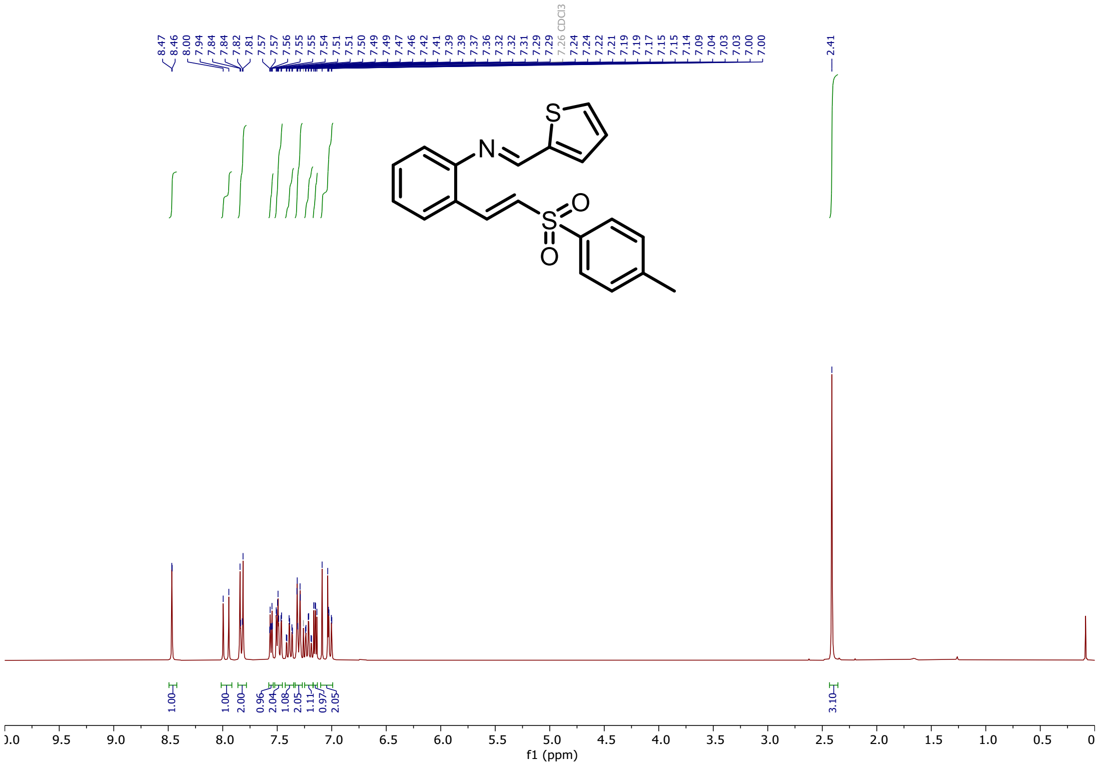

1q (^{13}C-NMR)

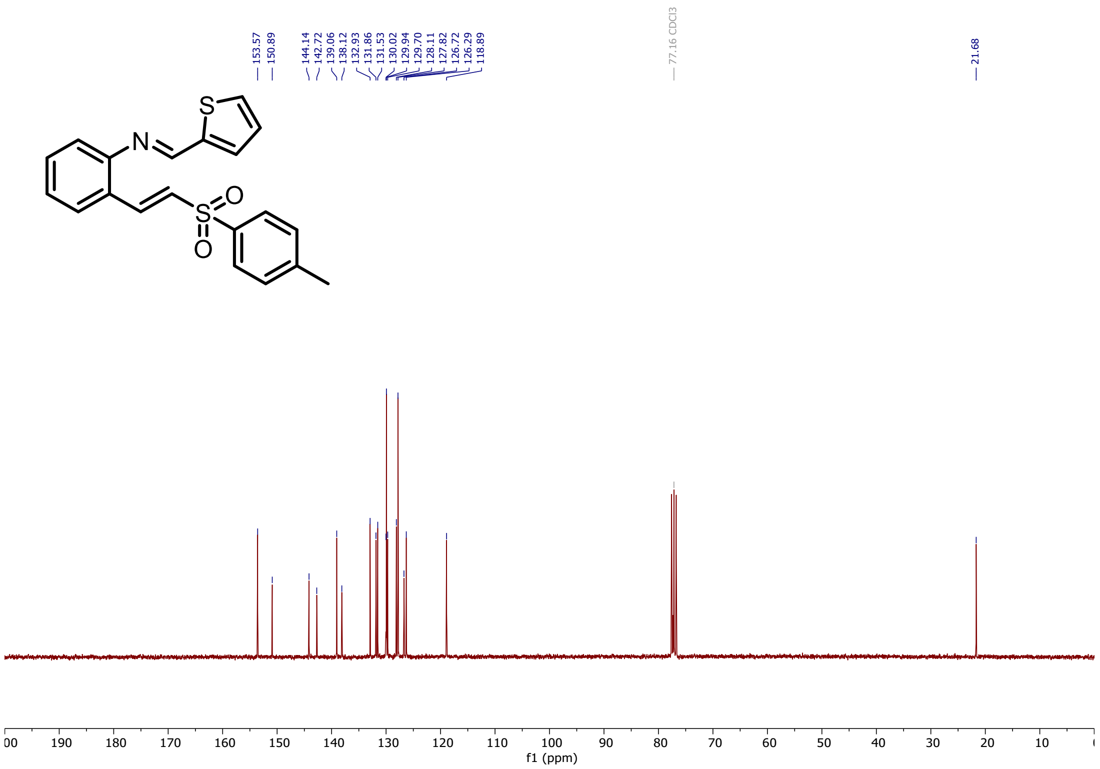

(E)-*N*-(4-Methoxy-2-((*E*)-2-tosylvinyl)phenyl)-1-phenylmethanimine (1r)

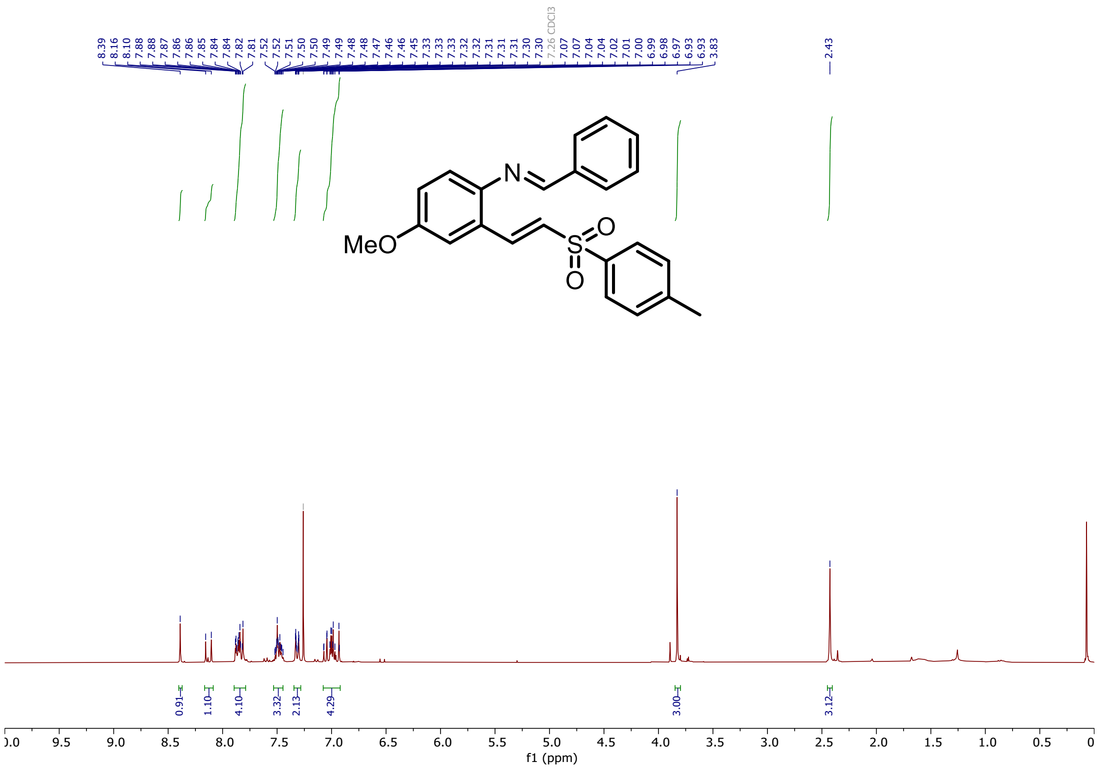

1r (^{13}C-NMR)

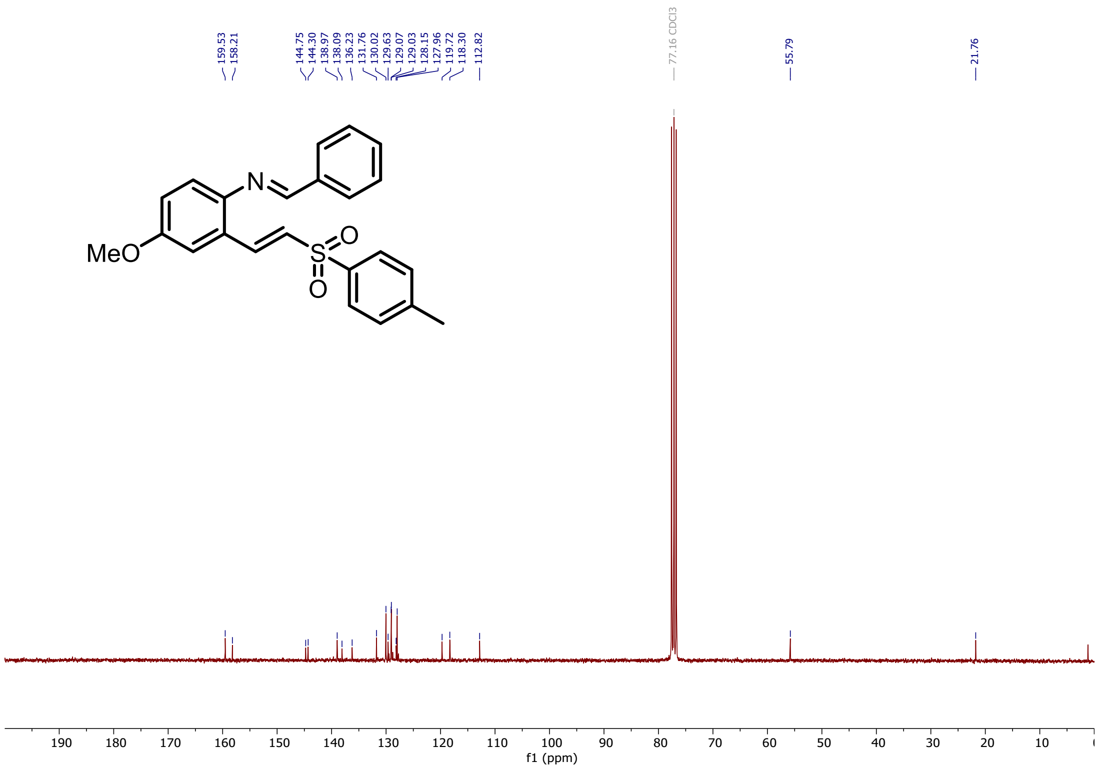

(*E*)-*N*-(4-Chloro-2-((*E*)-2-tosylvinyl)phenyl)-1-phenylmethanimine (1s)

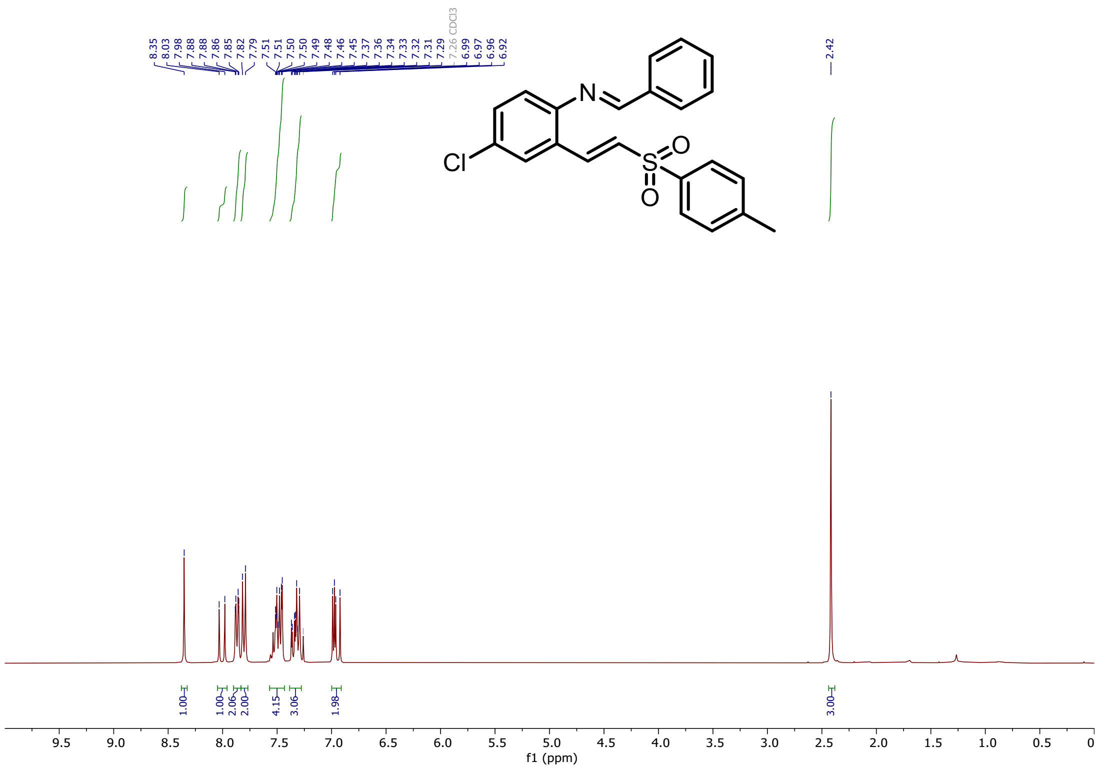

1s (^{13}C-NMR)

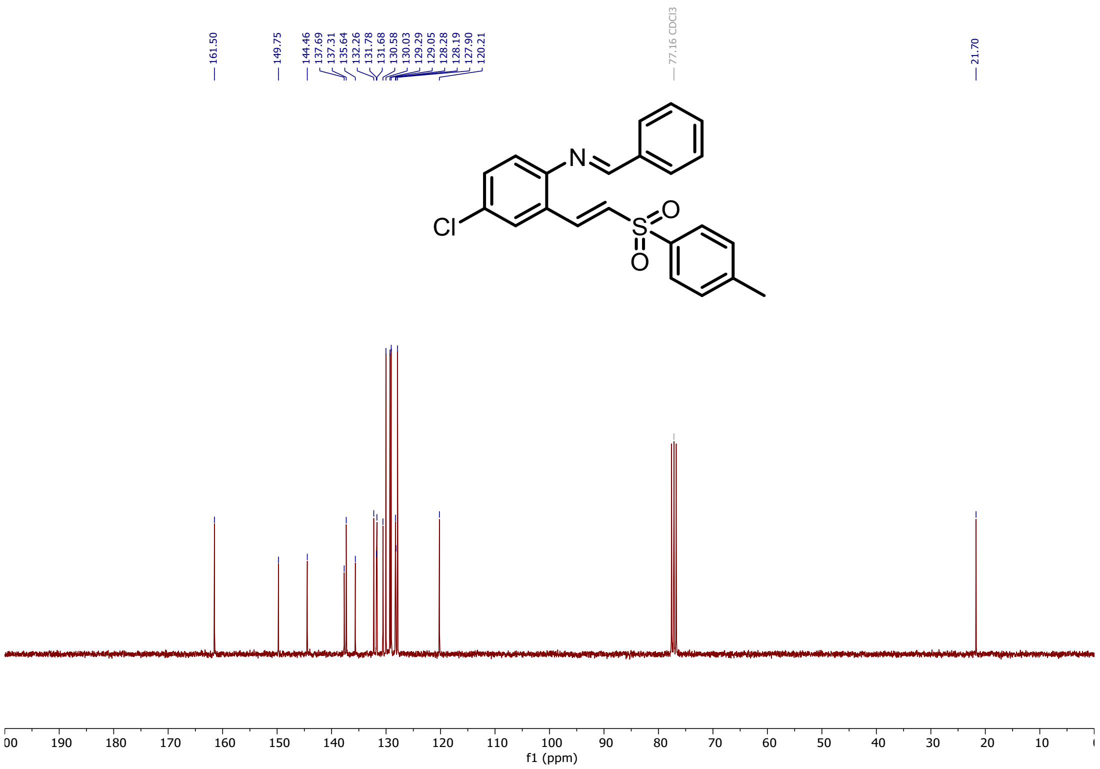

(*E*)-*N*-(5-Bromo-2-((*E*)-tosylvinyl)phenyl)-1-phenylmethanimine (1t) Not purified, used in situ.

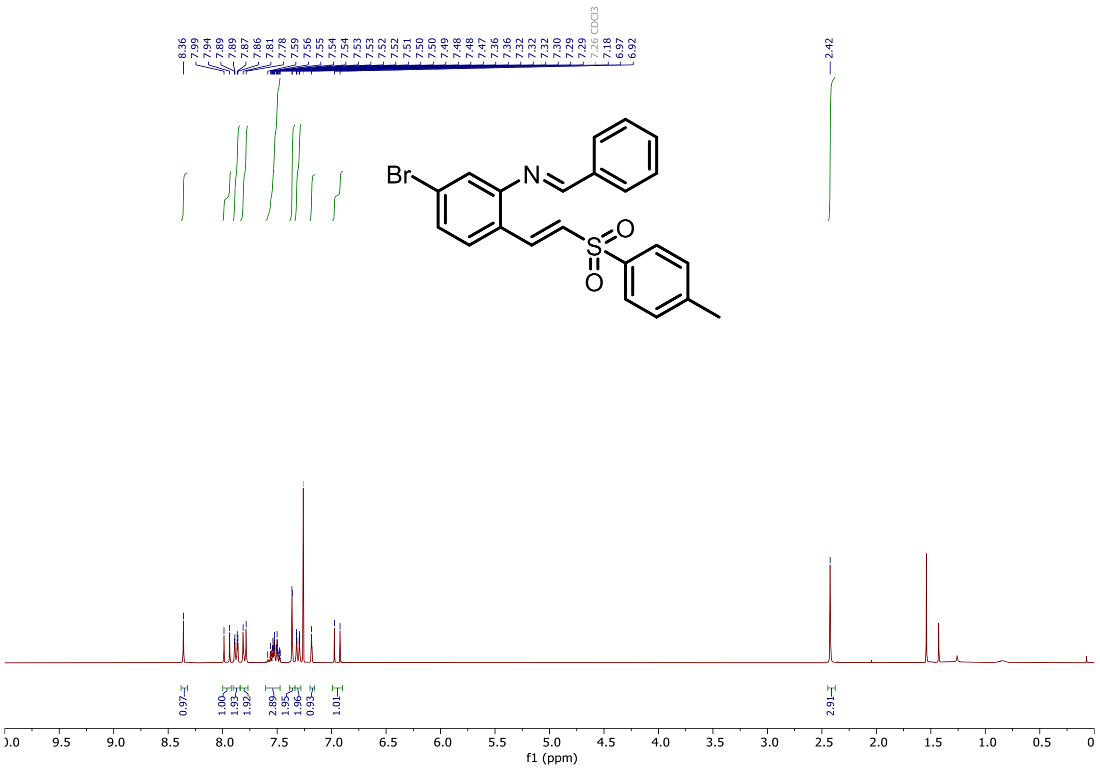

1t (^{13}C-NMR)

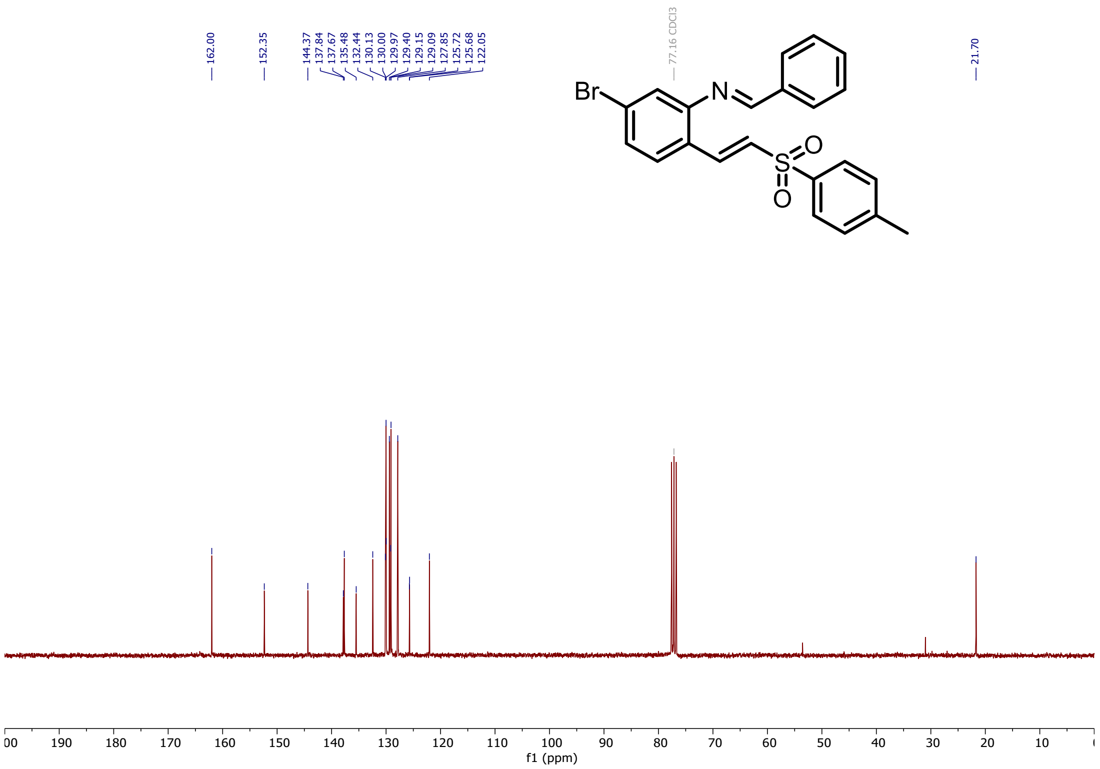

(*E*)-1-Phenyl-*N*-(6-((*E*)-2-tosylvinyl)benzo[*d*][1,3]dioxol-5-yl)methanimine (1u)

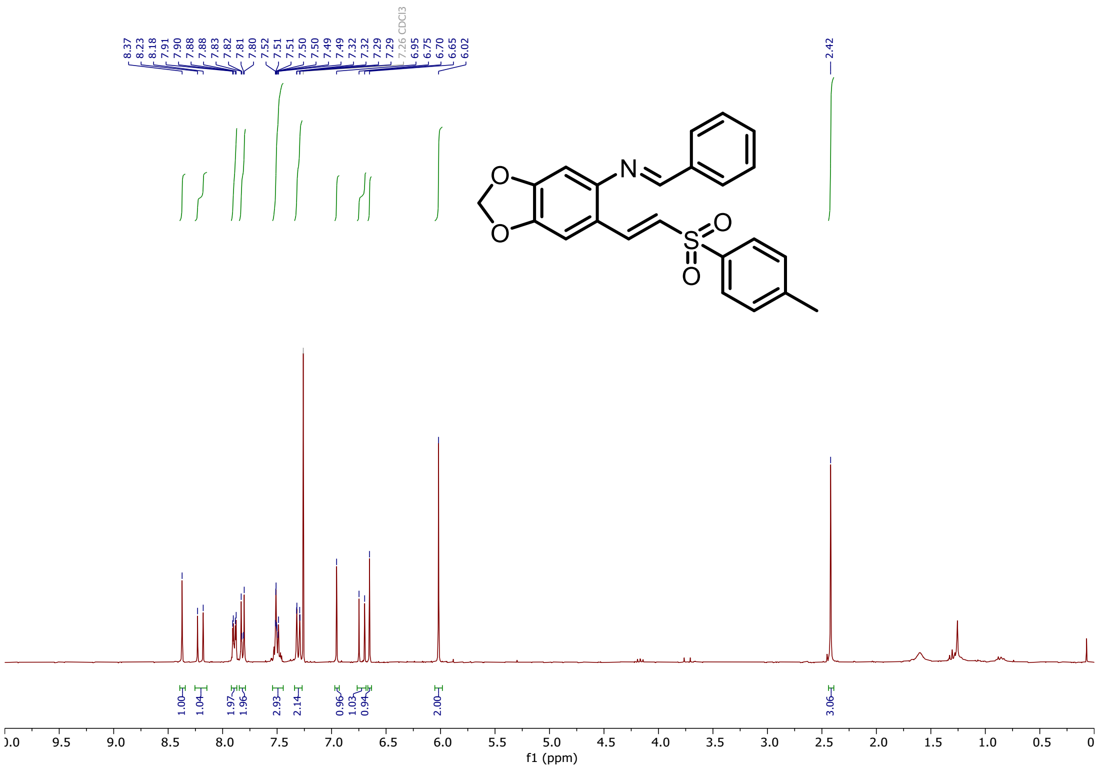

1u (13C-NMR)

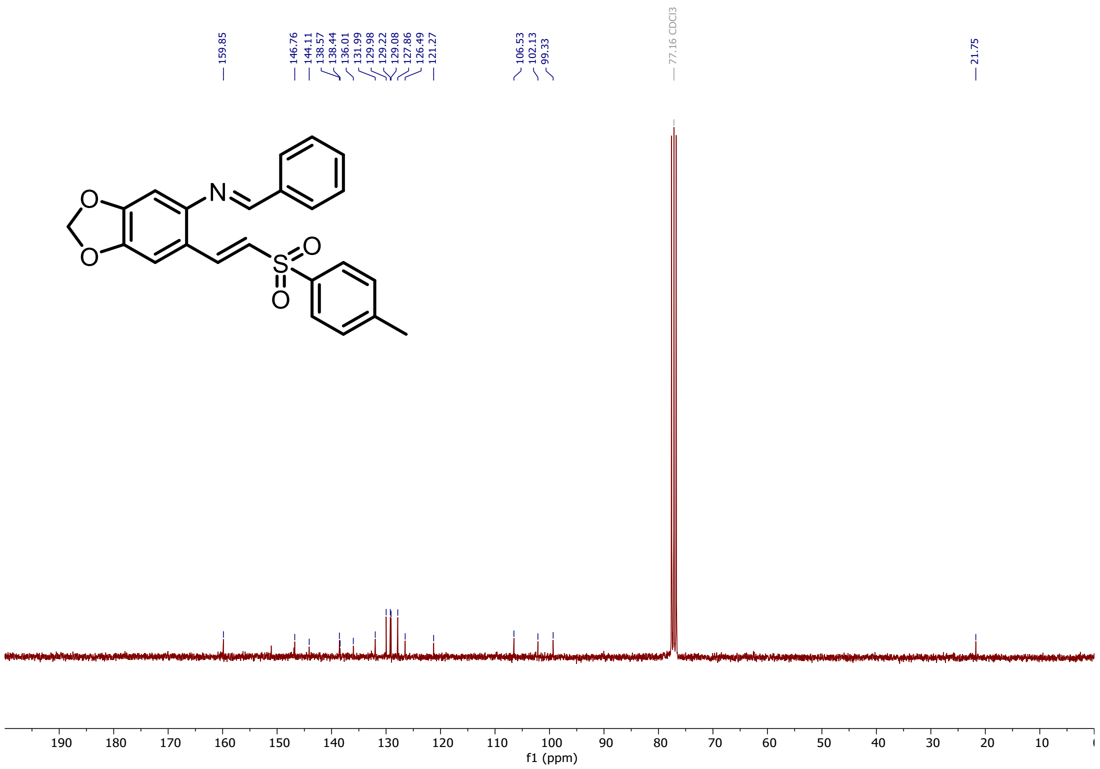

(*E*)-1-Cyclopropyl-*N*-(2-((*E*)-2-tosylvinyl)phenyl)methanimine (1v) Volatile imine, used without evaporation (cyclohexane is present in the spectrum).

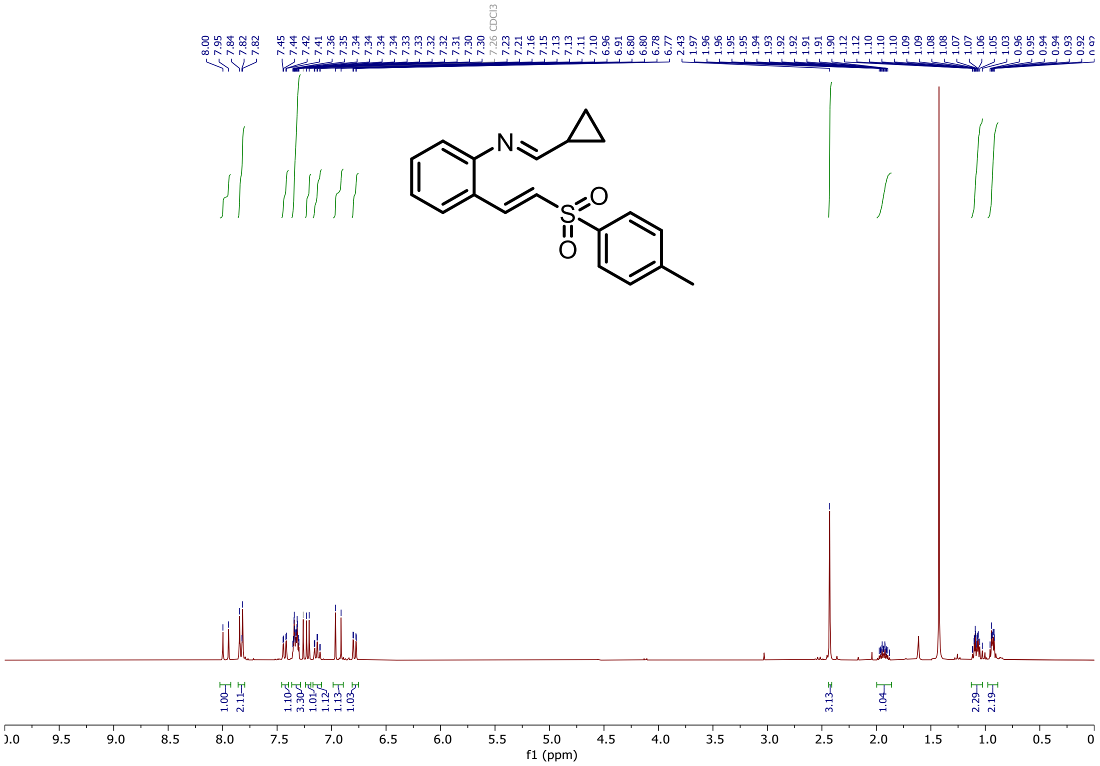

1v (^{13}C-NMR)

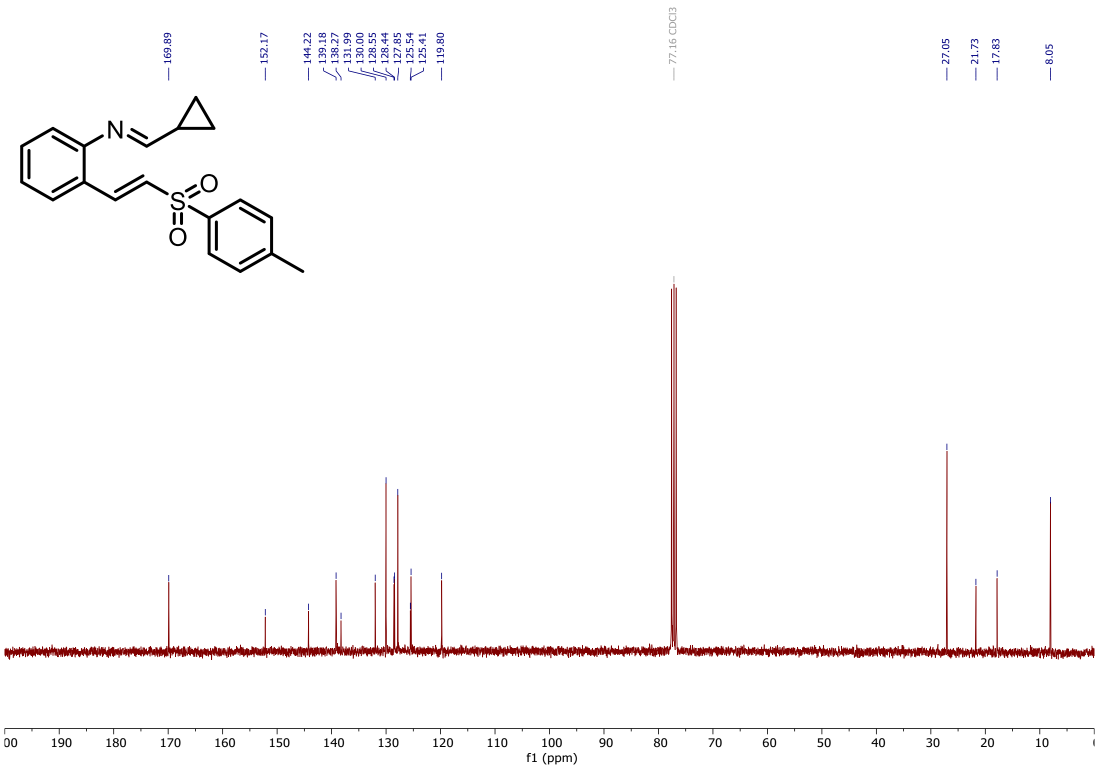

(*E*)-2,2-Dimethyl-*N*-(2-((*E*)-2-tosylvinyl)phenyl)propan-1-imine (1w)

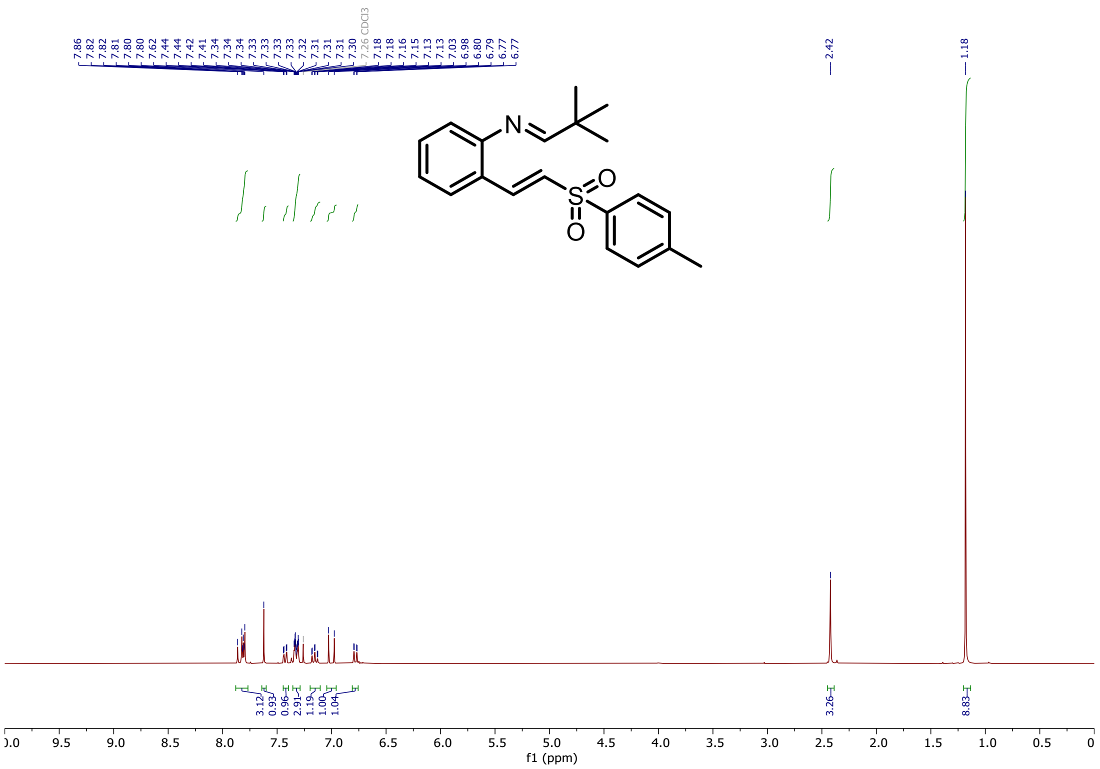

1w (^{13}C-NMR)

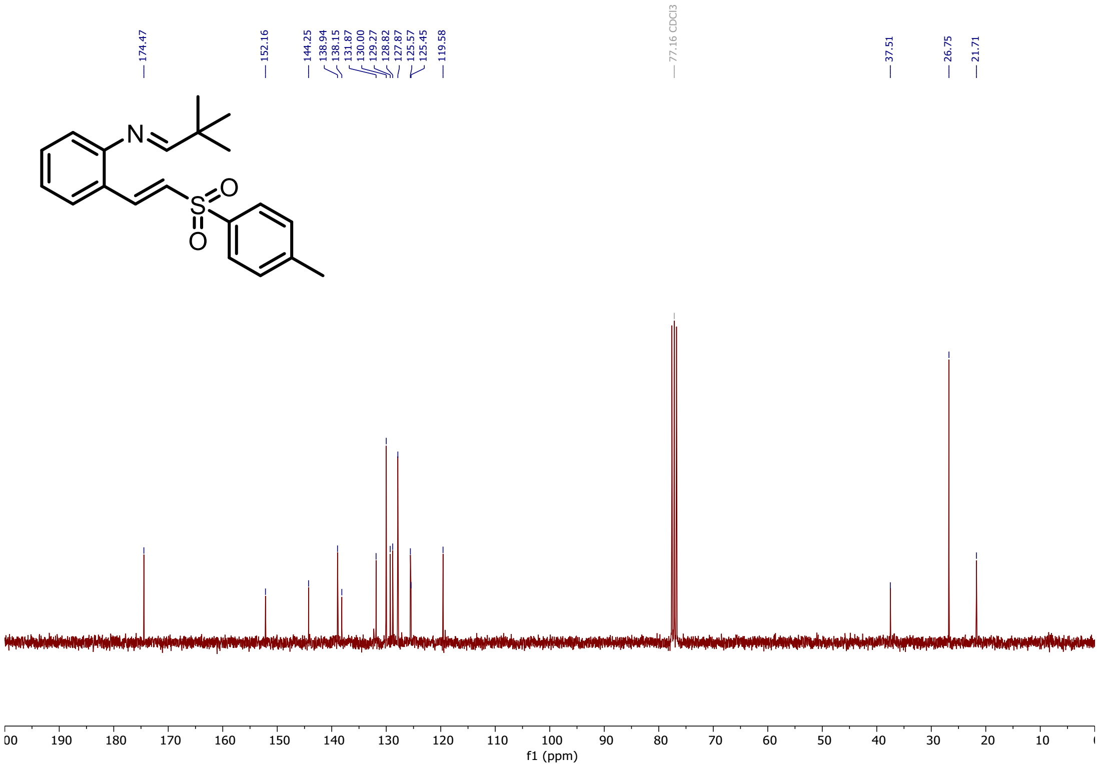

2-Phenylquinoline (2a)

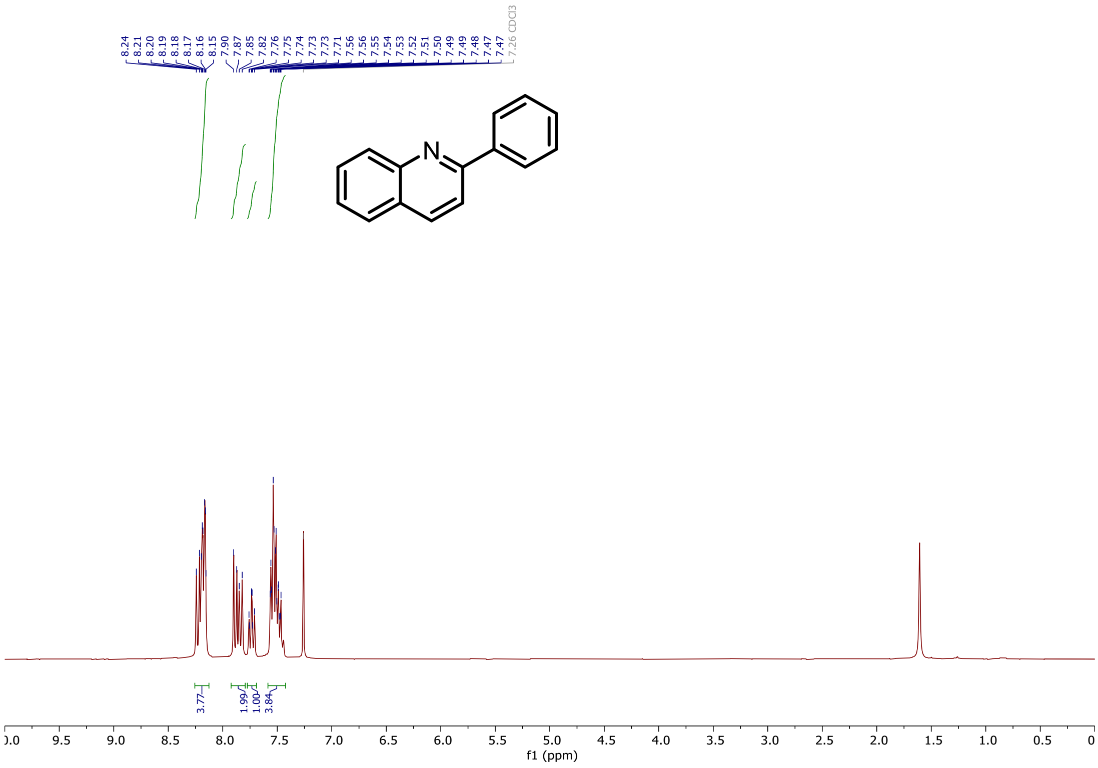

2-(*o*-Tolyl)quinoline (2b)

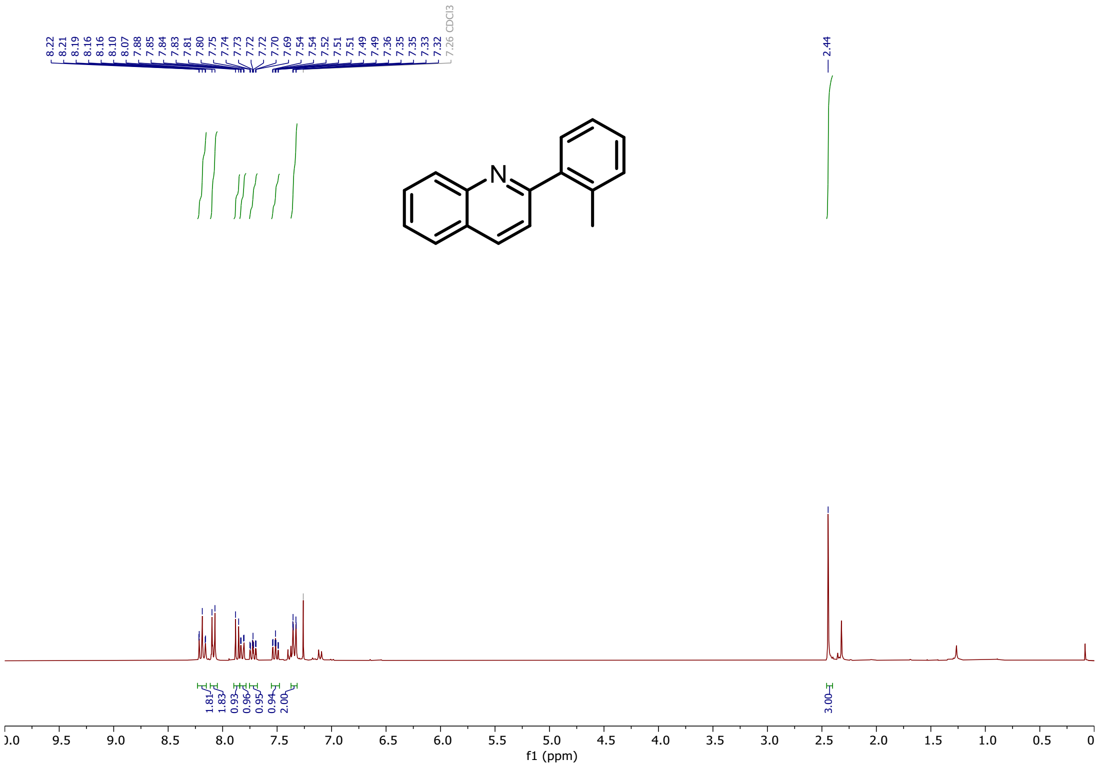

2-(4-Fluorophenyl)quinoline (2c)

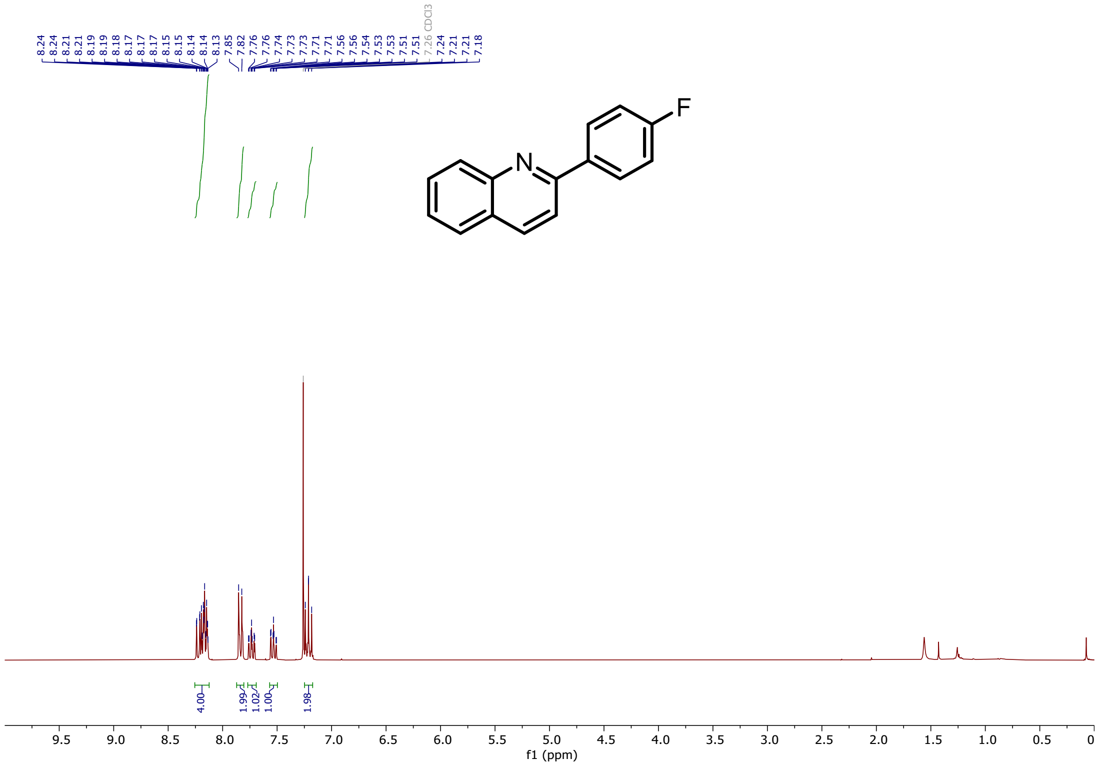

2-(4-Chlorophenyl)quinoline (2d)

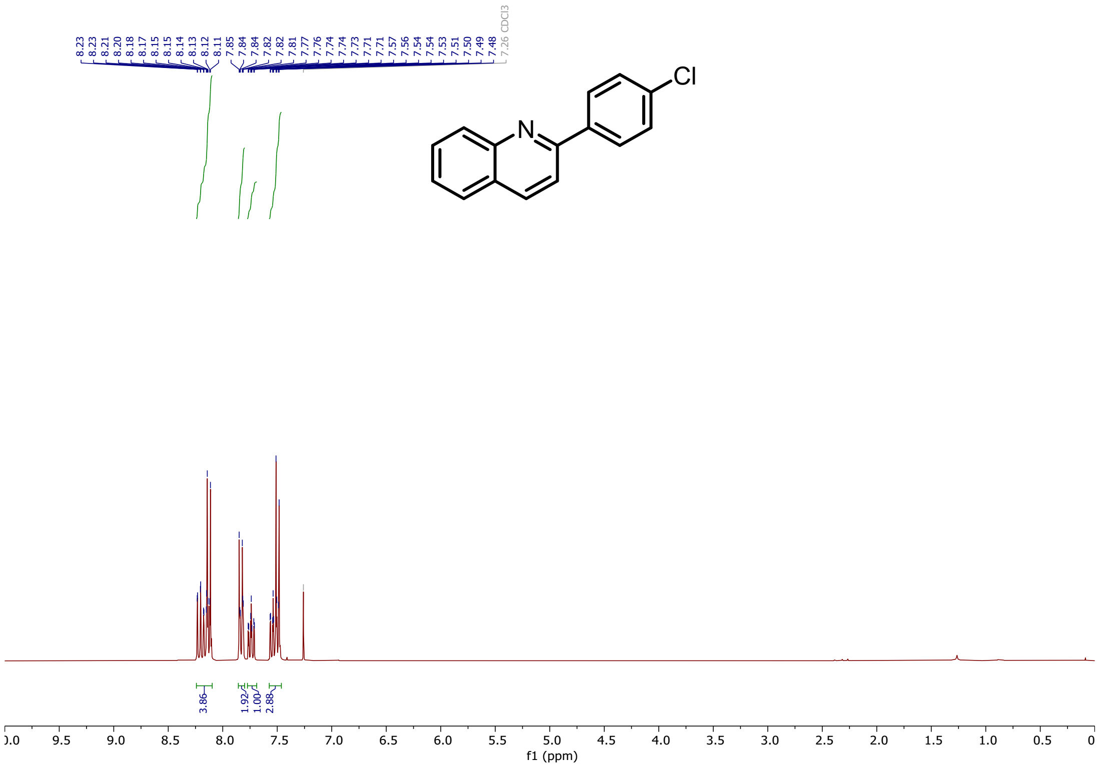

2-(4-Bromophenyl)quinoline (2e)

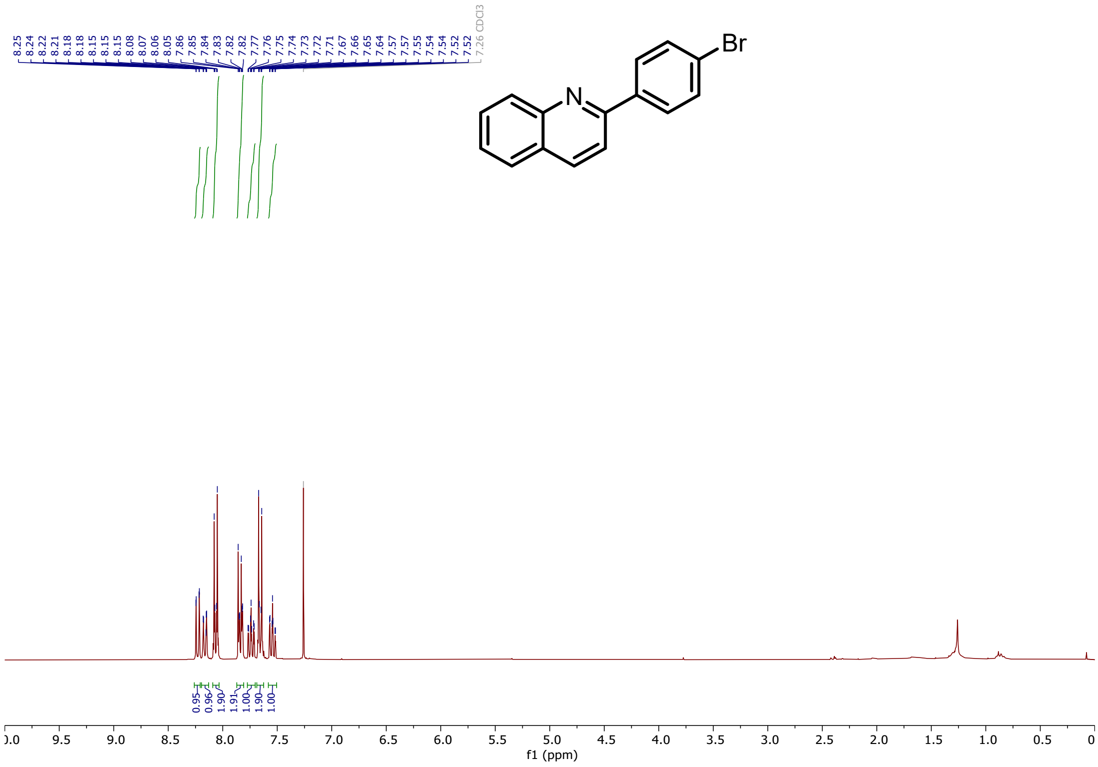

2-(*p*-Tolyl)quinoline (2f) Contaminated with *p*-tolyl disulfide in 3/0.81 ratio due to impossible separation (yield is determined assuming this value)

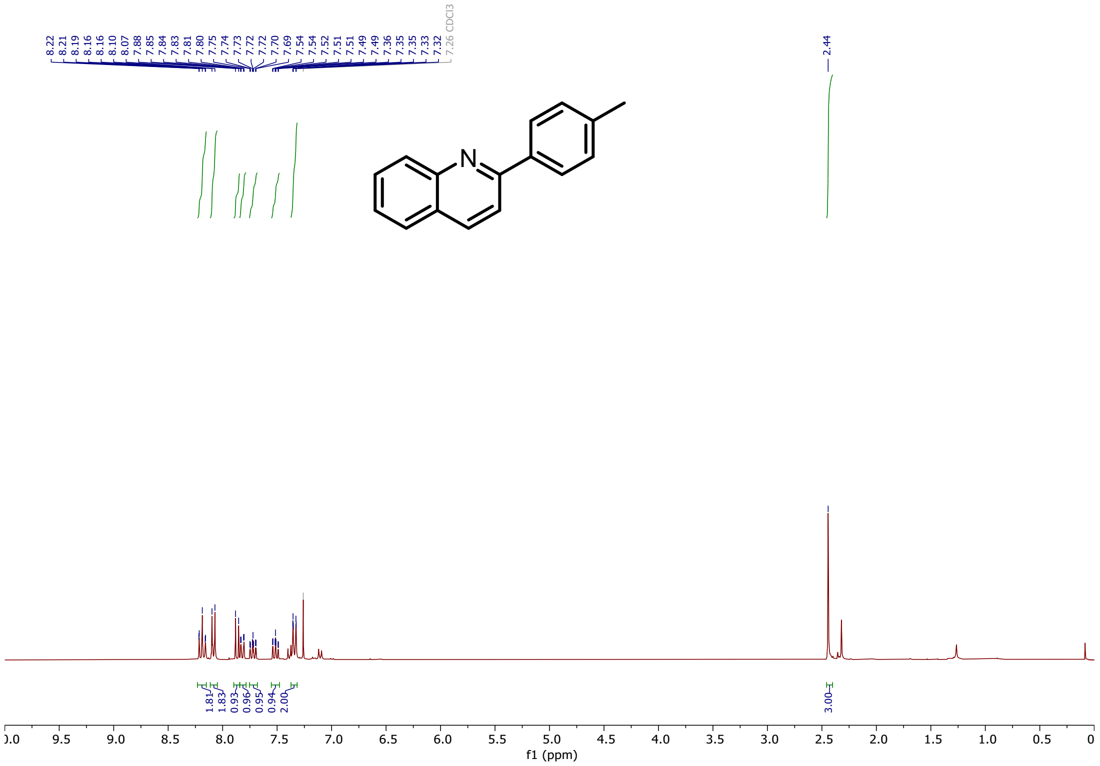

2-(4-Methoxyphenyl)quinoline (2g)

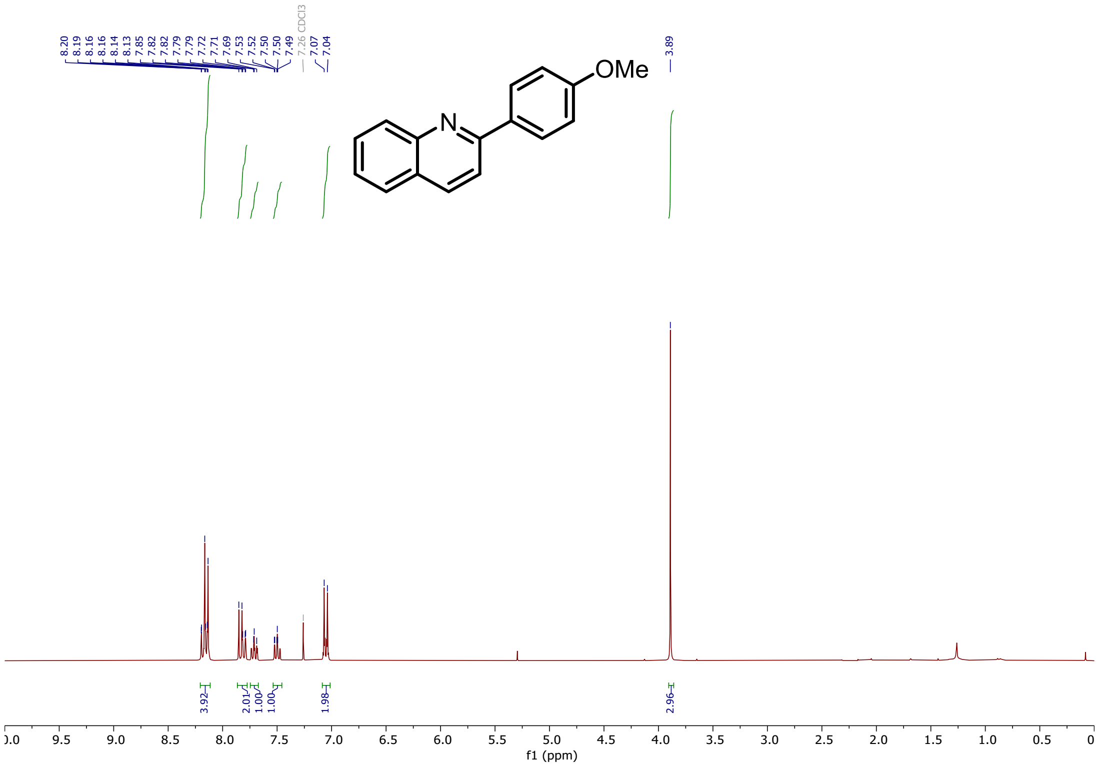

2-(4-(Benzyloxy)phenyl)quinoline (2h)

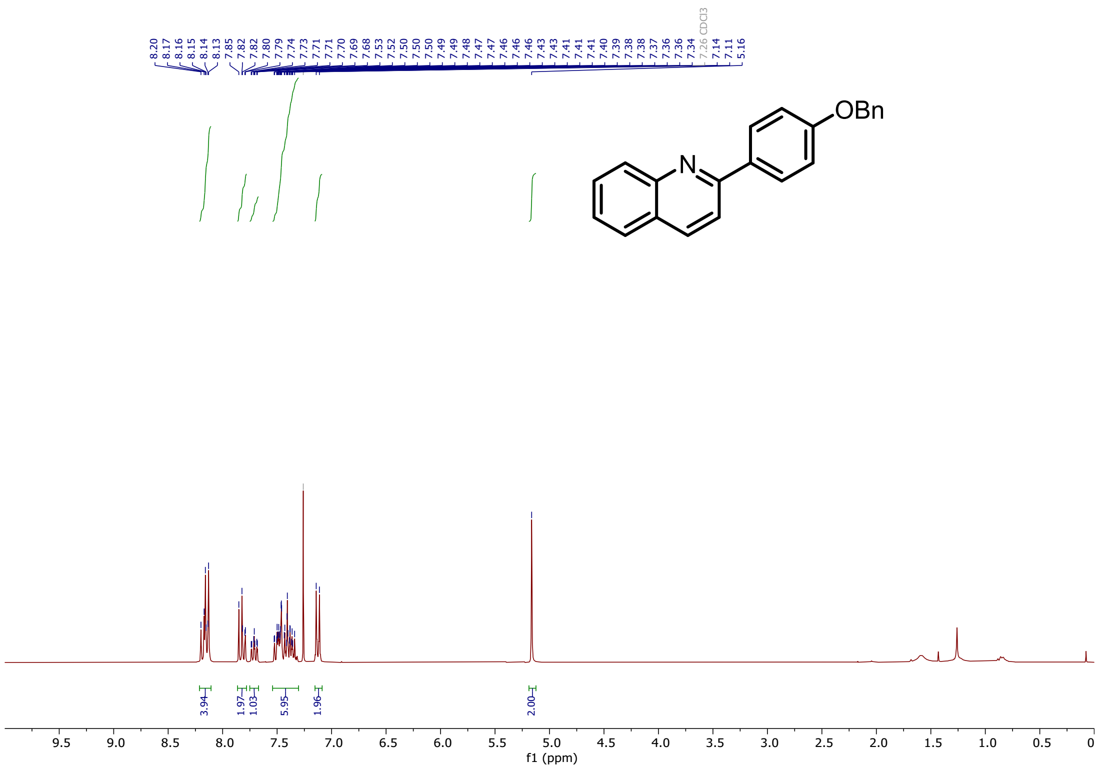

2-(4-(Methylthio)phenyl)quinoline (2i)

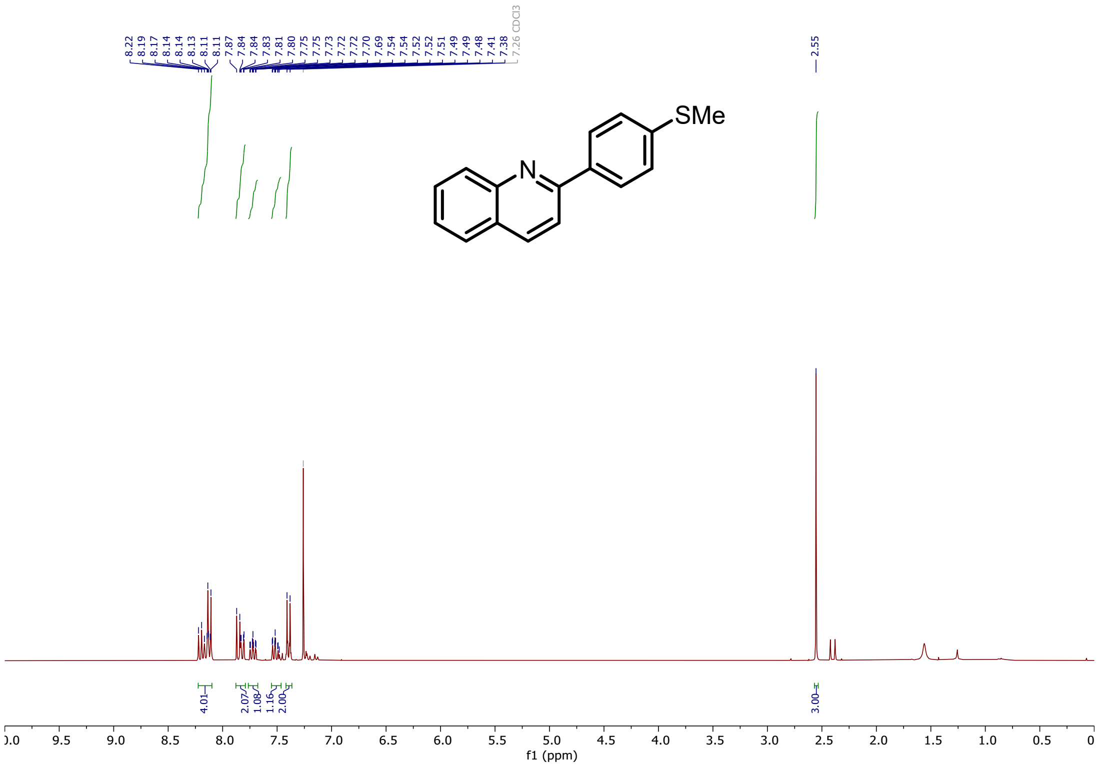

***N*, *N*-Dimethyl-4-(quinoline-2-yl)aniline (2j)**

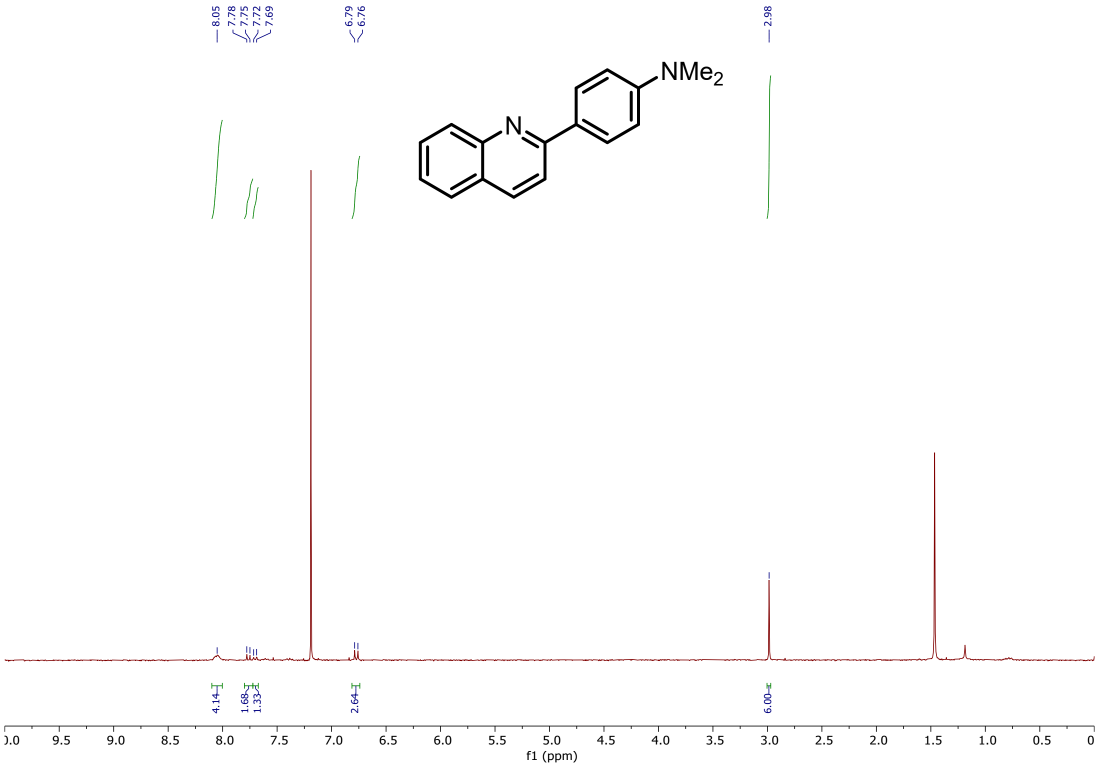

2-(4-(Trifluoromethyl)phenyl)quinoline (2k)

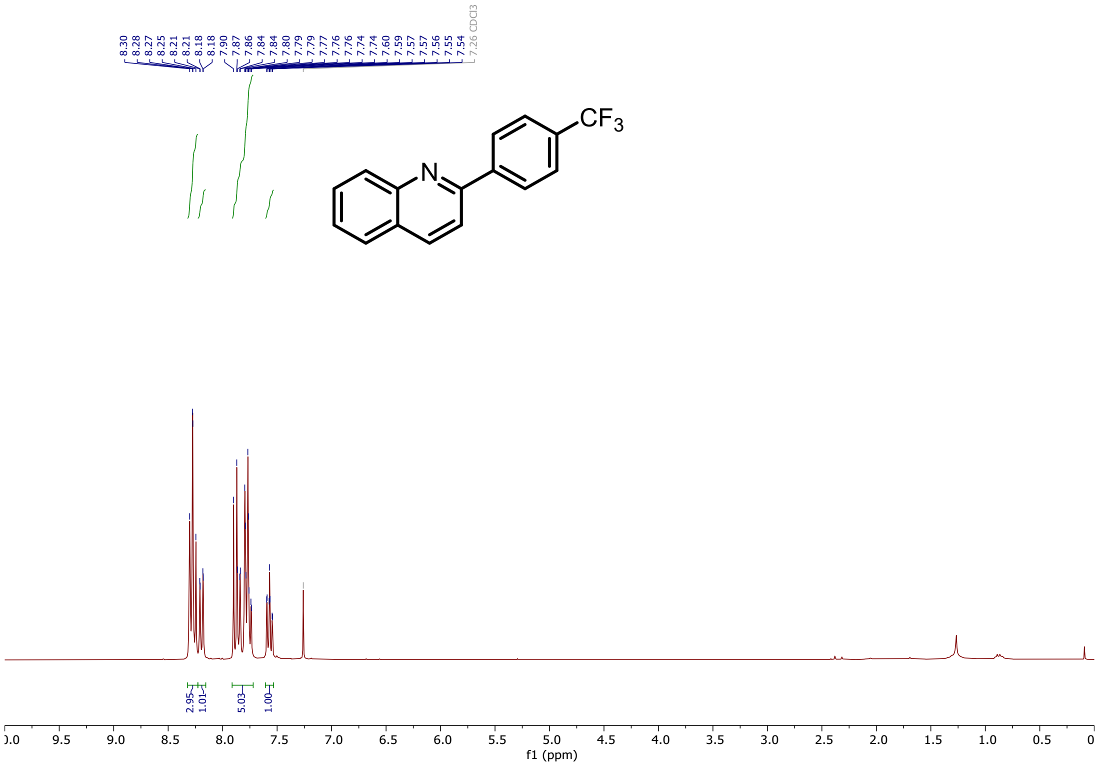

Methyl-4-(quinoline-2-yl)benzoate (2l)

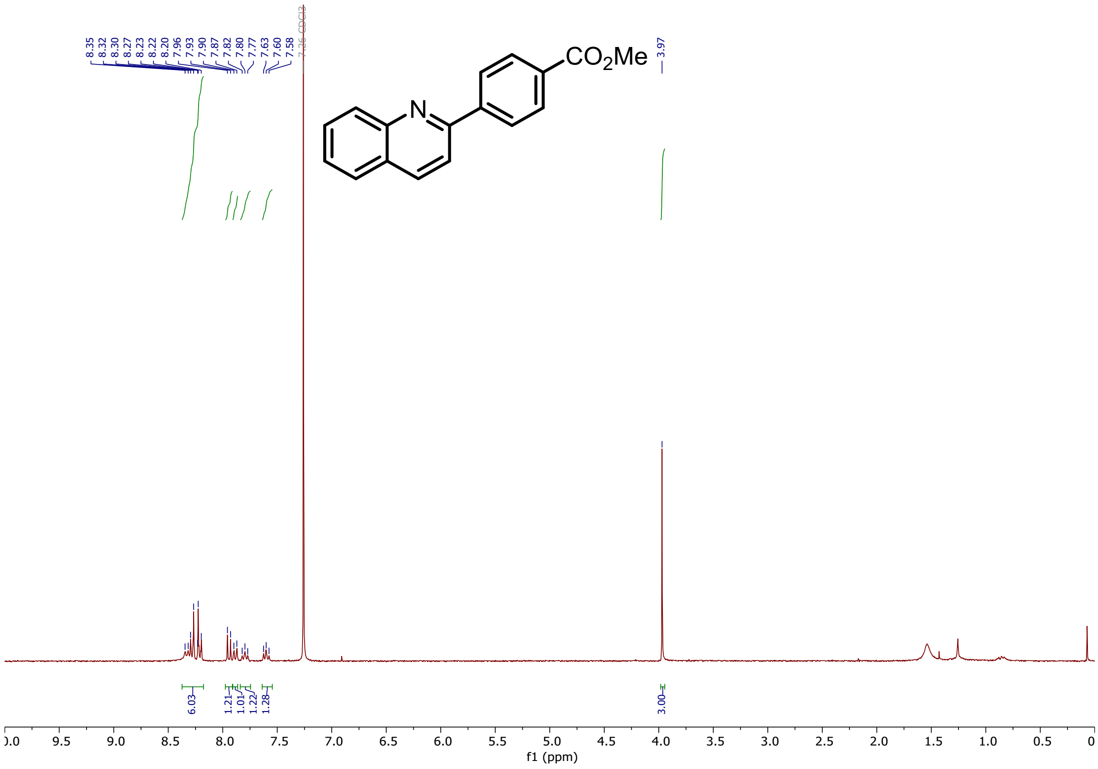

2-(Napthalen-1-yl)quinoline (2o)

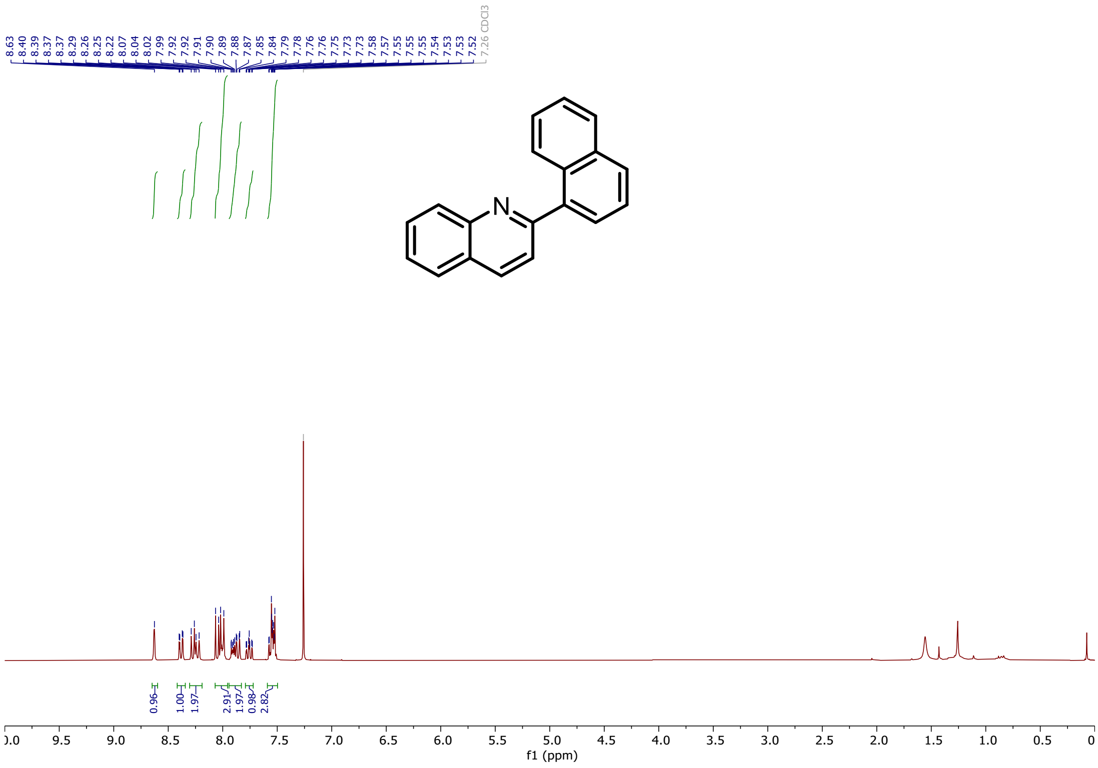

2-(Furan-2-yl)quinoline (2p)

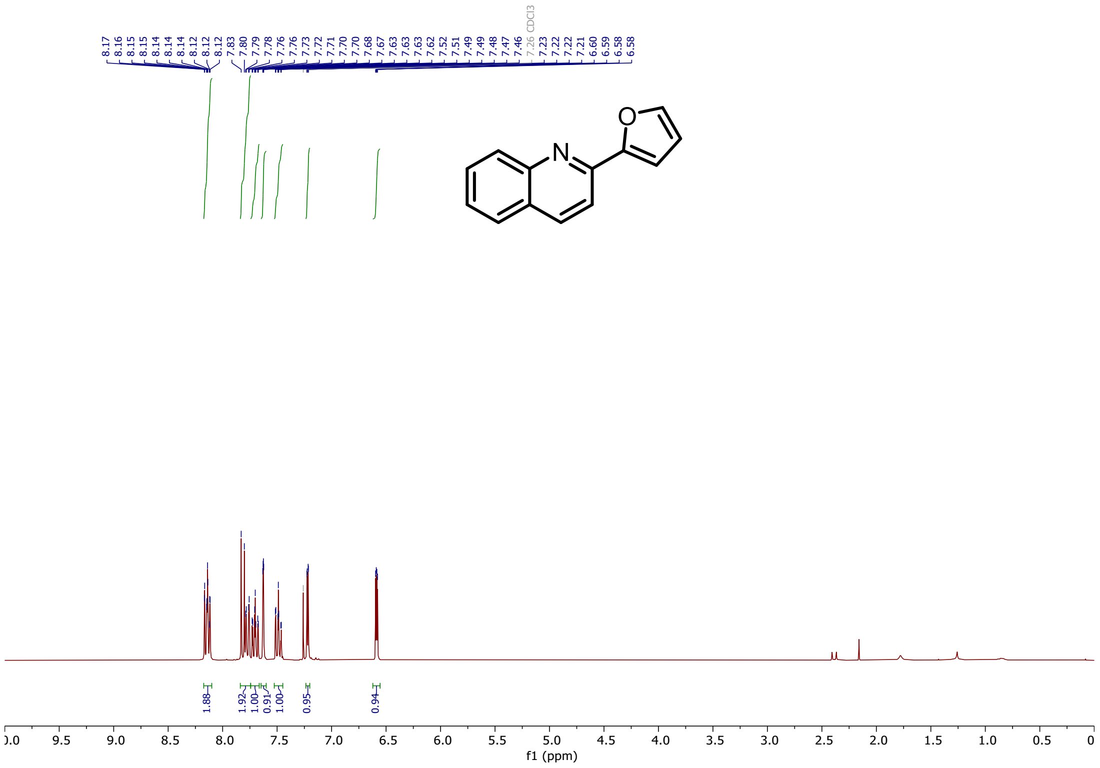

2-(Thiophen-2-yl)quinoline (2q)

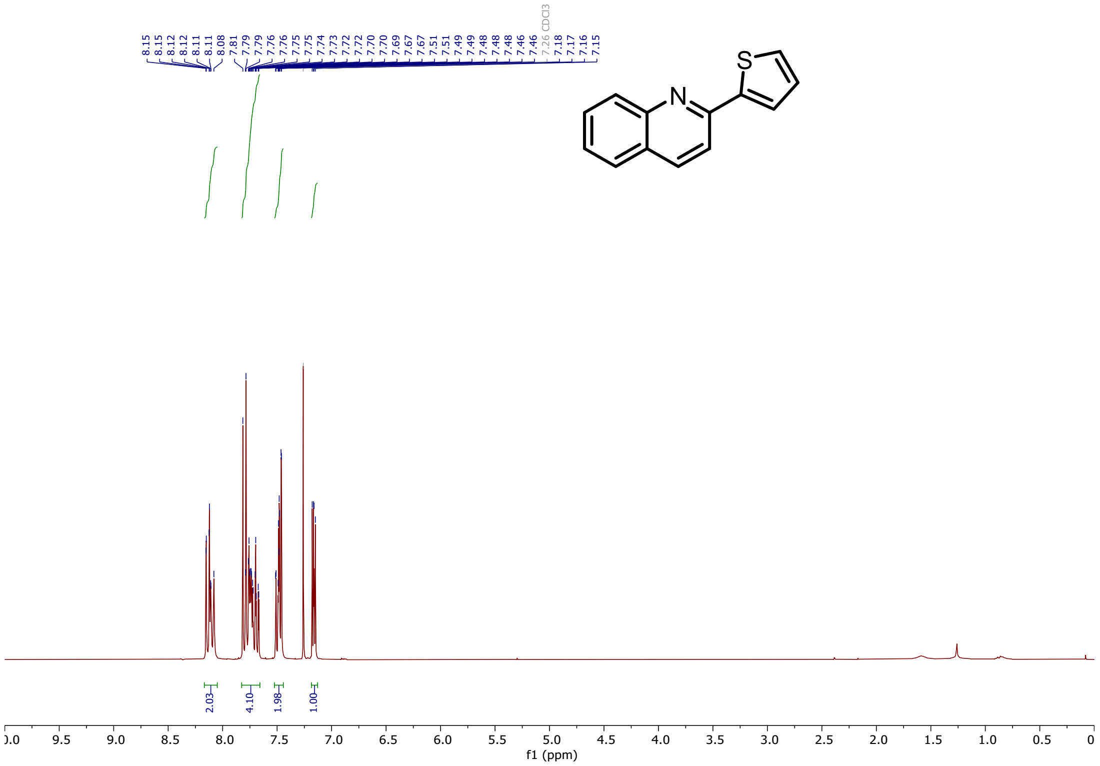

6-Methoxy-2-phenylquinoline (2r)

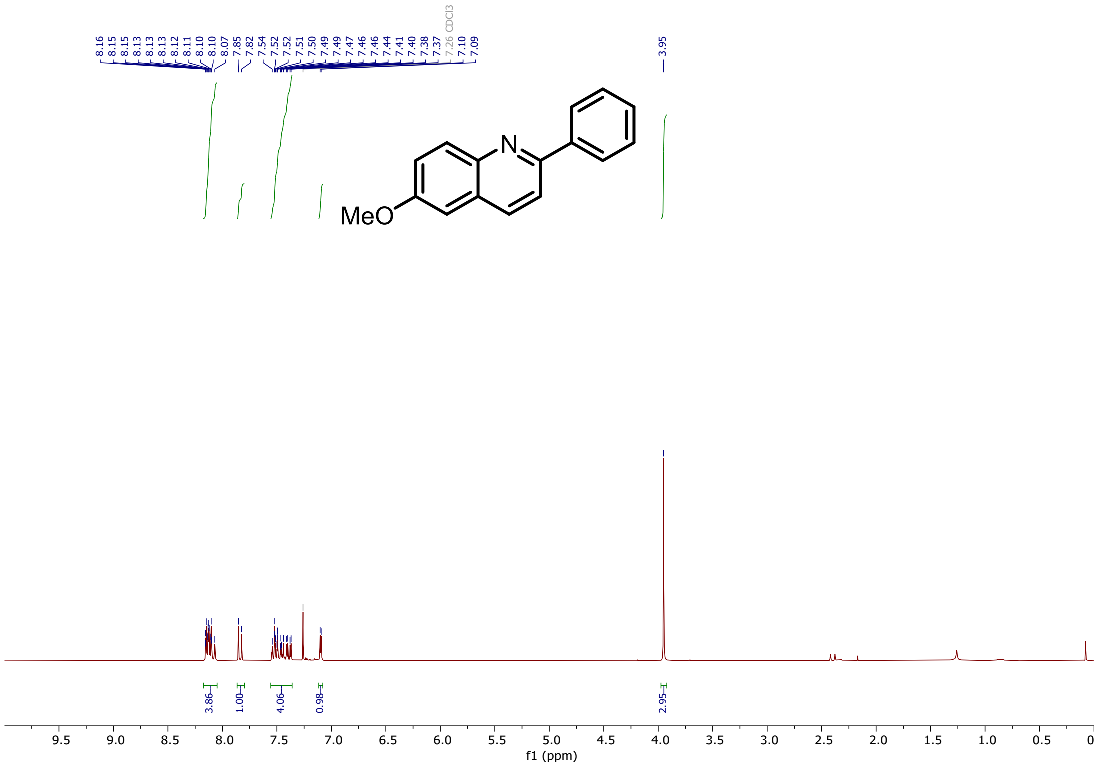

6-Chloro-2-phenylquinoline (2s)

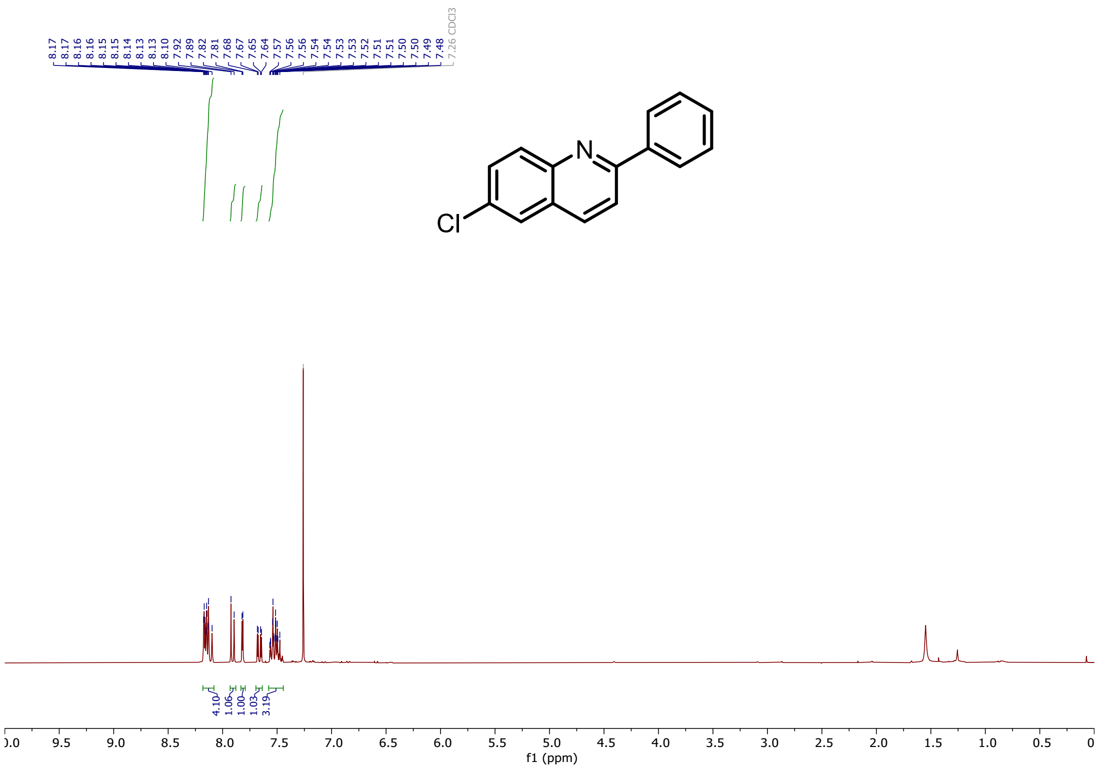

7-Bromo-2-phenylquinoline (2t)

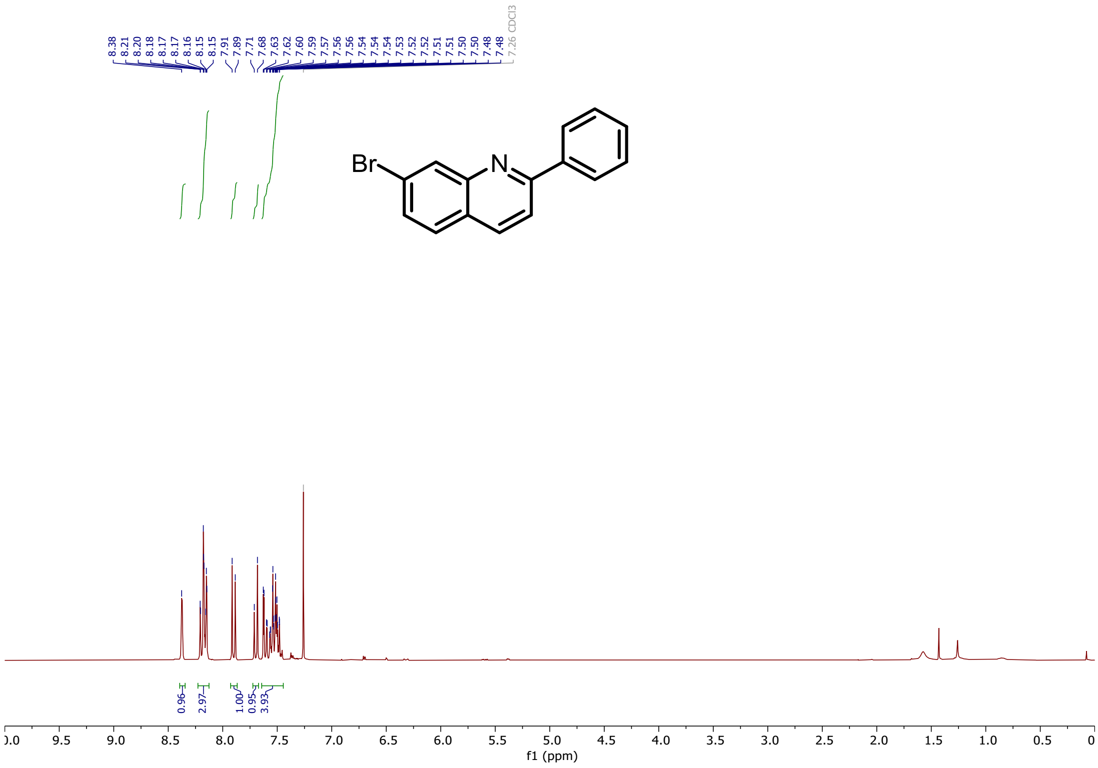

6-Phenyl-[1,3]dioxolo[4,5-*g*]quinoline (2u)

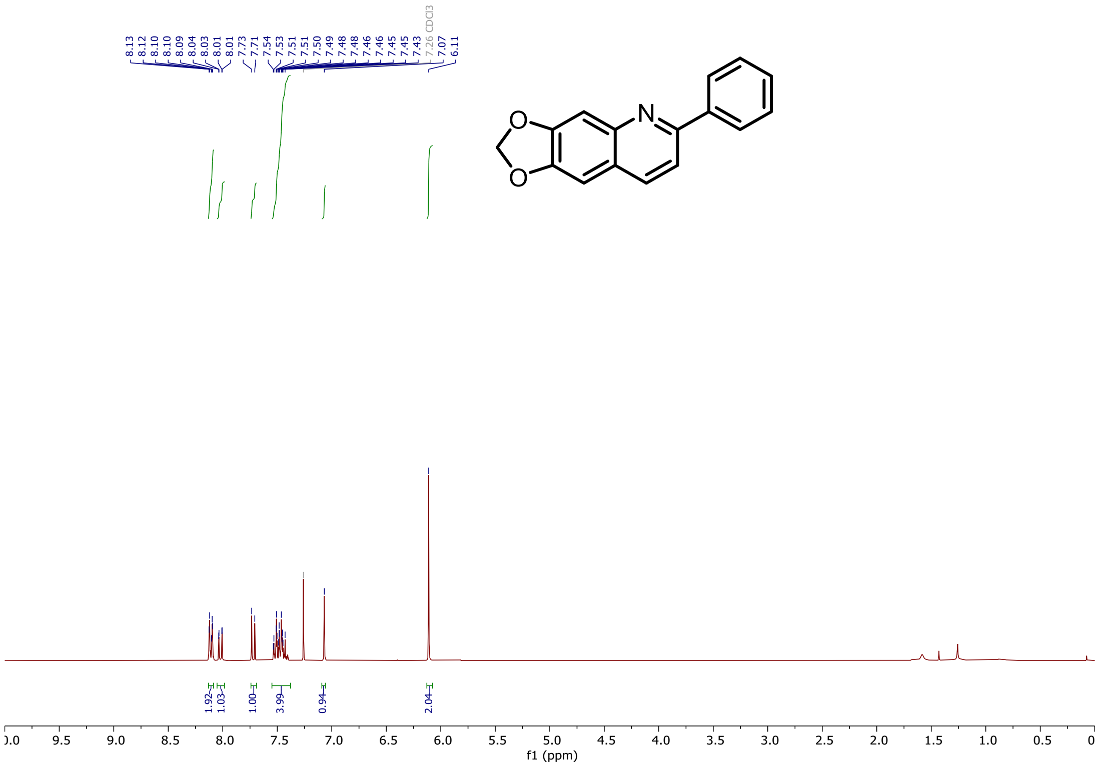

8.- Theoretical calculations

8.1.- Computational details

DFT calculations were performed with Gaussian 16.[16] Geometries were optimized using the M06-2X functional[17] in the gas phase without symmetry restrictions with the 6-31G(d) basis set for all atoms (BS1). Harmonic frequencies were calculated at the same level to characterize the stationary points and to determine the zero-point energies (ZPE). All potential energies were corrected by single-point calculations with the larger 6-311++G(d,p) basis set (BS2). Solvation was introduced implicitly in all cases through the SMD model,[18] with toluene as the solvent (ε = 2.3741). The reported free energies include zero-point energies and thermal corrections calculated at 298 K with M06-2X/BS1

8.2.- Cartesian coordinates (Å) and energies (hartrees) of all the optimized structures.

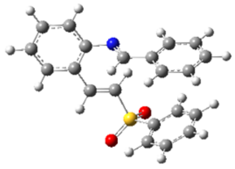

Mod1a

E(M062X/BS1) = -1413.30958522
G(correction)= 0.279993
E(M062X /BS2)$_{toluene}$ = -1413.63326708

Imaginary frequencies: 0

1	0	-3.71448	-3.02807	0.31389
6	0	-3.90142	-1.98782	0.06807
6	0	-5.16565	-1.55833	-0.31604
6	0	-2.83462	-1.08983	0.13657
6	0	-5.38354	-0.21912	-0.63368
1	0	-5.98067	-2.27318	-0.3715
6	0	-3.04675	0.27459	-0.15587
7	0	-1.57021	-1.57576	0.52769
6	0	-4.32816	0.67813	-0.5562
1	0	-6.36739	0.12178	-0.93773
6	0	-1.99828	1.3008	-0.06766
6	0	-0.67375	-1.70937	-0.36461
1	0	-4.48833	1.72588	-0.79625
1	0	-2.14845	2.19453	-0.67348
6	0	-0.90715	1.26689	0.70017
1	0	-0.87624	-1.48092	-1.42122
6	0	0.69265	-2.15584	-0.04717
1	0	-0.63523	0.46843	1.38223
16	0	0.25453	2.59448	0.64064
6	0	1.59625	-2.38555	-1.08638
6	0	1.09292	-2.363	1.27876
8	0	-0.2302	3.59975	-0.30384
8	0	0.60809	2.94751	2.01273
6	0	1.66693	1.78788	-0.09318
6	0	2.88084	-2.84483	-0.81034
1	0	1.28729	-2.20909	-2.11378
6	0	2.37822	-2.80971	1.55299
1	0	0.37729	-2.18077	2.0749
6	0	2.51155	1.04075	0.72424
6	0	1.8676	1.88735	-1.46577
6	0	3.27075	-3.06034	0.50827
1	0	3.57614	-3.03111	-1.62303
1	0	2.68633	-2.97364	2.58094
6	0	3.58719	0.37708	0.14475
1	0	2.33274	1.00276	1.79457
6	0	2.9502	1.2208	-2.03318
1	0	1.19223	2.49332	-2.06149
1	0	4.27186	-3.4207	0.72594
6	0	3.80306	0.46687	-1.22972
1	0	4.25406	-0.21375	0.76389
1	0	3.13128	1.29538	-3.10076
1	0	4.64443	-0.05455	-1.67651

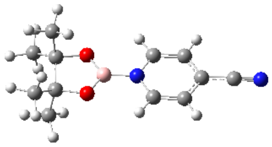

I

E(M062X/BS1) = -751.504263056
G(correction)= 0.230606
E(M062X /BS2)$_{toluene}$ = -751.7288906

Imaginary frequencies: 0

6	0	1.65954	1.18588	-0.17854
6	0	3.01482	1.20434	-0.18176
7	0	0.94273	0.	0.
1	0	1.05175	2.07171	-0.31151
6	0	3.75889	0.	0.
1	0	3.53246	2.14586	-0.32341
6	0	1.65954	-1.18588	0.17854
5	0	-0.49735	0.	0.
6	0	3.01482	-1.20434	0.18176
6	0	5.17358	0.	0.
1	0	1.05175	-2.07171	0.31151
8	0	-1.23576	-1.1288	0.22829
8	0	-1.23576	1.1288	-0.22829
1	0	3.53246	-2.14586	0.32341
7	0	6.33713	0.	0.
6	0	-2.60196	-0.77649	-0.10098
6	0	-2.60196	0.77649	0.10098
6	0	-3.53988	-1.53807	0.81989
6	0	-2.82711	-1.183	-1.55525
6	0	-3.53988	1.53807	-0.81989
6	0	-2.82711	1.183	1.55525
1	0	-4.57531	-1.22072	0.65728
1	0	-3.47263	-2.60833	0.6069
1	0	-3.28256	-1.38085	1.86889
1	0	-2.60626	-2.24816	-1.66179
1	0	-3.86237	-1.00865	-1.86231
1	0	-2.165	-0.6259	-2.22559
1	0	-4.57531	1.22072	-0.65727
1	0	-3.47263	2.60833	-0.6069
1	0	-3.28256	1.38085	-1.86889
1	0	-2.60626	2.24816	1.66179
1	0	-3.86237	1.00865	1.86231
1	0	-2.165	0.6259	2.22559

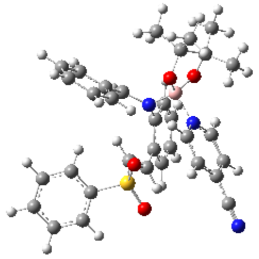

II

E(M062X/BS1) = -2164.83382757
G(correction)= 0.536741
E(M062X /BS2)$_{toluene}$ = -2165.36709941

Imaginary frequencies: 0

1	0	-2.94333	0.17246	2.91053
6	0	-2.14167	-0.54815	3.03427
6	0	-2.18549	-1.51107	4.03728
6	0	-1.07804	-0.49925	2.12956
6	0	-1.17228	-2.46284	4.1321
1	0	-3.01745	-1.52729	4.73454
6	0	-0.04339	-1.45612	2.22644
7	0	-1.08583	0.47074	1.08062
6	0	-0.1238	-2.43975	3.22214
1	0	-1.20478	-3.2253	4.90372
6	0	1.06484	-1.54059	1.26166
6	0	-0.68186	1.73721	1.47633
5	0	-2.10008	0.20984	-0.04962
1	0	0.66045	-3.1903	3.2793
1	0	1.4629	-2.53745	1.06565
6	0	1.60747	-0.5187	0.59666
1	0	-0.77231	1.95451	2.54022
6	0	-0.04993	2.71503	0.66023
7	0	-1.53643	-1.24263	-0.71624
8	0	-2.136	1.15386	-1.10684
8	0	-3.43816	-0.03925	0.40371
1	0	1.3505	0.52777	0.71618
16	0	2.7531	-0.83614	-0.70791
6	0	0.18031	4.00816	1.19946
6	0	0.42927	2.46742	-0.65166
6	0	-0.61858	-1.20257	-1.68768
6	0	-1.90854	-2.41059	-0.17626
6	0	-3.45633	1.70482	-1.14253
6	0	-4.31842	0.53463	-0.56852
8	0	3.04741	-2.27054	-0.74688
8	0	2.2124	-0.17724	-1.90461
6	0	4.2192	0.04227	-0.22446
6	0	0.83502	4.98586	0.47217
1	0	-0.17383	4.22494	2.20449
6	0	1.09212	3.45244	-1.36777
1	0	0.29452	1.48782	-1.09121
6	0	0.00688	-2.35223	-2.14801
1	0	-0.38022	-0.21998	-2.07771
6	0	-1.33517	-3.60548	-0.57749
1	0	-2.67168	-2.34486	0.59258
6	0	-3.78446	2.09722	-2.57553
6	0	-3.47809	2.9401	-0.24052
6	0	-5.59762	0.97179	0.13023
6	0	-4.63911	-0.52227	-1.62929
6	0	4.19667	1.43545	-0.25373
6	0	5.33749	-0.67649	0.18232
6	0	1.30012	4.72095	-0.82023
1	0	0.99016	5.96633	0.91344
1	0	1.4573	3.22072	-2.36486
1	0	0.78405	-2.28397	-2.89914
6	0	-0.35335	-3.5703	-1.57285
1	0	-1.63575	-4.54119	-0.12224
1	0	-4.82337	2.43524	-2.65841
1	0	-3.13099	2.91773	-2.88559
1	0	-3.62954	1.26095	-3.26092
1	0	-2.68048	3.61845	-0.55776
1	0	-4.43682	3.46593	-0.29862
1	0	-3.28715	2.66238	0.80138
1	0	-6.25084	1.51825	-0.55897
1	0	-6.14019	0.09255	0.49075
1	0	-5.37502	1.61129	0.98682
1	0	-5.0232	-1.41772	-1.13035
1	0	-5.39733	-0.16825	-2.33483
1	0	-3.74461	-0.79759	-2.19693
6	0	5.33637	2.12389	0.14697
1	0	3.30731	1.96573	-0.58718
6	0	6.47398	0.02799	0.57011
1	0	5.30676	-1.76125	0.17933
1	0	1.81339	5.48956	-1.38884
6	0	0.27912	-4.79362	-1.99669
6	0	6.46993	1.42126	0.55518
1	0	5.33937	3.20887	0.13553
1	0	7.36222	-0.51096	0.88321
7	0	0.76822	-5.78595	-2.33211
1	0	7.35799	1.96494	0.86221

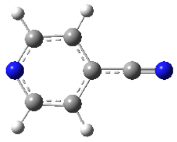

P5

E(M062X/BS1) = -340.383736565
G(correction)= 0.058342
E(M062X /BS2)$_{toluene}$ = -340.48212593

Imaginary frequencies: 0

7	0	-2.19481	-0.00003	-0.00019
6	0	-1.50307	1.14035	0.00026
6	0	-1.50241	-1.14084	-0.00023
6	0	-0.11288	1.20343	0.00001
1	0	-2.08883	2.05611	-0.00047
6	0	-0.11285	-1.20326	0.00021
1	0	-2.08834	-2.05641	-0.00001
1	0	0.40651	2.15467	-0.00038
6	0	0.5938	0.00048	0.00029
1	0	0.40761	-2.1539	0.00004
6	0	2.03505	-0.00069	0.00014
7	0	3.19155	0.00041	-0.00027

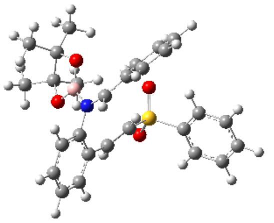

III

E(M062X/BS1) = -1824.42867262
G(correction)= 0.454040
E(M062X /BS2)$_{toluene}$ = -1824.8721641

Imaginary frequencies: 0

1	0	-2.8975	1.26717	-3.32498
6	0	-2.31695	1.99364	-2.76356
6	0	-2.3827	3.34861	-3.07261
6	0	-1.52139	1.54154	-1.71401
6	0	-1.67709	4.26887	-2.30302
1	0	-3.00334	3.68476	-3.89709
6	0	-0.7842	2.46083	-0.94631
7	0	-1.50116	0.14118	-1.40002
6	0	-0.9005	3.82353	-1.24018
1	0	-1.73825	5.32962	-2.52302
6	0	0.09532	2.05336	0.16496
6	0	-0.89866	-0.6985	-2.33805
5	0	-2.17113	-0.25799	-0.1922
1	0	-0.35316	4.53787	-0.63112
1	0	0.1176	2.69379	1.04647
6	0	0.88972	0.98346	0.14515
1	0	-0.97705	-0.3657	-3.36955
6	0	-0.09085	-1.83243	-2.05665
8	0	-2.39381	-1.55372	0.18855
8	0	-2.66872	0.67543	0.68471
1	0	0.98617	0.28693	-0.68266
16	0	1.82991	0.55093	1.57646
6	0	0.40158	-2.5904	-3.15172
6	0	0.32345	-2.2209	-0.75675
6	0	-3.26863	-1.4942	1.33772
6	0	-3.00297	-0.05302	1.88808
8	0	1.70187	1.6224	2.56359
8	0	1.47765	-0.82864	1.92509
6	0	3.50484	0.53211	0.97531
6	0	1.25338	-3.66237	-2.95893
1	0	0.09911	-2.31187	-4.15822
6	0	1.1876	-3.29053	-0.5785
1	0	-0.01974	-1.68189	0.1169
6	0	-2.89327	-2.61764	2.28993
6	0	-4.69362	-1.67743	0.82079
6	0	-4.20355	0.60931	2.54342
6	0	-1.78283	0.01381	2.80286
6	0	3.91365	-0.53639	0.18015
6	0	4.35486	1.58322	1.3003
6	0	1.65952	-4.02264	-1.66995
1	0	1.61141	-4.2228	-3.81758
1	0	1.49727	-3.54481	0.43149
1	0	-3.47505	-2.54635	3.21528
1	0	-3.10919	-3.58146	1.82078
1	0	-1.83002	-2.58672	2.53631
1	0	-4.74849	-2.61805	0.26661
1	0	-5.41566	-1.7117	1.64209
1	0	-4.97075	-0.86274	0.14427
1	0	-4.53975	0.02382	3.40583
1	0	-3.9225	1.60564	2.89538
1	0	-5.03345	0.71487	1.84193
1	0	-1.51344	1.0622	2.96277
1	0	-1.98761	-0.44332	3.77587
1	0	-0.91784	-0.49163	2.36071
6	0	5.21582	-0.53993	-0.30845
1	0	3.22481	-1.34844	-0.0407
6	0	5.65816	1.56087	0.8115
1	0	3.99263	2.38664	1.93359
1	0	2.33105	-4.86199	-1.5209
6	0	6.08344	0.50503	0.00786
1	0	5.55521	-1.36153	-0.9307
1	0	6.34181	2.36629	1.05947
1	0	7.10011	0.49384	-0.3727

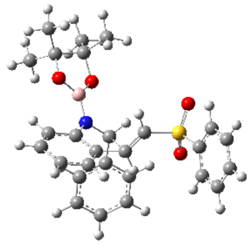

TScyc(5)

E(M062X/BS1) = -1824.41209806
G(correction)= 0.450885
E(M062X /BS2)$_{toluene}$ = -1824.85676015

Imaginary frequencies: 1(-458.7154 cm^{-1})

1	0	3.03012	0.95791	2.56336
6	0	1.95727	1.04286	2.69404
6	0	1.4009	1.34653	3.93351
6	0	1.10972	0.83417	1.60845
6	0	0.02027	1.44636	4.09099
1	0	2.05572	1.51218	4.7834
6	0	-0.28155	0.92559	1.75466
7	0	1.54701	0.50982	0.30212
6	0	-0.8202	1.22861	3.00059
1	0	-0.40229	1.68764	5.06079
6	0	-1.10385	0.61719	0.54564
6	0	0.62596	0.88121	-0.70203
5	0	2.60798	-0.40471	0.01474
1	0	-1.89874	1.28499	3.11543
1	0	-1.6196	1.42514	0.02953
6	0	-1.59188	-0.654	0.38266
1	0	0.48517	0.1498	-1.4938
6	0	0.47382	2.28497	-1.07245
8	0	2.88914	-0.80801	-1.2663
8	0	3.42756	-0.95981	0.96657
1	0	-1.25532	-1.51671	0.94857
16	0	-2.6177	-1.01757	-0.99078
6	0	-0.41247	2.614	-2.11398

6	0	1.14924	3.31596	-0.40071
6	0	3.8036	-1.91877	-1.14054
6	0	4.4818	-1.63174	0.23975
8	0	-2.65195	0.17315	-1.84633
8	0	-2.20864	-2.3169	-1.52395
6	0	-4.25535	-1.21897	-0.31018
6	0	-0.60249	3.93912	-2.47941
1	0	-0.96209	1.818	-2.61028
6	0	0.95375	4.64007	-0.7747
1	0	1.83817	3.07184	0.40196
6	0	4.74992	-1.91325	-2.3295
6	0	2.96137	-3.19282	-1.12997
6	0	4.89906	-2.87029	1.01492
6	0	5.64585	-0.64908	0.13057
6	0	-4.68143	-2.49002	0.06188
6	0	-5.06336	-0.09641	-0.15055
6	0	0.0798	4.95776	-1.81279
1	0	-1.28653	4.18049	-3.28714
1	0	1.48917	5.42883	-0.25491
1	0	5.51696	-2.68629	-2.21238
1	0	4.18825	-2.1233	-3.24365
1	0	5.23852	-0.94387	-2.44405
1	0	2.34879	-3.21806	-2.03471
1	0	3.58971	-4.08812	-1.106
1	0	2.29119	-3.21191	-0.2646
1	0	5.6301	-3.45131	0.4426
1	0	5.36187	-2.57274	1.9599
1	0	4.04048	-3.50559	1.23975
1	0	5.93492	-0.3315	1.13609
1	0	6.51324	-1.10843	-0.35258
1	0	5.35604	0.23794	-0.44157
6	0	-5.94985	-2.63578	0.61674
1	0	-4.03099	-3.3426	-0.10552
6	0	-6.32859	-0.25522	0.40467
1	0	-4.70387	0.87399	-0.47851
1	0	-0.06967	5.9936	-2.10118
6	0	-6.76785	-1.52132	0.79014
1	0	-6.30215	-3.62034	0.90712
1	0	-6.97559	0.60698	0.53039
1	0	-7.75698	-1.64039	1.22136

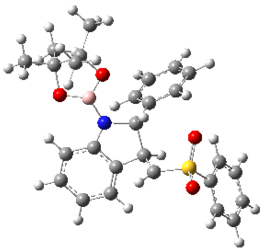

IVa(5)

E(M062X/BS1) = -1824.44335902

G(correction)= 0.454918

E(M062X /BS2)$_{toluene}$ = -1824.88626212

Imaginary frequencies: 0

1	0	2.7315	-2.9665	0.95972
6	0	1.65163	-3.00744	1.03904
6	0	1.00199	-4.10083	1.61181
6	0	0.86558	-1.95798	0.56565
6	0	-0.38761	-4.15694	1.71343
1	0	1.59847	-4.92679	1.98769
6	0	-0.5277	-2.00795	0.66445
7	0	1.28034	-0.7681	-0.04143
6	0	-1.16257	-3.10064	1.23069
1	0	-0.86554	-5.0202	2.16427
6	0	-1.12724	-0.77495	0.02692
6	0	0.13849	0.14443	-0.20482
5	0	2.60324	-0.33573	-0.31864
1	0	-2.24733	-3.13076	1.29396
1	0	-1.81368	-0.25518	0.70684
6	0	-1.84596	-1.10756	-1.23345
1	0	0.12884	0.55927	-1.21606
6	0	0.15853	1.2876	0.79191
8	0	2.8577	0.90628	-0.84913
8	0	3.73866	-1.07912	-0.08073
1	0	-1.55646	-1.92747	-1.8829
16	0	-2.94048	0.06014	-1.95375
6	0	-0.42443	2.50854	0.44473
6	0	0.69657	1.1237	2.06937
6	0	4.26029	0.92131	-1.18419
6	0	4.84286	-0.15525	-0.21113
8	0	-2.23577	1.34302	-2.03557
8	0	-3.5257	-0.55606	-3.14048
6	0	-4.22291	0.24581	-0.73001
6	0	-0.47035	3.55126	1.36688
1	0	-0.83916	2.63293	-0.55283
6	0	0.65089	2.1685	2.98927
1	0	1.15572	0.17692	2.3431
6	0	4.79952	2.3268	-0.97449
6	0	4.3792	0.5134	-2.65131
6	0	6.05371	-0.90589	-0.73867
6	0	5.11912	0.40569	1.18287
6	0	-5.32896	-0.5981	-0.7894
6	0	-4.08458	1.21391	0.26186
6	0	0.06664	3.38391	2.64145
1	0	-0.9163	4.50031	1.08422
1	0	1.07327	2.03185	3.98034
1	0	5.88487	2.3482	-1.12082
1	0	4.34185	3.00514	-1.69964
1	0	4.56819	2.69335	0.02729
1	0	3.76775	1.18968	-3.25425
1	0	5.41427	0.57145	-3.00122
1	0	4.01431	-0.50728	-2.80365
1	0	6.87803	-0.21228	-0.93615
1	0	6.3889	-1.63218	0.00708
1	0	5.81669	-1.44405	-1.65834
1	0	5.31424	-0.42607	1.86512
1	0	5.9887	1.06958	1.18325
1	0	4.25282	0.96231	1.55613
6	0	-6.31991	-0.46963	0.17853
1	0	-5.40815	-1.32223	-1.59422
6	0	-5.08128	1.32722	1.22701
1	0	-3.21635	1.8676	0.2647
1	0	0.03519	4.19865	3.35838
6	0	-6.19257	0.48737	1.18452
1	0	-7.19394	-1.11186	0.14528

1	0	-4.99188	2.07528	2.00787
1	0	-6.96844	0.58272	1.93778

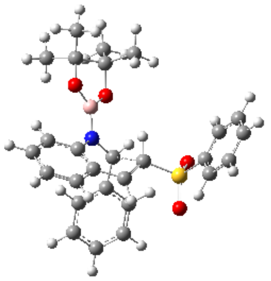

TScyc(6)

E(M062X/BS1) = -1824.41164278
G(correction)= 0.453592
E(M062X /BS2)$_{toluene}$ = -1824.85691040

Imaginary frequencies: 1(-531.7454 cm^{-1})

1	0	3.28428	1.39617	1.69251
6	0	2.30462	1.57312	2.12283
6	0	2.18165	2.18137	3.36445
6	0	1.17318	1.19767	1.39415
6	0	0.91646	2.4617	3.88668
1	0	3.07491	2.46202	3.91334
6	0	-0.11594	1.42656	1.93686
7	0	1.34317	0.6544	0.08167
6	0	-0.20929	2.09646	3.16993
1	0	0.81574	2.96458	4.84284
6	0	-1.31964	1.04717	1.22496
6	0	0.40073	0.92799	-0.92409
5	0	2.28446	-0.4041	-0.15875
1	0	-1.20057	2.30417	3.56427
1	0	-2.20883	1.66413	1.33172
6	0	-1.2842	0.08854	0.2434
1	0	0.3693	0.18306	-1.7129
6	0	-0.00186	2.2783	-1.28433
8	0	2.399	-1.00451	-1.38937
8	0	3.12221	-0.93269	0.79082
1	0	-0.63547	-0.78235	0.27593
16	0	-2.7134	-0.18185	-0.75679
6	0	-0.92387	2.42996	-2.33724
6	0	0.46887	3.42802	-0.63007
6	0	3.19873	-2.18891	-1.18511
6	0	4.02846	-1.80924	0.08472
8	0	-3.59113	0.98041	-0.63255
8	0	-2.24462	-0.6401	-2.0684
6	0	-3.53386	-1.57113	0.00956
6	0	-1.35893	3.68892	-2.7198
1	0	-1.30574	1.54245	-2.8355
6	0	0.03179	4.68829	-1.02492
1	0	1.19489	3.3413	0.17087
6	0	4.02392	-2.44771	-2.43445
6	0	2.22941	-3.34244	-0.9329
6	0	4.38329	-2.97833	0.98816
6	0	5.27372	-0.99021	-0.24868
6	0	-3.2363	-2.85866	-0.42485
6	0	-4.44094	-1.33204	1.03782
6	0	-0.88421	4.82577	-2.06394
1	0	-2.07533	3.78545	-3.52943
1	0	0.41218	5.56848	-0.5154
1	0	4.71164	-3.28462	-2.27255
1	0	3.3603	-2.70439	-3.26456
1	0	4.60038	-1.56556	-2.71886
1	0	1.5359	-3.40837	-1.7754
1	0	2.75646	-4.29625	-0.8368
1	0	1.64818	-3.17186	-0.02054
1	0	4.99359	-3.70884	0.44648
1	0	4.95931	-2.61776	1.84467
1	0	3.48732	-3.47535	1.36446
1	0	5.68525	-0.58234	0.67858
1	0	6.04057	-1.6044	-0.72972
1	0	5.02651	-0.15603	-0.91309
6	0	-3.86224	-3.93608	0.19619
1	0	-2.54332	-2.99841	-1.24854
6	0	-5.06055	-2.41671	1.64954
1	0	-4.66257	-0.31044	1.33063
1	0	-1.22689	5.81157	-2.36279
6	0	-4.76831	-3.71435	1.23115
1	0	-3.64856	-4.94793	-0.13294
1	0	-5.77595	-2.25052	2.44845
1	0	-5.25551	-4.5576	1.71082

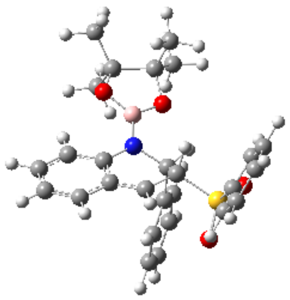

IVa(6)

E(M062X/BS1) = -1824.45874988
G(correction)= 0.456861
E(M062X /BS2)$_{toluene}$ = -1824.90203513

Imaginary frequencies: 0

1	0	-2.89947	1.4238	-2.01497
6	0	-1.88379	1.66043	-2.30564
6	0	-1.64436	2.52257	-3.37082
6	0	-0.82448	1.08448	-1.60542
6	0	-0.33571	2.84302	-3.75173
1	0	-2.48613	2.95803	-3.89965
6	0	0.51722	1.40078	-1.98117
7	0	-1.03138	0.1883	-0.53798
6	0	0.72423	2.29236	-3.06292
1	0	-0.15664	3.52268	-4.5784
6	0	1.60344	0.82026	-1.29723
6	0	-0.00667	0.1732	0.52575
5	0	-2.20846	-0.58675	-0.32981
1	0	1.74763	2.53146	-3.33981

1	0	2.61913	0.99651	-1.63701
6	0	1.34873	-0.0937	-0.15568
1	0	-0.22119	-0.69052	1.15591
6	0	-0.13385	1.41022	1.40242
8	0	-2.37867	-1.34506	0.81257
8	0	-3.27019	-0.68953	-1.20055
1	0	1.32891	-1.14518	-0.48635
16	0	2.71128	-0.10464	1.06
6	0	-1.067	1.34062	2.4431
6	0	0.54891	2.60841	1.18753
6	0	-3.53027	-2.18152	0.58277
6	0	-4.32701	-1.354	-0.47586
8	0	3.27094	1.23633	1.2084
8	0	2.2224	-0.86866	2.20703
6	0	3.94786	-1.09263	0.22863
6	0	-1.31559	2.44214	3.25276
1	0	-1.5999	0.40725	2.6099
6	0	0.3019	3.7114	2.00303
1	0	1.29419	2.68392	0.40414
6	0	-4.25257	-2.39565	1.90278
6	0	-3.02044	-3.5103	0.02832
6	0	-5.14645	-2.18267	-1.45086
6	0	-5.18759	-0.2635	0.16015
6	0	3.85073	-2.48056	0.28351
6	0	4.97932	-0.45336	-0.45148
6	0	-0.62954	3.63469	3.03246
1	0	-2.03756	2.36817	4.06045
1	0	0.84787	4.63367	1.83059
1	0	-5.1866	-2.94539	1.74478
1	0	-3.61992	-2.98115	2.57555
1	0	-4.48006	-1.44495	2.38892
1	0	-2.29976	-3.93225	0.73375
1	0	-3.83584	-4.22676	-0.1086
1	0	-2.5177	-3.36424	-0.93301
1	0	-5.89751	-2.77299	-0.91507
1	0	-5.66555	-1.51996	-2.14866
1	0	-4.512	-2.85722	-2.0287
1	0	-5.54766	0.40529	-0.62641
1	0	-6.05222	-0.6866	0.68
1	0	-4.60385	0.32779	0.87341
6	0	4.80604	-3.24532	-0.37754
1	0	3.05156	-2.94186	0.85626
6	0	5.9301	-1.23021	-1.10882
1	0	5.03666	0.63063	-0.43789
1	0	-0.81581	4.497	3.66551
6	0	5.83938	-2.61981	-1.07461
1	0	4.75088	-4.32848	-0.34196
1	0	6.74524	-0.75051	-1.64078
1	0	6.58388	-3.22104	-1.58708

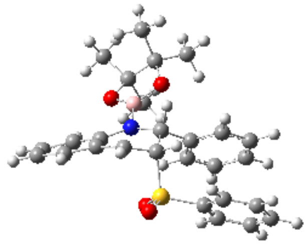

IVb

E(M062X/BS1) = -1824.45461132

G(correction)= 0.459172

E(M062X /BS2)$_{toluene}$ = -1824.89821523

Imaginary frequencies: 0

1	0	2.7562	1.94605	-1.50166
6	0	2.15418	2.64665	-0.93919
6	0	2.35744	4.01378	-1.08857
6	0	1.19071	2.16601	-0.04951
6	0	1.60121	4.94001	-0.36011
1	0	3.11584	4.35994	-1.78375
6	0	0.36607	3.09829	0.64671
7	0	1.06881	0.79094	0.22599
6	0	0.61644	4.48293	0.48892
1	0	1.77154	6.00464	-0.48086
6	0	-0.70659	2.63299	1.43247
6	0	-0.09004	0.24877	0.95518
5	0	2.1943	-0.10291	0.19057
1	0	-0.01583	5.17906	1.03289
1	0	-1.33239	3.33586	1.9686
6	0	-1.21979	1.27208	1.172
1	0	0.24147	-0.0007	1.97305
6	0	-0.57905	-1.05867	0.33595
8	0	2.17793	-1.27411	0.91336
8	0	3.35815	0.06771	-0.52305
1	0	-1.86842	0.90807	1.97643
16	0	-2.40196	1.47108	-0.25743
6	0	-1.2706	-1.96449	1.14041
6	0	-0.33873	-1.38283	-0.99896
6	0	3.49449	-1.84932	0.81554
6	0	4.03316	-1.21039	-0.50392
8	0	-3.18726	2.66078	0.07261
8	0	-1.64804	1.39093	-1.50834
6	0	-3.52892	0.08645	-0.1812
6	0	-1.71736	-3.17657	0.62445
1	0	-1.45407	-1.72276	2.18587
6	0	-0.76282	-2.60701	-1.51114
1	0	0.16941	-0.66542	-1.63664
6	0	3.36067	-3.36355	0.78345
6	0	4.27598	-1.40185	2.04901
6	0	5.53366	-0.97488	-0.53729
6	0	3.57768	-1.96889	-1.7495
6	0	-3.56824	-0.8152	-1.23591
6	0	-4.37237	-0.03324	0.92168
6	0	-1.45277	-3.5069	-0.70287
1	0	-2.26033	-3.86772	1.26186
1	0	-0.56166	-2.85159	-2.55039
1	0	4.33071	-3.83359	0.58881
1	0	2.99548	-3.71807	1.75126
1	0	2.64931	-3.67787	0.01693

1	0	3.72498	-1.70474	2.94328
1	0	5.27018	-1.85762	2.08039
1	0	4.38823	-0.31324	2.06625
1	0	6.07166	-1.92297	-0.43088
1	0	5.81346	-0.52825	-1.49537
1	0	5.84741	-0.29855	0.25996
1	0	3.79584	-1.3613	-2.63182
1	0	4.09703	-2.92689	-1.84699
1	0	2.4989	-2.15701	-1.71715
6	0	-4.4596	-1.88307	-1.16977
1	0	-2.90227	-0.67582	-2.08047
6	0	-5.24807	-1.11081	0.98255
1	0	-4.35573	0.71812	1.70623
1	0	-1.78664	-4.45885	-1.10485
6	0	-5.28751	-2.03577	-0.06113
1	0	-4.49902	-2.60066	-1.98326
1	0	-5.90929	-1.2228	1.83585
1	0	-5.97525	-2.87437	-0.01159

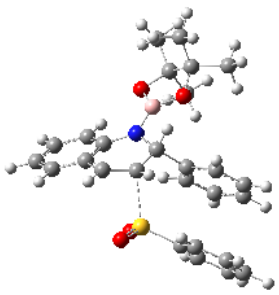

TSelim

E(M062X/BS1) = -1824.43978031
G(correction)= 0.453836
E(M062X /BS2)$_{toluene}$ = -1824.88589649

Imaginary frequencies: 1(-299.6675 cm^{-1})

1	0	2.85184	1.91637	-1.46706
6	0	2.16495	2.61117	-1.00286
6	0	2.18441	3.95473	-1.36136
6	0	1.27099	2.1549	-0.02953
6	0	1.31444	4.87489	-0.77267
1	0	2.88986	4.28479	-2.11788
6	0	0.36544	3.07644	0.55169
7	0	1.28065	0.82464	0.4233
6	0	0.41021	4.42858	0.17373
1	0	1.3372	5.91954	-1.06343
6	0	-0.61311	2.60147	1.48208
6	0	0.10929	0.2267	1.09162
5	0	2.39887	-0.06385	0.32028
1	0	-0.29899	5.11348	0.63031
1	0	-1.26357	3.31785	1.97359
6	0	-0.90959	1.25478	1.55177
1	0	0.47955	-0.24168	2.01318
6	0	-0.48035	-0.91415	0.26399
8	0	2.35763	-1.298	0.93046
8	0	3.57627	0.16092	-0.35639
1	0	-1.57536	0.90616	2.33963
6	0	-1.1303	-1.96283	0.91543
6	0	-0.3364	-0.95916	-1.12277
6	0	3.67453	-1.86858	0.80589
6	0	4.23432	-1.12327	-0.44687
6	0	-1.61815	-3.05055	0.19818
1	0	-1.23801	-1.9368	1.99827
6	0	-0.8048	-2.05807	-1.84002
1	0	0.14616	-0.13367	-1.63828
6	0	3.5397	-3.3742	0.64485
6	0	4.4393	-1.52877	2.08347
6	0	5.73782	-0.90704	-0.44659
6	0	3.77793	-1.76173	-1.75789
6	0	-1.44014	-3.1081	-1.18212
1	0	-2.12488	-3.85799	0.71788
1	0	-0.67788	-2.08786	-2.91843
1	0	4.51366	-3.82802	0.4322
1	0	3.15453	-3.8077	1.57187
1	0	2.84588	-3.62293	-0.16052
1	0	3.87445	-1.90278	2.94146
1	0	5.43139	-1.99005	2.09103
1	0	4.55513	-0.44591	2.19295
1	0	6.26206	-1.86831	-0.41974
1	0	6.03327	-0.38142	-1.3588
1	0	6.05249	-0.3084	0.41024
1	0	4.01754	-1.08469	-2.58231
1	0	4.28025	-2.71747	-1.9342
1	0	2.69523	-1.92969	-1.75218
1	0	-1.80653	-3.96265	-1.74323
16	0	-2.7157	1.37623	0.07043
8	0	-3.508	2.37475	0.80876
8	0	-2.19439	1.67804	-1.27228
6	0	-3.7524	-0.08147	-0.05914
6	0	-3.81147	-0.77758	-1.25877
6	0	-4.45763	-0.49139	1.07049
6	0	-4.61061	-1.9167	-1.32795
1	0	-3.24382	-0.42033	-2.11124
6	0	-5.23641	-1.63947	0.99228
1	0	-4.41175	0.09793	1.98157
6	0	-5.31172	-2.35055	-0.20648
1	0	-4.67804	-2.46954	-2.26005
1	0	-5.79629	-1.97363	1.86009
1	0	-5.92669	-3.24338	-0.26552

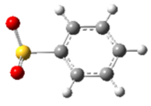

PhSO2$^{\cdot}$

E(M062X/BS1) = -779.99367239
G(correction)= 0.066617
E(M062X /BS2)$_{toluene}$ = -780.14273005

Imaginary frequencies: 0

16	0	1.69762	0.	-0.25282
8	0	2.18257	-1.29074	0.26605
8	0	2.18259	1.29074	0.26607
6	0	-0.09144	0.	-0.08459
6	0	-0.7571	1.22044	-0.05444

6	0	-0.75716	-1.22049	-0.05443
6	0	-2.14592	1.21054	0.03211
1	0	-0.19349	2.14714	-0.07768
6	0	-2.14589	-1.21053	0.0321
1	0	-0.19345	-2.14713	-0.0777
6	0	-2.83572	0.00003	0.07321
1	0	-2.68889	2.14916	0.07048
1	0	-2.68897	-2.14909	0.07061
1	0	-3.91909	-0.00003	0.13864

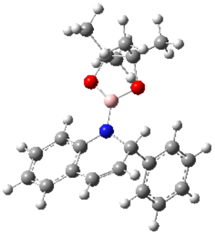

V

E(M062X/BS1) = -1044.44928287

G(correction)= 0.366448

E(M062X /BS2)$_{toluene}$ = -1044.74792679

Imaginary frequencies: 0

1	0	0.01034	-1.83263	1.74603
6	0	-0.87581	-2.10746	1.18782
6	0	-1.70151	-3.13157	1.63924
6	0	-1.17755	-1.4307	0.0032
6	0	-2.844	-3.4929	0.92868
1	0	-1.45042	-3.64539	2.56222
6	0	-2.33558	-1.78742	-0.71815
7	0	-0.348	-0.41432	-0.51455
6	0	-3.15181	-2.81648	-0.24471
1	0	-3.48679	-4.29043	1.28656
6	0	-2.64335	-1.06434	-1.95124
6	0	-0.98939	0.65553	-1.30646
5	0	1.04855	-0.31205	-0.27585
1	0	-4.03904	-3.08136	-0.81481
1	0	-3.40677	-1.47417	-2.60814
6	0	-2.01871	0.07855	-2.24339
1	0	-0.18974	1.11017	-1.89712
6	0	-1.55093	1.743	-0.39281
8	0	1.79069	0.75216	-0.759
8	0	1.81679	-1.21988	0.42116
1	0	-2.25717	0.64692	-3.13753
6	0	-0.71331	2.78838	0.00624
6	0	-2.85371	1.68923	0.10391
6	0	3.17646	0.40677	-0.56585
6	0	3.09877	-0.59523	0.63007
6	0	-1.17151	3.76657	0.88326
1	0	0.30566	2.8213	-0.37206
6	0	-3.3109	2.66496	0.98684
1	0	-3.51197	0.88247	-0.20495
6	0	3.96468	1.67572	-0.28463
6	0	3.66352	-0.25314	-1.85447
6	0	4.17428	-1.669	0.63535
6	0	3.03803	0.11182	1.98343
6	0	-2.47333	3.70645	1.37608
1	0	-0.51405	4.57839	1.18024
1	0	-4.32603	2.61224	1.36888
1	0	5.00309	1.43519	-0.03263
1	0	3.96736	2.31091	-1.17492
1	0	3.52362	2.24156	0.53844
1	0	3.49134	0.43273	-2.68821
1	0	4.73178	-0.48488	-1.80763
1	0	3.11218	-1.17812	-2.05028
1	0	5.16954	-1.2157	0.69666
1	0	4.03901	-2.31796	1.50495
1	0	4.121	-2.28709	-0.26276
1	0	2.77389	-0.62044	2.75133
1	0	4.00041	0.5608	2.24675
1	0	2.27367	0.89589	1.97896
1	0	-2.83354	4.46945	2.05952

References

[1] Z-L. Song, Y. Hou, F. Bai and J. Fang, Generation of Potent Nrf2 Activators via Tuning the Electrophilicity and Steric Hindrance of Vinyl Sulfones for Neuroprotection, *Bioorg. Chem.*, **2021**, 107, 104520-104536.

[2] P. Seubert, M. Freund, R. Rudolf, Y. Lin, L. Altevogt, U. Bilitewski, A. Baro and S. Laschat, Buchwald-Hartwig versus Microwave-Assisted Amination of Chloroquinolines: En Route to the Pyoverdin Chromophore, *Synlett*, 2020, **31**, 1177-1181.

[3] N.V. Shcherbakov, P.F. Kotikova, E.I. Chikunova, D.V. Dar'in, V.Y. Kikishkin and A.Y. Dubovtsev, Gold-Catalyzed Annulation of Ynamides witg Aminocarbonyls as a Route to 2-Aminoquinolines Diversely Substituted at the 4th-Position, *Adv. Synth. Catal.*, 2023, ***365***, 2428-2434.

[4] S. Morales, F.G. Guijarro, J.L. García-Ruano and M.B. Cid, A General Aminocatalytic Method for the Synthesis of Aldimines, *J. Am. Chem. Soc.,* 2014, **136**, 1082-1089.

[5] J. Zhou, G. Wu, X. Jie and W. Su, Pd- Catalyzed Cross-Coupling of Aryl Carboxylic Acids with Propiophenones through a Combination of Decarboxylation and Dehydrogenation, *Chem. Eur. J.*, 2012, **18**, 8032-8036.

[6] X. Xu, X. Zhang, W. Liu, Q. Zhao, Z. Wang, L. Yu and F. Shi, Synthesis of 2-Substituted Quinolines from Alcohols, *Tetrahedron Lett.*, 2015, **56**, 3790-3792.

[7] B. Ghao, Z. Han, W. Meng, X. Feng and H. Du, Asymmetric Reduction of Quinolines: A Competition between Enantioselective Transfer Hydrogenation and Racemic Borane Catalysis, *J. Org. Chem.*, 2023, **88**, 3335-3339.

[8] S. De, P. Ranjan, V. Chaurasia, S. Pal, P. Pandey and J.K. Bela, Synchronous Proton-Hydride Transfer by a Pyrazole-Functionalized Protic Mn (I) Complex in Catalytic Alcohol Dehydrogenative Coupling, *Chem. Eur. J.,* 2023, **29**, e202301758.

[9] H. Rakasekaran, D.P. Dorairak, N. Bhuvanesh and R. Karvembu, Ru(II)-*p*-Cymene Complexes Containing Hydrazone Ligands Catalyzed: A Alkylation of Letones and One-pot Synthesis of Bioactive Quinolines and 3-(Quinolin-2-yl)-2H-chromen-2-one, *J. Organomet. Chem.*, 2023, **987-988**, 122622-122631.

[10] D. Bhattacharyya, P. Adhikari, K. Deori and A. Das, Ruthenium Pincer Complex Catalyzed Efficient Synthesis of Quinoline, 2-Styrylquinoline and Quinazoline Derivatives via Acceptorless Dehydrogenative Couplings Reactions, *Catal. Sci. Technol.*, 2022, **12**, 5695-5702.

[11] X. Shi, Q. Zhang, A. Wang and T-S. Shang, Substrate-Induced Synthesis of Coumarin-Fused Quinolinones from Anilines, 4-Hydroxycoumarins and DMSO under Air, *Adv. Synth. Catal.*, 2018, **360**, 2691-95.

[12] A.C. Shaikh, D. S. Ranade, S. Thorat, A. Maity, P. P. Kulkarni, R. G. Gonnade, P. Munshid and N. T. Patil, Highly Emmisive Organic Solid with Remarkably Broad Color Turnability Bases on *N,C*-Chelate, Four-Coordinate Organoborons, *Chem. Commun.*, 2015, **51**, 16115-16118.

[13] Q. Ghao, Y. Guo, Z. Sun, X. He, Y. Gao, G. Fan, P. Cao, L. Fang, S. Bai and Y. Jia, Deaminative Cyclization of Tertiary Amines for the Synthesis of 2-Arylquinoline Derivatives with Nonsubstituted Vinylene Fragment, *Org. Lett.*, 2023, **25**, 109-114.

[14] S. Chun, R.R. Putta, J. Hong, S.H. Choi, D-C. Oh and S. Hong, Iron-Catalyzed Transfer Hydrogenation: Divergent Synthesis of Quinolines and Quinolones from *ortho*-Nitrobenzyl Alcohols, *Adv. Synth. Catal.,* 2023, **365**, 3367-3374.

[15] Y. Hu, J. Nan, J. Yin, G. Huang, X. Ren and Y. Ma, Rhodium-Catalyzed Dehydrogenative Annulation of *N*-Arylmethanimines with Vinylene Carbonate for Synthesizing Quinolines, *Org. Lett.*, 2021, **21**, 8527-8532.

[16] *Gaussian 16, Revision C.01*, M. J. Frisch, G. W. Trucks, H. B. Schlegel, G. E. Scuseria, M. A. Robb, J. R. Cheeseman, G. Scalmani, V. Barone, G. A. Petersson, H. Nakatsuji, X. Li, M. Caricato, A. V. Marenich, J. Bloino, B. G. Janesko, R. Gomperts, B. Mennucci, H. P. Hratchian, J. V. Ortiz, A. F. Izmaylov, J. L. Sonnenberg, D. Williams-Young, F. Ding, F. Lipparini, F. Egidi, J. Goings, B. Peng, A. Petrone, T. Henderson, D. Ranasinghe, V. G. Zakrzewski, J. Gao, N. Rega, G. Zheng, W. Liang, M. Hada, M. Ehara, K. Toyota, R. Fukuda, J. Hasegawa, M. Ishida, T. Nakajima, Y. Honda, O. Kitao, H. Nakai, T. Vreven, K. Throssell, J. A. Montgomery, Jr., J. E. Peralta, F. Ogliaro, M. J. Bearpark, J. J. Heyd, E. N. Brothers, K. N. Kudin, V. N. Staroverov, T. A. Keith, R. Kobayashi, J. Normand, K. Raghavachari, A. P. Rendell, J. C. Burant, S. S. Iyengar, J. Tomasi, M. Cossi, J. M. Millam, M. Klene, C. Adamo, R. Cammi, J. W. Ochterski, R. L. Martin, K. Morokuma, O. Farkas, J. B. Foresman, and D. J. Fox, Gaussian, Inc., Wallingford CT, 2019.

[17] Zhao, Y.; Truhlar, D. G. *Theor. Chem. Acc.,* **2008***, 120,* 215-241.

[18] S. A. V. Marenich, C. J. Cramer, D. G. Truhlar, *J. Phys. Chem. B*, **2009**, *113,* 6378-6396.
